# Effect of immune-related intratumoral microbiota and host gene expression on cancer prognosis

**DOI:** 10.1128/msystems.01146-25

**Published:** 2025-09-15

**Authors:** Qingzhen Fu, Ning Zhao, Xia Li, Yanbing Li, Tian Tian, Lijing Gao, Yukun Cao, Liwan Wang, Jinyin Liu, Fan Wang, Yanlong Liu, Binbin Cui, Yashuang Zhao

**Affiliations:** 1 Department of Epidemiology, School of Public Health, Harbin Medical University 34707, Harbin, P.R. China; 2 Department of Colorectal Surgery, Harbin Medical University Cancer Hospital 91631, Harbin, P.R. China; The University of Hong Kong, Hong Kong, Hong Kong

**Keywords:** intratumoral microbiota, host gene expression, TCGA, prognosis, immunity, survival mediation association, single cell

## Abstract

The intratumoral microbiota has been identified as an indispensable part of the tumor microenvironment (TME). However, the relationship between the intratumoral microbiota and host gene expression, as well as its impact on prognosis and TME immunity, remains unclear. We utilized a machine learning-based framework to identify microbiota-host gene associations across 14 tumors from The Cancer Genome Atlas (TCGA) and validated them in 11 tumors from the Gene Expression Omnibus. By calculating immune scores and identifying immune-related microbiota, we developed both a pan-cancer Immune and Prognosis-Related Microbial Score (IPRMS) and cancer-specific IPRMSs and analyzed the relationship between the cancer-specific IPRMSs and immune infiltration at bulk level and single-cell level. Furthermore, we systematically analyzed the potential mechanisms in which the intratumoral microbiota might affect prognosis using survival mediation analyses (SMAs). We identified gene subsets associated with microbiota, which were predominantly enriched in immune-related and cell signaling regulation pathways. Subsequently, we constructed the overall survival-related IPRMS and found that high-IPRMS patients had poorer prognosis in pan-cancer and increased presence of macrophage and cancer-associated fibroblasts. In contrast, low-IPRMS patients showed enrichment in tumor‐infiltrating lymphocytes. SMAs suggest that intratumoral microbiota may influence prognosis by affecting immune cells, pathways, and host genes. High-IPRMSs were consistently associated with poorer prognosis and lower abundance of tumor-infiltrating lymphocytes. At the single-cell level, cancer-associated fibroblasts were predominantly enriched in the high-IPRMS group, while tumor-infiltrating lymphocytes were also mainly enriched in the low-IPRMS group. Our research indicates that the intratumoral microbiota was associated with immune and prognosis, which may impact the cancer prognosis by modifying immune cells, pathways, and host gene expression.

**IMPORTANCE:**

The intratumoral microbiota is a vital part of the tumor microenvironment, yet its interplay with host gene expression and immune regulation remains unclear. Based on a machine learning framework for the interaction analysis of intratumoral microbiota and host genes, as well as the construction of the Immune and Prognosis-Related Microbial Score, our findings suggest that intratumoral microbiota may influence gene expression by affecting host pathways, especially immune-related pathways. Moreover, immune-related intratumoral microbiota are significantly associated with patient survival and TME immunity and may influence prognosis by affecting immune cells, pathways, or gene expression, offering new perspectives and potential biomarkers for predicting personalized patient prognosis in the future.

## INTRODUCTION

Cancer is a disease of complex etiology, frequently resulting from an interplay of genetic predispositions and environmental influences (1). Microbiota, as a common environmental factor, is crucial for supporting human physiological functions and promoting health when the host’s microbiota maintains a state of eubiosis. Conversely, microbial dysbiosis contributes significantly to the development of various diseases, including cancer (2, 3). The associations between gut microbiome and cancer have been widely investigated (4). Recently, intratumoral microbiota, which refers to the community of microbes (including bacteria, viruses, and fungi) that colonizes the tumor microenvironment (TME) (5), has been identified as an essential element of the TME (6). Their relationship with tumors has also received widespread attention.

Research based on *in vivo* and *in vitro* experiments indicates that intratumoral microbiota is closely related to tumors, especially regarding the TME immunity and host prognosis. One of the most researched microbes is *Fusobacterium nucleatum*, which can regulate host gene expression and influence the TME (7, 8). Recent research has shifted focus on populations. In 2019, studies showed that intratumoral microbiota, including *Pseudoxanthomonas*, *Streptomyces*, *Saccharopolyspora*, and *Bacillus clausii*, are associated with long-term survival in patients with pancreatic cancer and potentially modulating immune infiltration and CD8^+^T cells activation (9). Almost simultaneously in 2020, Nejman et al. (10) found that cancers, such as lung cancer and breast cancer (BRCA), previously thought to be sterile, also contained microbiota, and the compositions of intratumoral microbiota varied across different tumors. In addition, they discovered that microbiota reside in both cancer cells and immune cells within these tumor tissues (10). This suggests that intratumoral microbiota may interact with immune cells and influence the host TME. In 2022, Qiao et al. (11) suggested that the intratumoral bacterial load within nasopharyngeal carcinoma tissues was related to the poor survival and negatively correlated with T-cell infiltration of nasopharyngeal carcinoma patients, while other elements, like gene expression, can also affect cancer prognosis. Until now, studies on the intratumoral microbiota in populations generally focus only on the relationship between microbiota and prognosis. The connection between intratumoral microbiota and host gene expression, as well as the impact of this connection on prognosis and TME immunity, remains unclear.

To address this gap, we integrated multi-omics data to systematically analyze the interplay between the intratumoral microbiota and host gene expression. Utilizing a machine learning (ML)-based framework (12), we first identified microbiota-gene associations across 14 tumors in TCGA and validated these associations in the Gene Expression Omnibus (GEO). Subsequently, by constructing a pan-cancer Immune and Prognosis-Related Microbial Score (IPRMS), we assessed the prognostic significance of IPRMS and used survival mediation analysis (SMA) to explore its impact on host prognosis through immune cell infiltration, microbe-related pathways, and gene expression. Finally, we developed cancer-specific IPRMSs, examined their prognosis and TME immunity associations, and validated immune infiltration differences between high-IPRMS and low-IPRMS groups using external GEO data at both bulk and single-cell levels.

## RESULTS

### Identification of intratumoral microbiota-host gene subset associations and pathway enrichment

**Fig 1:**
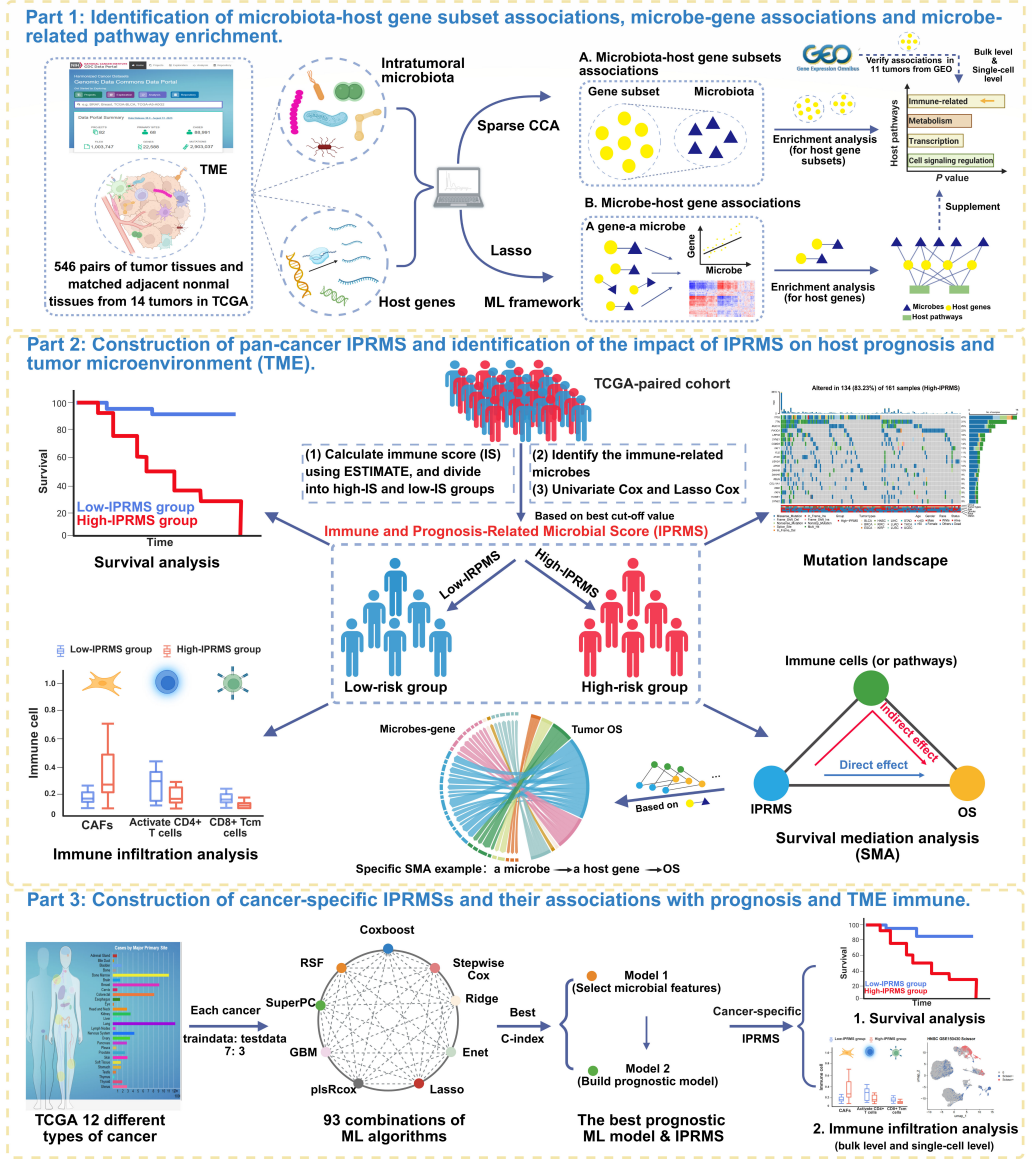
The workflow of this study. Part 1: Based on a ML framework, we applied sparse CCA to identify the microbiota-host gene subset associations across different tumors in TCGA and then used enrichment analysis on these gene subsets to identify microbiota-related host pathways (group-level associations), and we verified these associations in six tumors from GEO. Lasso was applied to identify individual microbe-host gene associations, followed by enrichment analysis on these associations. Part 2: We next calculated the IS based on ESTIMATE algorithm, classified samples from TCGA-paired cohort into high-IS and low-IS groups, and identified immune-related microbiota by difference analysis between two IS groups. Using univariate Cox and Lasso Cox, we developed a pan-cancer IPRMS. By dividing the samples into high-IPRMS and low-IPRMS groups based on the optimal cutoff value, we explored differences in prognosis, immunity, and gene mutations. In addition, SMA was used to explore the potential ways through which microbes affect prognosis, such as whether some microbes can influence host prognosis by affecting specific immune cells or specific pathways. We ultimately expanded our SMA to explore whether a microbe can affect host prognosis by influencing the related gene identified in part 1. Part 3: Through 93 mL combinations, we selected the model with the highest c-index for each cancer. Based on these models, we developed cancer-specific IPRMSs and analyzed the associations between IPRMSs and prognosis and identified the different immune infiltration between two IPRMS groups at both the bulk and single-cell levels. The figure is made by BioRender.

**Fig 2:**
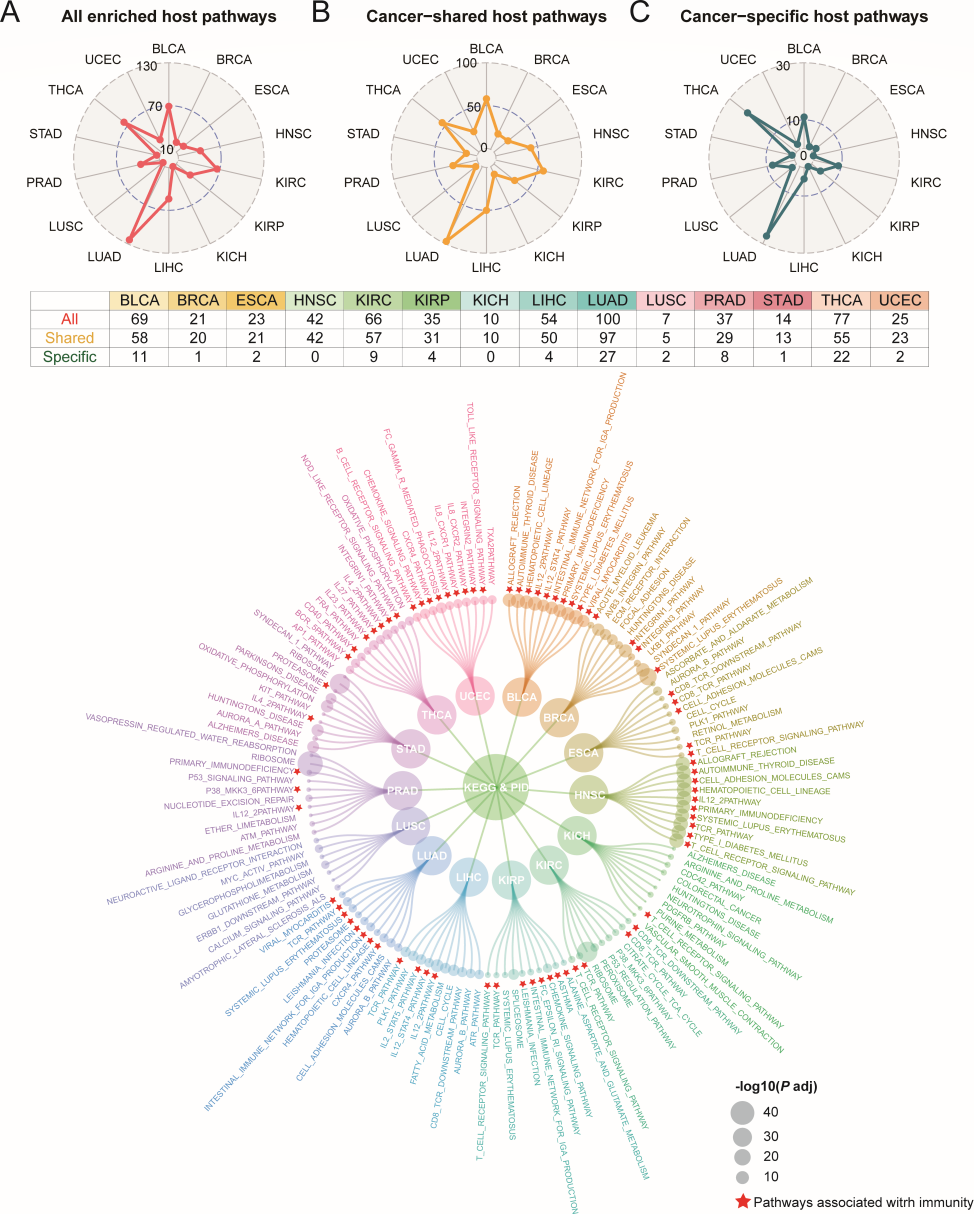
Identification of host pathways associated with intratumoral microbiota. (**A–C**) The distribution of (**A**) overall host pathways, (**B**) cancer-shared host pathways, and (**C**) cancer-specific host pathways enriched for gene subsets correlated to intratumoral microbiota across in 14 tumors; the table shows the specific number of overall enriched pathways, cancer-shared pathways, and cancer-specific pathways in each tumor. (**D**) The top 10 pathways enriched in each tumor with the most significant *P* values; red pentagrams represent labeled pathways related to immunity. BLCA: bladder urothelial carcinoma, BRCA: breast cancer, ESCA: esophageal carcinoma, HNSC: head and neck squamous cell carcinoma, KICH: kidney chromophobe, KIRC: kidney renal clear cell carcinoma, KIRP: kidney renal papillary cell carcinoma, LIHC: liver hepatocellular cancer, LUAD: lung adenocarcinoma, LUSC: lung squamous cell carcinoma, PRAD: prostate cancer, STAD: stomach adenocarcinoma, THCA: thyroid carcinoma, UCEC: uterine corpus endometrial carcinoma.

Figure 1 illustrates the workflow of our study. Firstly, in BRCA, kidney chromophobe (KICH), kidney renal clear cell carcinoma (KIRC), and thyroid carcinoma (THCA), we found statistically significant overall correlations between intratumoral microbiota and host gene expression (*M*^2^_min_=0.728, *P* < 0.05) (Fig. S1; Table S1). In contrast, the overall correlations of esophageal carcinoma (ESCA) and head and neck squamous cell carcinoma (HNSC) were not statistically significant (*P* > 0.05, M^2^_min_=0.915), suggesting that there may be an association between host gene expression subsets and intratumoral microbial subsets in these cancers. Subsequently, we further analyzed the associations between subsets of microbiota and host genes and performed pathway enrichment analysis on gene subsets (FDR < 0.05). Across 14 tumors, a total of 604 microbiota-related pathways were identified (Table S3), including 511 cancer-shared pathways and 93 cancer-specific pathways (Fig. 2A through C). Lung adenocarcinoma (LUAD) exhibited the highest number of pathways with 100 identified. For cancer-specific pathways, LUAD enriched 27 pathways, whereas HNSC and KICH showed no enrichment in any pathways (Fig. 2A through C). Pathways enriched in more than seven tumors were all immune-relevant pathways, including T-cell receptor (TCR) signaling pathway, interleukin (IL) 12-2 pathway, CD8 TCR downstream pathway, and TCR pathway. The 10 most significant pathways based on *P* value in each tumor were mainly immune regulation-related pathways in TME (e.g., CD8 TCR and BCR pathways). Others included cell cycle regulation and signal transduction pathways (e.g., cell cycle and cell adhesion molecules pathways), metabolism-related pathways (e.g., arginine and proline metabolism pathway), and transcriptional and translational-related pathways (e.g., ribosome pathway) (Fig. 2D).

### Validation of bulk-level microbiota-host gene subset associations in GEO

In GEO, we found that the overall correlations between microbiota and genes in bladder urothelial carcinoma (BLCA), BRCA, KIRC, liver hepatocellular cancer (LIHC), LUAD, prostate cancer (PRAD), THCA, and uterine corpus endometrial carcinoma (UCEC) were not statistically significant (Fig. S2; Table S2). These results suggest the possibility of group-level associations occurring within these tumors. We identified 191 microbiota-related pathways in both GEO and TCGA, with the maximum Pathway Overlap Index (POI, Supplementary materials) reaching 0.929 in HNSC and the minimum at 0.038 in PRAD (Table S4). We also found that among the six tumors with highly consistent enriched pathways (POI > 0.4) in TCGA and GEO, the microbiota-related pathways were enriched in ESCA, HNSC, KIRC, and LIHC and were mainly concentrated in immune-related and inflammatory-related pathways, followed by cell cycle regulation and signal transduction pathways (Table S4).

### Validation of the association between the microbiota-host gene subset at the single-cell level in GEO-LUAD

At the single-cell level, following noise reduction and elimination of potential contaminating microbes, we identified 27,184 cells that potentially co-localized with microbes among a total of 37,409 cells (Fig. S3A and B), with T cells harboring the highest number of microbial sequences (Fig. S3C; Table S5). We further identified seven candidate intratumoral microbes, four of which were found to co-localize with more than 10 host cells (Fig. S3D through G). Notably, *Bacillus*, *Streptomyces*, and *Mycobacterium* were also detected in LUAD data sets from both TCGA and GEO databases. Then, in the T cells, macrophages, B cells, and epithelial cells with the most microbial sequences, the microbiota-related pathways mainly enriched immune-related pathways (e.g., TCR signaling pathway), microbial infection-related pathways (e.g., bacterial invasion of epithelial cells pathway), and energy metabolism-related pathways (e.g., glycerophospholipid metabolism pathway) (Table S6). These findings were consistent with the result of bulk level.

### Identification of individual microbe-host gene associations

After excluding microbe-related genes in adjacent tissues, we identified 7,138 unique host genes associated with specific microbes across 14 tumors. The highest number of microbe-related genes was found in THCA (2,050 genes) and the lowest in ESCA (70 genes) (Fig. S4 and S5A; Table S7). Through the enrichment analysis of these genes, we identified 95 cancer-specific pathways and 27 shared pathways across 12 tumors, primarily involving cell cycle regulation and signal transduction, immune response, metabolism, and tumor development-related pathways (Fig. S5B; Table S8).

### The effect of IPRMS on prognosis and subgroup analysis

**Fig 3:**
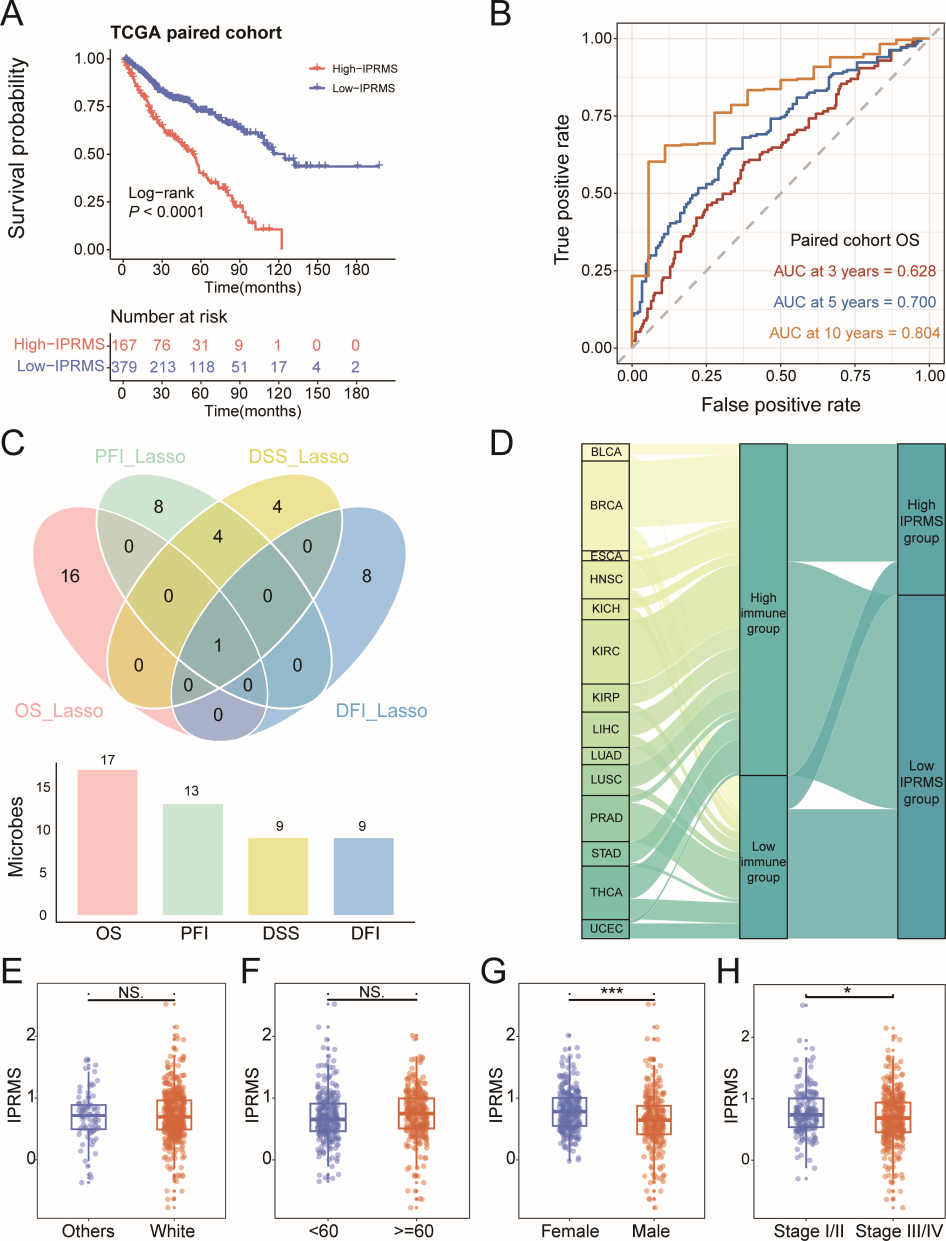
Construction of the pan-cancer IPRMS and survival analysis. (**A**) Based on Lasso Cox algorithm screening out the microbes associated with OS, PFS, DSS, and DFI. (**B**) Kaplan-Meier curves of OS for TCGA-paired cohort based on OS-IPRMS classification. (**C**) Receiver operating characteristic curves of OS for TCGA-paired cohort based on OS-IPRMS classification. (**D**) Sankey diagram showed the IPRMS in different tumor types. (**E–H**) Differences of IPRMS between race, age, gender, and tumor stage. * *P* < 0.05; ** *P* < 0.01; *** *P* < 0.001.

Next, we screened for microbes associated with both immunity and survival in the TCGA-paired cohort. Four microbial subsets associated with immune and four survival metrics were used to construct IPRMSs (Fig. S6; Table S9). We observed less overlap between the microbes associated with the four kinds of survival metrics (Fig. 3A). Compared to low-IPRMS groups, the high-IPRMS groups had poorer prognoses (overall survival [OS]: HR_adj2_ = 2.127 [1.520, 2.977], progression-free interval [PFI]: HR_adj2_ = 2.735 [1.755, 4.264], disease-specific survival [DSS]: HR_adj2_ = 2.111 [1.401, 3.180], disease-free interval [DFI]: HR_adj2_ = 3.309 [1.741, 6.290], *P* < 0.001) (Fig. 3B and C; Fig. S7 and S8; Table 1). However, in the TCGA total cohort, IPRMSs were only statistically significant for OS and DFI (OS: HR_adj2_ = 1.163 [1.032, 1.311]; DFI: HR_adj2_ = 1.358 [1.146, 1.609]) (Table 1; Fig. S8). We used OS-related-IPRMS as the pan-cancer IPRMS to examine the association between immune-related microbiota and prognosis and observed that 31% of patients in the TCGA-paired cohort were classified into the high-IPRMS group (Fig. 3D) via best cut-off values (footnote of Table 1).

**TABLE 1:** The association between IPRMS and four survival metrics in pan-cancer with different adjustment of confounders^*d*^

	Risk group	Univariate cox analysis		Multivariate cox analysis^*a*^		Multivariate cox analysis^*b*^		Multivariate cox analysis^*c*^	
		HR (95% CI)	P	HR_adj1_ (95% CI)	P	HR_adj2_ (95% CI)	P	HR_adj3_ (95% CI)	P
TCGA-paired-cohort-OS	Low	Reference	*P* < 0.001	Reference	*P* < 0.001	Reference	*P* < 0.001	Reference	*P* = 0.023
	High	2.932 (2.186–3.932)		3.293 (2.437–4.450)		2.127 (1.520–2.977)		1.774 (1.083–2.905)	
TCGA-total-cohort-OS	Low	Reference	*P* < 0.001	Reference	*P* < 0.001	Reference	*P* = 0.014	Reference	*P* < 0.001
	High	1.257 (1.126–1.403)		1.224 (1.096–1.367)		1.163 (1.032–1.311)		1.707 (1.467–1.985)	
TCGA-paired-cohort-PFI	Low	Reference	*P* < 0.001	Reference	*P* < 0.001	Reference	*P* < 0.001	Reference	*P* < 0.001
	High	3.288 (2.180–4.961)		3.073 (2.029–4.654)		2.735 (1.755–4.264)		3.169 (1.763–5.696)	
TCGA-total-cohort-PFI	Low	Reference	*P* = 0.973	Reference	*P* = 0.779	Reference	*P* = 0.375	Reference	*P* = 0.641
	High	1.003 (0.866–1.161)		1.022 (0.880–1.186)		1.072 (0.919–1.251)		0.958 (0.802,1.146)	
TCGA-paired-cohort-DSS	Low	Reference	*P* < 0.001	Reference	*P* < 0.001	Reference	*P* = 0.001	Reference	*P* = 0.027
	High	3.730 (2.182–6.374)		3.595 (2.070–6.243)		2.703 (1.509–4.841)		2.346 (1.101–4.998)	
TCGA-total-cohort-DSS	Low	Reference	*P* = 0.327	Reference	*P* = 0.391	Reference	*P* = 0.240	Reference	*P* = 0.265
	High	1.123 (0.890–1.417)		1.108 (0.876–1.401)		1.155 (0.908–1.468)		1.170 (0.888–1.543)	
TCGA-paired-cohort-DFI	Low	Reference	*P* < 0.001	Reference	*P* < 0.001	Reference	*P* < 0.001	Reference	*P* = 0.198
	High	4.106 (2.262–7.452)		4.151 (2.263–7.616)		3.309 (1.741–6.290)		1.767 (0.743–4.203)	
TCGA-total-cohort-DFI	Low	Reference	*P* = 0.001	Reference	*P* = 0.001	Reference	*P* < 0.001	Reference	*P* = 0.001
	High	1.317 (1.119–1.551)		1.305 (1.108–1.537)		1.358 (1.146–1.609)		1.388 (1.143–1.686)	

^*a*^ Multivariate Cox analysis was adjusted for age, gender, race, and stage.

^*b*^ Multivariate Cox analysis was adjusted for age, gender, stage, race, and tumor types.

^*c*^ Multivariate Cox analysis was adjusted for age, gender, stage, race, tumor types, tumor status, chemotherapy, and radiation therapy.

^*d*^ For OS, PFI, DSS, and DFI, the IPRMS cutoff values for defining high-IPRMS and low-IPRMS groups were 0.8825, 0.5484, 0.8641, and −0.0923, respectively, based on the best cutoff of ROC. HR indicates hazard ratio; 95% CI indicates 95% CI of hazard ratio.

We further analyzed the associations between pan-cancer IPRMS and prognosis in various immune subtypes and TME subtypes and found that patients in the high-IPRMS groups had significantly worse prognoses in C3 and C4 subtypes and D subtypes (Fig. S9 and S10). Moreover, survival analysis using IPRMS across 13 tumors demonstrated that the high-IPRMS was a risk factor for patient prognosis in BLCA, BRCA, ESCA, HNSC, KIRC, kidney renal papillary cell carcinoma (KIRP), LIHC, and THCA (*P* < 0.1, Fig. S11). Additionally, the pan-cancer IPRMS was statistically higher in female and stage I/II than that in male and stage III/IV (Fig. 3E through H).

### The mediation effects of host TME factors on the association between intratumoral microbiota and prognosis

**Fig 4:**
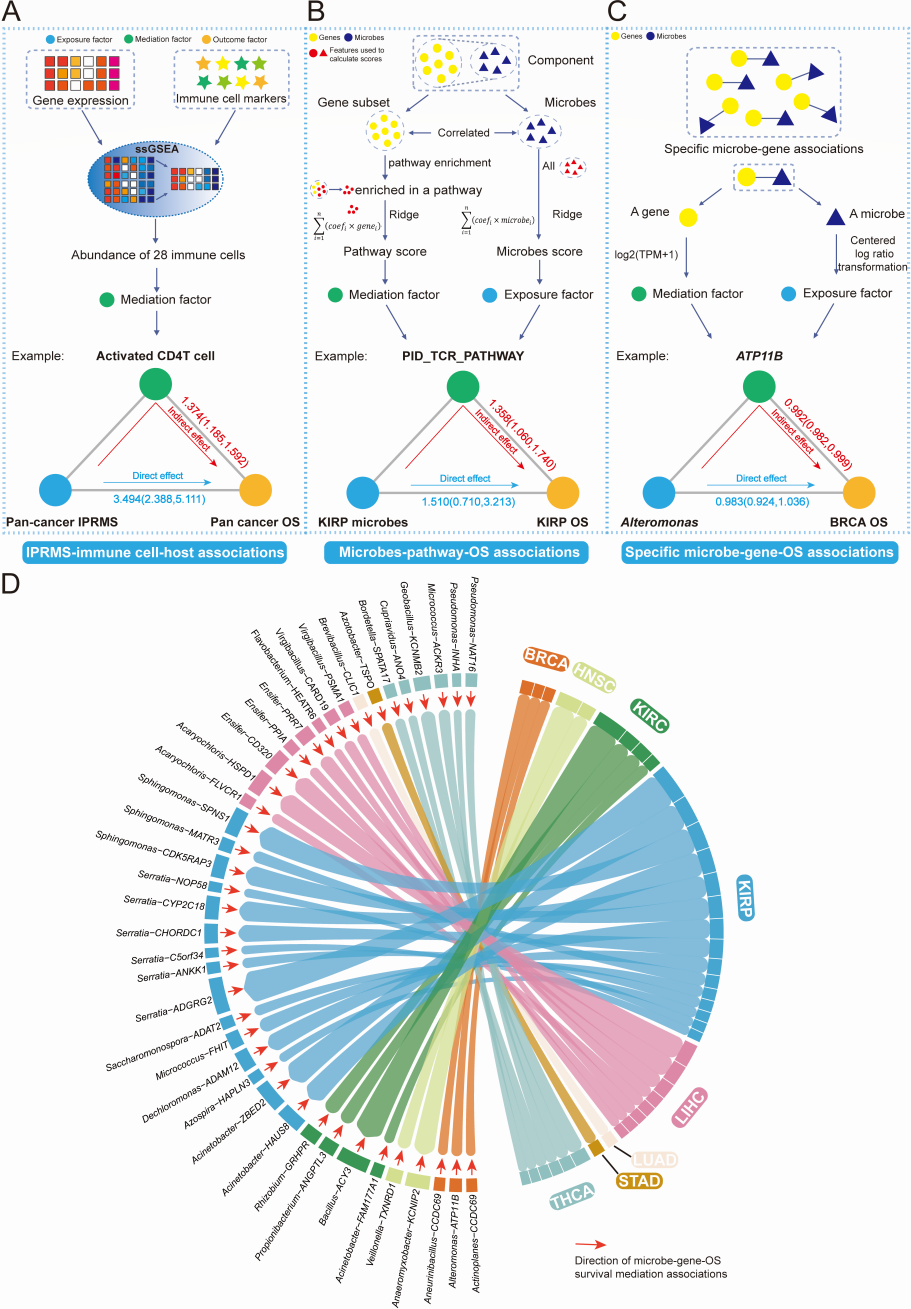
SMA of microbiota affecting host prognosis. (**A–C**) represent analytical processes and examples of the three types of SMAs developed in this study: (**A**) represents microbe subsets influencing host prognosis by affecting infiltration of immune cells (IPRMS-immune cell-OS SMAs); (**B**) describes microbiota-related gene subsets identified based on group-level associations and enriched these genes to some specific pathways to explore whether microbiota can influence host prognosis by affecting these pathways (microbiota-pathway-OS SMAs); (**C**) shows identification of microbe-host gene associations using Lasso and stability selection to investigate whether a particular microbe can influence host prognosis by affecting the expression of related host genes (microbe-gene-OS SMAs). The total effect of microbes affecting host prognosis can be divided into the indirect effect of the microbiota on prognosis by mediating gene expression, pathways, or immune cells, and the direct effect of microbes on prognosis. (**D**) represents the microbe-gene-OS SMAs. Each band represents an instance: a specific microbe affecting the prognosis of cancer by mediating a specific gene expression. The mediation effect relationship was identified through delta method (*P* < 0.05), where the colors of the bars represent the tumor type affected by a particular microbe-host gene-OS association. The width of the band is proportional to the significance level of the mediating effect. The adjusting factors for HR include age, gender, race, and tumor stage.

We further explored whether microbiota can modulate immune cells in TME, microbiota-related pathways, or microbe-related gene expression and thus affect prognosis. Firstly, we used 28 immune cell abundance as mediation factors (Fig. 4A). In the TCGA-paired cohort, the IPRMS-immune cell-OS SMAs demonstrated that microbiota can mediate the prognosis by affecting infiltration of immune cells and may mainly affect infiltration of T cells (IPRMS-activated CD4^+^T [CD4^+^T_ac_] cell-OS SMA: HR_indirect_ = 1.374 [1.185, 1.592], HR_direct_ = 3.494 [2.388, 5.111], and proportion mediated [PM] = 0.344). We also found consistent results in the TCGA-total cohort (IPRMS-CD4^+^T_ac_ cell-OS SMA: HR_indirect_ = 1.163 [1.128, 1.199], HR_direct_ = 1.106 [0.977, 1.252], PM = 0.629) (Fig. 4A; Table S10).

Secondly, we investigated whether microbiota might affect the prognosis by regulating host pathways. The microbes-pathway-OS SMAs revealed that microbiota can also affect prognosis by modulating the immune-related pathways (especially in KIRP, KIRP microbes-CD8 TCR pathway-OS SMAs: HR_indirect_ = 1.343 [1.046, 1.837], HR_direct_ = 1.744 [0.655, 4.220], PM = 0.446), cell cycle regulation and signal transduction pathways, and metabolism-related pathways (Fig. 4B; Tables S11 and S12). Finally, we analyzed whether a microbe could affect the prognosis by altering a microbe-related gene expression. We identified a total of 103 microbe-gene-OS SMAs (using delta method) and 57 microbe-gene-OS SMAs (using bootstrap method) at every taxonomic level (Fig. 4D; Tables S13 and S14). For example, *Alteromonas* was associated with prognosis of BRCA by influencing the *ATP11B* expression, exhibiting a protective role (Fig. 4C and D; Table S13).

### The impact of IPRMS on the immune and genomic mutations of the TME

**Fig 5:**
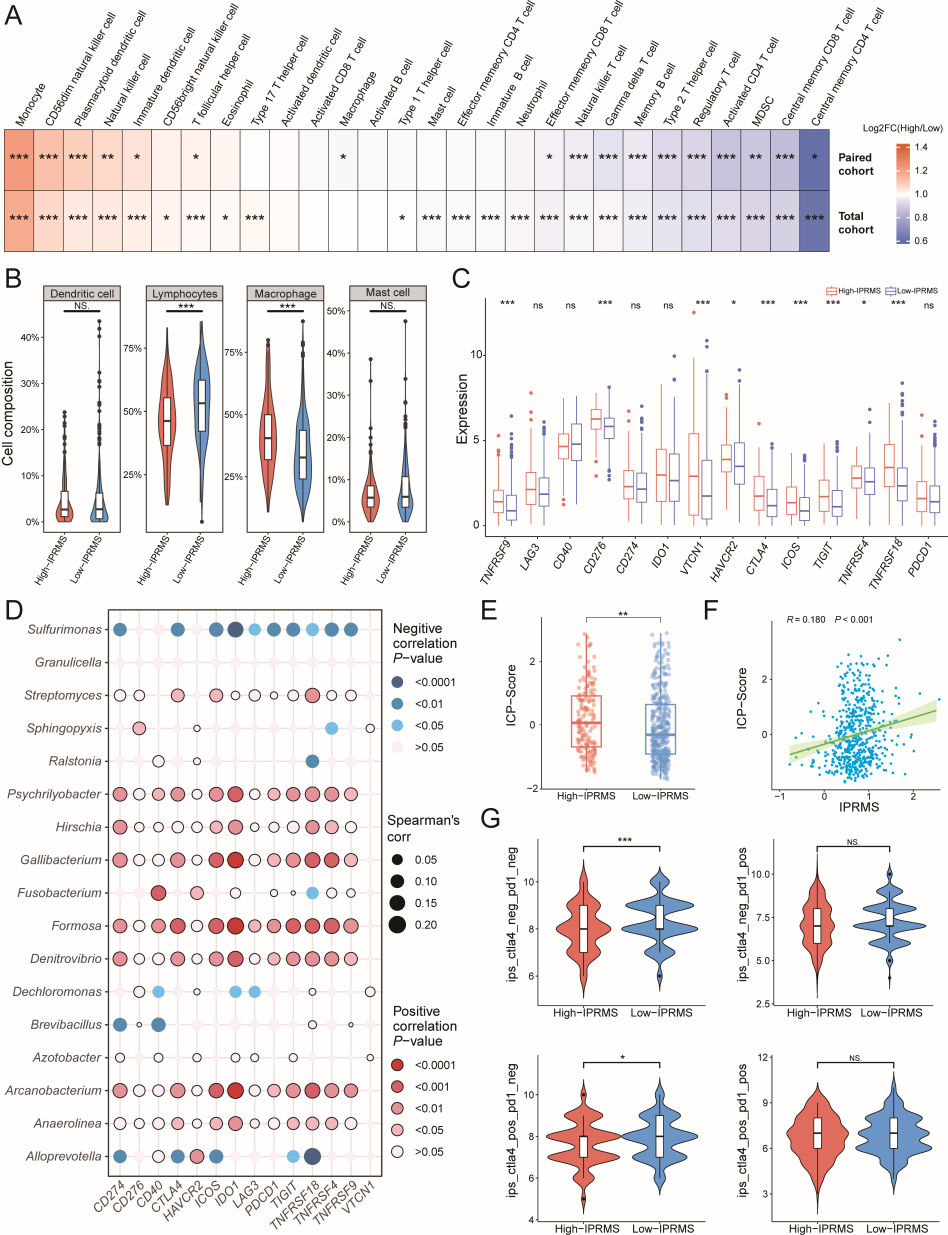
The association between IPRMS and immune characteristics. (**A**) Heatmap shows the ratio of high-IPRMS/low-IPRMS infiltrating estimations of 28 immune cells in TME. The ratio >1 means higher infiltration in high-IPRMS, while the ratio <1 means higher in low-IPRMS (High: high-IPRMS group, Low: low-IPRMS group). (**B**) The differential distribution of dendritic cells, lymphocytes, macrophages, and mast cells between high-IPRMS and low-IPRMS groups in TCGA-paired cohort (based on the results of the CIBERSORT algorithm). (**C**) The differences in ICP gene expression between different IPRMS groups. (**D**) Spearman correlation between the abundance of 17 microbes used to construct IPRMS and the expression of common ICP genes. (**E**) The difference in ICP score between high-IPRMS group and low-IPRMS group. (**F**) The Spearman correlation between IPRMS and ICP score. (**G**) Distribution of IPS among four IPS types (CTLA4^−^ PD1^−^, CTLA4^−^ PD1^+^, CTLA4^+^ PD1^+^, CTLA4^+^ PD1^−^) between high-IPRMS and low-IPRMS group in TCGA-paired cohort. *P < 0.05; **P < 0.01; ***P < 0.001.

To assess the effect of IPRMS on immune infiltration in TME, we estimated 28 immune cells abundance. The low-IPRMS group displayed a higher proportion of tumor‐infiltrating lymphocytes (TILs), including central memory CD4^+^T (CD4^+^T_cm_) cells, CD8^+^T_cm_ cells, and CD4^+^T_ac_ cells (Fig. 5A). CIBERSORT showed the high-IPRMS group showed increased macrophage infiltration but reduced TILs infiltration compared to the low-IPRMS group (Fig. 5B; Fig. S12). Furthermore, the EPIC analysis showed that the low-IPRMS group exhibited higher levels of CD8^+^T cells and endothelial cell infiltration, while cancer-associated fibroblasts (CAFs) and macrophages were less prevalent (Fig. S13).

Subsequently, we investigated the difference and correlation between IPRMS and immune checkpoint (ICP) gene expression, as well as immunotherapy sensitivity. The high-IPRMS group exhibited higher expression of ICP genes (Fig. 5C; Fig. S14), and most microbes constructing pan-cancer IPRMS showed positive correlations with ICP genes expression (Fig. 5D). Additionally, IPRMS was positively correlated with the ICP score (Fig. 5E and F, *r* = 0.180, *P* < 0.001). Based on immunophenotype scores (IPSs), the low-IPRMS group had higher immunogenicity and better immunotherapy efficacy (Fig. 5G; Fig. S15).

As for the association between IPRMS and genomic variation (Fig. S16A and B), we noticed a remarkable positive correlation between IPRMS and tumor mutational burden (TMB), with the high-IPRMS group displaying a substantially higher TMB than the low-IPRMS group (Fig. S16C and D). Furthermore, the high-IPRMS group exhibited a higher mutation frequency, particularly in the top 20 mutated genes (Fig. S16E). TP53 had the highest mutation frequency, with high-IPRMS linked to an increased mutation (odds ratio [OR] = 2.47 [1.65, 3.72]) (Fig. S16E and F). We also found that a greater proportion of patients in the high-IPRMS group had mutations in the TP53 signaling pathway compared to the low-IPRMS group (Fig. S16G through I).

### Cancer-specific IPRMSs and their associations with prognosis and immune infiltration

**Fig 6:**
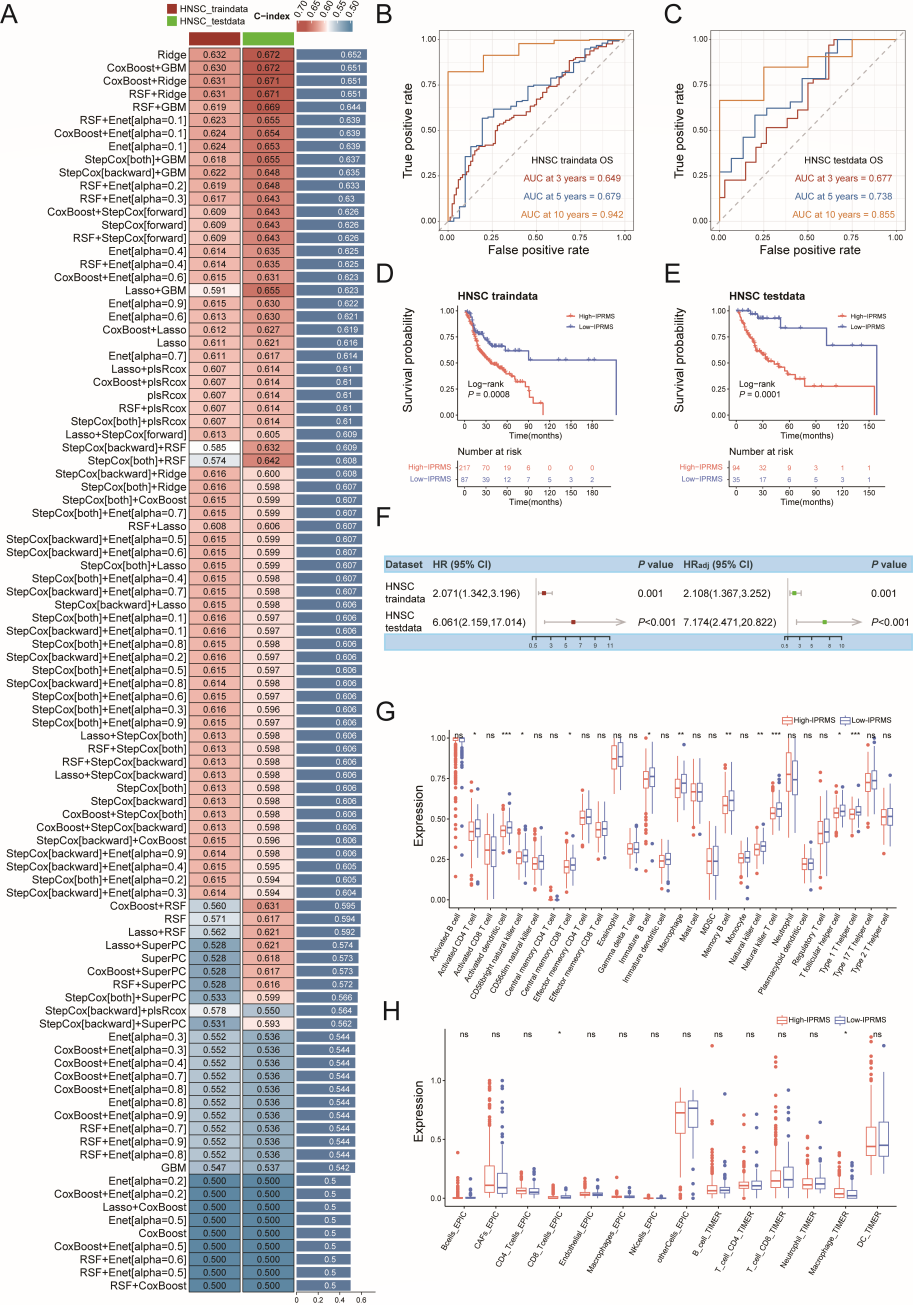
Construction of HNSC-IPRMS and its association with prognosis and immune infiltration. (**A**) The C-index for traindata, testdata, and average C-index of each ML combination in HNSC. (**B–C**) ROC curve of the prognostic model based on IPRMS for (**B**) HNSC traindata and (**C**) testdata. (**D–E**) Associations between IPRMS-HNSC and survival in (**D**) HNSC traindata and (**E**) testdata. (**F**) Univariate and multivariate associations between IPRMS and survival and adjust factors were age, gender, race, and tumor stage. (**G**) The difference of infiltrating estimations of 28 immune cells in TME between high-IPRMS and low-IPRMS groups. (**H**) The difference in abundance of immune cells between high-IPRMS and low-IPRMS groups using the EPIC and TIMER. * *P* < 0.05; ** *P* < 0.01; *** *P* < 0.001.

We developed cancer-specific prognostic prediction models for each cancer (Fig. 6A; Fig. S17 to S27A) and found the microbes used to construct the IPRMSs were cancer-specific, with only four shared microbes (Table S15). The finding, consistent with pan-cancer result, showed that the high-IPRMS groups generally had a poorer prognosis, especially in BRCA, ESCA, HNSC, KIRC, LUAD, lung squamous cell carcinoma (LUSC), THCA, and UCEC (*P*_testdata_ of log-rank test < 0.05) (Fig. 6B through E; Fig. S17 to S27B through E), with the point estimates of HR_adj_ ranging from 1.127 to 7.174 for the testdata (Fig. 6F; Fig. S17 to S27F). Additionally, IPRMS effectively predicted the 10-year survival status of HNSC patients (AUC_testdata_ = 0.855) (Fig. 6B and C), and it also performed well in predicting the 5-year survival status of THCA (AUC_testdata_ = 0.852) and ESCA patients (AUC_testdata_ = 0.776) (Fig. S19B, C, S26B and C). Multivariate Cox further indicated that IPRMSs were associated with the prognosis of HNSC (HR_adj(testdata)_ = 7.174 [2.472, 20.822], *P* < 0.001), LUAD (HR_adj(testdata)_ = 3.881 [1.189, 12.663], *P* = 0.025) and KIRC (HR_adj(testdata)_ = 2.449 [1.160, 5.170], *P* = 0.014). Finally, immune infiltration analysis revealed notable differences in immune cell infiltration between the two IPRMS groups in BRCA, HNSC, KIRC, KIRP, and UCEC. The TILs, especially CD4^+^T cells (CD4^+^T_ac_) and CD8^+^T cells (CD8^+^T_ac_ and CD8^+^T_cm_), were more abundant in low-IPRMS groups.

### ScRNA-seq validated the associations between IPRMSs and immune infiltration

Finally, based on the Scissor algorithm (Supplementary materials) (13), we identified cell subtypes in single-cell RNA-seq (scRNA-seq) data that were most highly associated with the IPRMS phenotype. Consistent with the results of TCGA, the TILs were predominantly enriched in the Scissor− (low-IPRMS) groups (OR > 1.5, *P* < 0.05), such as CD8^+^T cells being enriched in the Scissor− groups of HNSC and KIRC and CD4^+^T cells primarily enriched in HNSC and LUAD. The Scissor+ (high-IPRMS) groups of BRCA, KIRC, and LUAD were mainly enriched with CAFs (OR > 1.5, *P* < 0.05) (Fig. S28 to S31; Table S16).

## DISCUSSION

Recently, the intratumoral microbiota has emerged as a rapidly growing area of research. Previous studies mainly explored the relationship between intratumoral microbiota and tumors through *in vivo* and *in vitro* experiments, lacking population-based research support. Our innovation is that this is the first study to analyze the impact of immune-related intratumoral microbiota on prognosis and immunity of TME, by integrating host gene expression data across TCGA and GEO pan-cancer data sets, at both the bulk and single-cell levels. By analyzing the association between intratumoral microbiota and gene expression, we found that microbiota-related gene subsets in each tumor were primarily enriched in immune-related pathways. Furthermore, after constructing both a pan-cancer IPRMS and cancer-specific IPRMSs, and based on survival analysis, SMAs, and immune infiltration analysis, we found that high-IPRMS groups had a poorer prognosis and lower infiltration of TILs. Ultimately, at the single-cell level, we validated the relationship between IPRMS phenotype and TME immunity.

The intratumoral microbiota is closely associated with gut microbiota. Studies have shown that gut microbiota may be transported through the blood and colonize tumors (14). In this study, we found that after filtering by abundance and prevalence, the intratumoral microbiota mainly originated from the gut (with an average contribution of 95.2%, Fig. S32). However, when the unfiltered intratumoral microbiota data were traced to the source, the proportion of gut microbiota origin decreased (mean contribution of TCGA-paired-cohort = 0.777, mean contribution of TCGA-total-cohort = 0.758; Table S17). Additionally, although both can affect the TME, their mechanisms of action are different: the gut microbiota mainly influences the TME indirectly through metabolic products or by modulating the immune system (15), while the intratumoral microbiota directly acts within the TME, such as inducing DNA damage (16) and regulating signaling molecules (17). This highlights the uniqueness of the intratumoral microbiota, and further research may help reveal its role in cancer progression.

In 2022, Priya et al. (12) employed a ML framework that overcame common problems in microbiota-host gene expression integration, such as high dimensionality and sparsity. They identified biological associations between gut microbiota and host genes, as well as pathways in patients with three intestinal diseases. Consequently, we used Priya’s ML framework to further investigate the associations between intratumoral microbiota and gene subsets and identified microbiota-related pathways. We discovered that the TCR signaling pathway was the most frequently enriched microbiota-related pathway, and it was even identified at the single-cell level of LUAD. Previous studies have shown a strong link between intratumoral microbiota and T cells in the TME. Based on population study and animal experiment, Peng et al. (18) found that *Methylobacterium* in stomach adenocarcinoma (STAD) tissue was significantly associated with poor prognosis in STAD patients and could decrease the CD8^+^ tissue-resident memory T cells in the TME. Using scRNA-seq, Ghaddar et al. (19) observed that T cells with cell-associated bacteria in pancreatic cancer tissue were more likely to have an activated phenotype (e.g., memory T cells or effector T cells), and these T cells had upregulated pathways for PD-1 signaling and responses to intracellular infections. These studies, along with our findings, suggest a close connection between the intratumoral microbiota and T cells, and intratumoral microbiota may induce immune infiltration and immune response in the TME. Additionally, the DNA replication pathway was enriched in HNSC, LUAD, PRAD, and THCA, indicating that microbiota may induce DNA damage by interfering with DNA replication. In summary, these findings highlight the cross talk between TME and intratumoral microbiota, suggesting close associations among microbiota, TME immunity, and prognosis.

Risk scores derived from multiple intratumoral microbiota have been successfully used to predict cancer prognosis (20–23). Alexander et al. (22) predicted a higher disease-free survival in colorectal cancer (CRC) resected patients based on a model that included a group of microbes such as *Fusobacterium* and *Gemella*. Song et al. (23) constructed a risk score using 27 microbes, which serves as an independent prognostic indicator for hepatocellular carcinoma patients. However, these risk scores considered only microbial features and did not integrate host-level information. Guan et al. (21) identified microbial modules linked to gene expression across pan-cancer using weighted gene coexpression network analysis. They discovered genes and pathways related to these microbes and developed prognostic models that integrated both microbial and host gene features. However, they did not exclude genes and pathways associated with adjacent tissues. Moreover, these models were relatively complex due to the integration of both gene and microbe features. In this study, we calculated immune score (IS) and identified immune-related microbes in high-IS and low-IS groups, ultimately constructing the IPRMS. Thus, our risk score incorporated immune properties. We also observed that high-IPRMS patients exhibited worse prognosis, even across different immune and TME subtypes. Given significant heterogeneity exists among various tumor types, with variations observed in both the types and abundance of intratumoral microbiota (10), we developed cancer-specific IPRMSs and found that high-IPRMS groups had poor prognoses in eight tumors. In conclusion, the IPRMS, developed based on the connection between intratumoral microbiota and host, may serve as an effective prognostic biomarker.

Unlike previous studies, we explored whether microbes influence prognosis by host TME factors via SMA, and we identified multiple microbe-host gene-OS SMA associations. Firstly, we discovered that intratumoral microbiota can impact prognosis by modulating immune cells (e.g., CD4^+^T_ac_ cells, CD8^+^T_cm_ cells, and memory B cells) or host pathways (e.g., CD8 TCR pathway and IL12-2 pathway). Studies have indicated that microbial invasion can activate cancer-associated pathways, such as PI3K signaling pathway (24, 25), and can also cause inflammation or local immune suppression to promote tumor growth, by affecting the differentiation and function of immune cells in the TME (24, 26, 27). For example, bacteria in CRC tissue induce IL-17 production and promote the influx of B cells, thereby promoting tumor progression (28–30). Intratumoral microbiota can influence host gene expression, such as *TP53*, *ERK*, and *CYP2J2*, thereby promoting tumor progression (31, 32). Our findings suggest that when intratumoral microbiota colonize the TME, they may influence the prognosis by affecting the infiltration of immune cells, regulating host pathways, and gene expression.

Finally, we observed that the low-IPRMS groups exhibited a higher abundance of TILs compared to the high-IPRMS groups, especially CD4^+^T and CD8^+^T cells. Recently, a study indicated that some bacteria can activate T cells to elicit anti-tumor immunity: *Lactobacillus reuteri* selectively activates the aryl-hydrocarbon-receptor signaling pathway in CD8^+^T cells by secreting indole-3-carboxaldehyde, enhancing IFN-γ production and thereby eliminating tumor cells (33). We also found that the high-IPRMS group had a higher frequency of TP53 mutation and TP53 signaling pathway gene mutation, but the specific mechanism remains unclear. Kadosh et al. (34) found that the microbial metabolite gallic acid can transform the tumor suppressor function of mutant TP53 into a tumor-promoting effect, ultimately leading to uncontrolled progression of CRC tumors. Furthermore, *Helicobacter pylori* (*Hp*) can inhibit USF1/p53 nuclear interaction and impairs DNA repair function (35). Our research also suggests potential correlation among intratumoral microbiota, CAFs, and prognosis. The CAFs, which secrete immune-regulatory molecules and interact with immune cells, can remodel the extracellular matrix, facilitating cancer progression and immune evasion (36, 37). In STAD, *Hp* infection of STAD-associated fibroblasts has been shown to promote the expression and secretion of Serpin family E member 1 (Serpin E1) by CAFs. This interaction subsequently induces Serpin E1 expression in STAD cells, thereby enhancing p38 mitogen activated protein kinase/vascular endothelial growth factor A (MAPK/VEGFA)-mediated angiogenesis, which contributes to STAD cell growth and peritoneal metastasis (38). In CRC, microbial dysbiosis leads to reduced production of butyrate, a key metabolite of the gut microbiota. This deficiency increases the abundance of SULF1^+^ CAFs, which are associated with poor clinical outcomes in CRC patients, ultimately promoting tumor progression (39). *Actinomyces* co-localize with CAFs in CRC tissues, and their interaction with gram-negative bacteria can activate the TLR2/NF-κB pathway, leading to the suppression of CD8^+^T cell infiltration within TME (40). In addition, Maddalena et al. (41) demonstrated that p53 mutations may worsen the prognosis of cancer patients by promoting TME fibrosis and inhibiting the immune system from killing cancer cells. These studies, combined with our findings, suggest that the enrichment of CAFs in the high-IPRMS group and their association with TP53 mutations and intratumoral microbiota may be linked to poor prognosis. Therefore, our finding suggests that the microbiota in the high-IPRMS group may be involved in the somatic mutations, shaping an immunosuppressive microenvironment devoid of TILs, facilitating the immune escape of tumor cells, and consequently contributing to poor prognosis.

This study has limitations. Firstly, there is a lack of large-scale data sets that simultaneously include prognosis information, intratumoral microbiota, and host gene expression to validate the IPRMS. Secondly, the TCGA data set may contain potential environmental contaminants, and the mechanisms of microbes in relation to tumor progression remain unclear. Nevertheless, the microbial data we used were mainly based on the remaining microbes after strict filtering based on sample prevalence and minimum relative abundance, and more than 95% of these were detected in the gut microbiome data set (42). In addition, some microbes mainly found in water or soil may enter the body through ingestion of food or other routes and reach tumor tissues, potentially contributing to cancer development. However, further research is needed to clarify the causal relationships and underlying mechanisms. Thirdly, the taxonomic resolution of the TCGA microbial data were limited to the genus level; further work with the species level is needed. In the future, IPRMS can be applied to multicenter large-sample prospective cohort studies to verify its correlation with survival. Moreover, clinical trials of probiotics or antibiotics targeting the intratumoral microbiota can be conducted in different IPRMS groups to evaluate whether regulating the IPRMS-related microbiome can affect the response to immunotherapy and improve survival rates.

### Conclusion

Our findings suggest that intratumoral microbiota may influence gene expression by affecting host pathways, especially immune-related pathways. Moreover, immune-related intratumoral microbiota are significantly associated with patient survival and immune in TME and may influence prognosis by affecting immune cells, pathways, or gene expression, offering new perspectives and potential biomarkers for predicting patient personalized prognosis in the future.

## MATERIALS AND METHODS

### Data accession

The intratumoral microbial data, derived from TCGA-miRNA-seq data, were sourced from the Bacteria in Cancer (BIC) database (http://bic.jhlab.tw/) (43). There are two main reasons for selecting microbial data from miRNA-seq: miRNAs are transcribed in both bacteria and host, and miRNAs have been found to play a regulatory role in bacteria and bacterial infectious diseases (44, 45). Most bacterial RNAs without poly-A tails are filtered out in RNA-seq, whereas miRNA-seq that has not processed poly-A filtering can identify bacteria not found in RNA-seq (46).

We selected 14 tumors from the BIC database for subsequent analysis, and the inclusion criteria for tumor selection were as follows: with both tumor tissues and adjacent normal tissues, and after matching microbial data with host RNA-seq data, there were >10 pairs of adjacent tissues and tumor tissues. Ultimately, BLCA, BRCA, ESCA, HNSC, KICH, KIRC, KIRP, LIHC, LUAD, LUSC, PRAD, STAD, THCA, and UCEC were selected for further analysis. The RNA-seq data were downloaded from the Genomic Data Commons (GDC) repository (https://portal.gdc.cancer.gov/). Clinical and survival data were available at the University of California, Santa Cruz Xena database (https://xenabrowser.net/datapages/). The 546 pairs of tumor tissue and adjacent normal tissues across the 14 tumors were defined as TCGA-paired cohort, while all samples from the 14 tumors (5,838 samples) constituted the TCGA-total cohort in this study.

Additionally, we obtained bulk RNA-seq data from GEO for 11 tumor types, including BLCA, BRCA, ESCA, KIRC, LIHC, and LUAD, and used the raw data to identify intratumoral microbiota. In addition, we also used a scRNA-seq raw data set of LUAD to explore the association between intratumoral microbiota and host gene expression. Moreover, we analyzed processed scRNA-seq data for BRCA, HNSC, KIRC, and LUAD from Tumor Immune Single-cell Hub 2 (TISCH2, https://xenabrowser.net/datapages/) (47). The data from GEO and TISCH2 were used to validate the results from TCGA. Details of the data collection for validation, processing, and analysis were provided in the Supplementary materials.

### Identifying microbiota-host associations based on a ML framework

Firstly, we analyzed the overall correlations between intratumoral microbiota and host gene expression using Procrustes analysis and the Mantel test, quantifying correlations with *M*^2^ and *r* values (Supplementary materials). Secondly, we identified microbiota-host gene subset associations between tumor tissues and adjacent normal tissues in the TCGA-paired cohort using Priya’s ML framework (12) (Supplementary materials). In short, we fitted the Sparse Canonical Correlation Analysis (CCA) model to each tumor to identify subsets of significantly correlated genes and intratumoral microbiota (group-level associations). Then, for each gene subset with FDR < 0.05, we performed pathway enrichment analysis, and after excluding pathways enriched in the adjacent tissues, we identified host pathways associated with the intratumoral microbiota in each tumor. We further categorized pathways into two types: the “cancer-sharing” pathways, which were enriched in at least two tumors, and the “cancer-specific” pathways, which were related to the intratumoral microbiota in only one tumor. Lasso-penalized regression was used to identify distinct correlations between individual host gene and intratumoral microbe, and both the “shared” microbe-host gene associations and “cancer-specific” microbe-host gene associations were identified. The microbiota-host associations at the single-cell level are detailed in the Supplementary materials.

### Construction of pan-cancer IPRMS and analyzing the association between IPRMS and prognosis

We used the ESTIMATE algorithm (48) to calculate the IS and divided 546 tumor samples with OS >30 days into high-IS and low-IS groups according to the optimal cut-off IS (2); We then performed difference analysis between the two IS groups to identify immune-related microbes (*P* < 0.1) (3); univariate Cox analysis was used to initially select immune-related microbes connected to OS (*P* < 0.1) (4). These microbes were further selected through Lasso Cox and used to develop the IPRMS with the following formula:


$$IPRMS=\sum_{i=1}^{n}\left({coef}_{i}\times {microbe}_{i}\right)$$

The analysis for PFI, DFI, and DSS followed the same process as for OS.

After dividing into high-IPRMS and low-IPRMS groups, we assessed the associations between pan-cancer IPRMS with OS, PFI, DFI, and DSS, respectively. Subgroup analyses were conducted based on immune subtypes, including C1 (wound healing), C2 (IFN-γ dominant), C3 (inflammatory), C4 (lymphocyte depleted) (49), and TME subtypes, such as immune-enriched (IE, non-fibrotic tumor), IE/F (IE, fibrotic tumor), F (fibrotic tumor), and D (depleted tumor) (50).

### SMA of microbiota affecting host prognosis

We investigated the effect of intratumoral microbiota on prognosis by analyzing whether microbiota affected immune cells (defined as IPRMS-immune cell-OS SMAs), microbiota-related pathways identified from group-level associations (microbiota-pathway-OS SMAs), or microbe-related gene identified from individual microbe-host gene associations (microbe-gene-OS SMAs), using VanderWeele’s SMA. The total effect of microbiota on prognosis was decomposed into indirect effect (HR_indirect_) and direct effect (HR_direct_) (Supplementary materials).

### The effect of IPRMS on immune and genomic variation in the TME

We employed ssGSEA (51) to evaluate the relative proportion of 28 immune cells and also used CIBERSORT (52), EPIC (53), and TIMER (54) algorithms to analyze immune infiltration between two IPRMS groups. Based on CIBERSORT results, we divided immune-infiltrating cells into four major categories (55). In addition, we analyzed the associations among the expression of common ICP genes, the ICP score (calculated using the “Immuno-Oncology Biological Research, IOBR” package) (56), and IPRMS. Utilizing the IPS data from The Cancer Immunome Atlas (https://tcia.at/home) (57), we predicted the therapeutic effects of CTLA-4 and PD-1 ICP inhibitors in patients. Furthermore, using the R package “maftools” (58), we summarized the overall mutation status and calculated the TMB for both IPRMS groups.

### Construction of tumor-specific IPRMSs

We randomly divided each TCGA tumor tissue into a traindata (70%) and a testdata (30%), and employed 9 mL algorithms, including Cox boost, stepwise Cox, Lasso Cox, Ridge, Elastic Net (Enet), survival support vector machine, generalized boosting regression models, and partial least Cox and random survival forests (RSF), combining them into 93 mL combinations to construct prognostic models. Then, we calculated the C-index of each model on the traindata and testdata, ranked them based on the average C-index of the two datasets, and selected the model with the highest average C-index as the optimal model. Finally, we developed 12 unique IPRMSs and explored the prognostic and immune differences across various tumors.

### Statistical analyses

Differential microbiota analysis was utilized by “trans_diff” function from the “microeco” package (59), to determine abundance differences of microbiota between two IS groups. Spearman correlation analysis was applied to calculate the correlations between variables. The Wilcoxon rank sum test was employed to compare group differences. Survival differences across IPRMS groups were estimated using the Log-rank test. The optimal cutoff value or the median for IPRMS was determined through a comprehensive analysis using the “surv-cutpoint” function from the “survminer” (60) and “survivalROC” package (61). A two-tailed test was employed, with statistical significance set at *P* < 0.05 unless specified otherwise. All statistical analyses were performed using R v.4.2.2.

## Data Availability

The data sets supporting the conclusions of this article are available in open access (http://bic.jhlab.tw/) from Chen’s study, UCSC Xena (http://xena.ucsc.edu/), GDC repository (https://portal.gdc.cancer.gov/), and TISCH2 (http://tisch.comp-genomics.org/home/). In this study, the bulk RNA-seq level data sets used from the GEO database are respectively: BLCA-GSE236932 (62), BRCA-GSE233242 (63), ESCA-GSE130078 (64), HNSC-GSE178537 (65), KIRC-GSE126964 (66), KIRP-GSE180777 (67), LIHC-GSE214846 (68), LUAD-GSE233774 (69), PRAD-GSE237995 (70), THCA-GSE83520 (71), and UCEC-GSE146889 (72). The single-cell level data set used is LUAD-GSE123902 (73).

## SUPPLEMENTAL MATERIAL

Supplemental figures, part 1 msystems.01146-25-s0001.docx Fig. S1 to S7.

Supplemental figures, part 2 msystems.01146-25-s0002.docx Fig. S8 to S22.

Supplemental figures, part 3 msystems.01146-25-s0003.docx Fig. S23 to S32.

Supplemental text msystems.01146-25-s0004.docx Detailed additional methodology.

Supplemental Tables msystems.01146-25-s0005.xlsx Tables S1 to S17.

ASM does not own the copyrights to Supplemental Material that may be linked to, or accessed through, an article. The authors have granted ASM a non-exclusive, world-wide license to publish the Supplemental Material files. Please contact the corresponding author directly for reuse.
