# Supplementary material for: Effect of immune-related intratumoral microbiota and host gene expression on cancer prognosis
Source: mSystems. 2025 Sep 15;10(10):e01146-25. doi: 10.1128/msystems.01146-25 (PMC12542631; doi:10.1128/msystems.01146-25)
Supplement: Supplemental figures, part 1 — Fig. S1 to S7. [file msystems.01146-25-s0001.docx]

**Supplementary Figure 1-7**


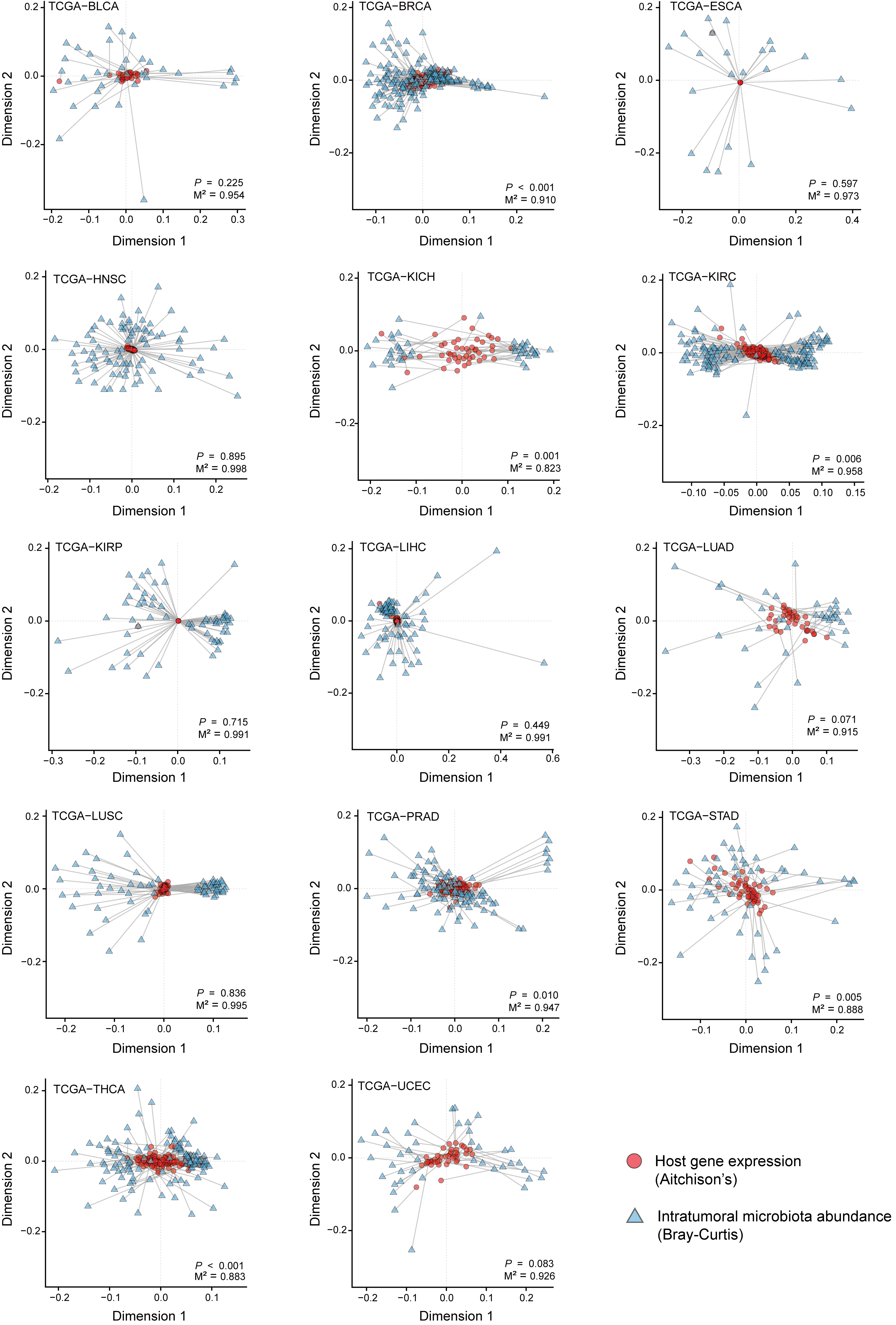


**Supplementary Figure 1 The overall correlations between host gene expression and intratumoral microbiota in 14 TCGA-tumors.**

The overall correlations between host gene expression and intratumoral microbiota abundance across 14 tumors was investigated using Procrustes analysis. Host gene expression data (circles) were represented by Aitchison distance, while intratumoral microbiota data (triangles) were represented by Bray-Curtis distance.


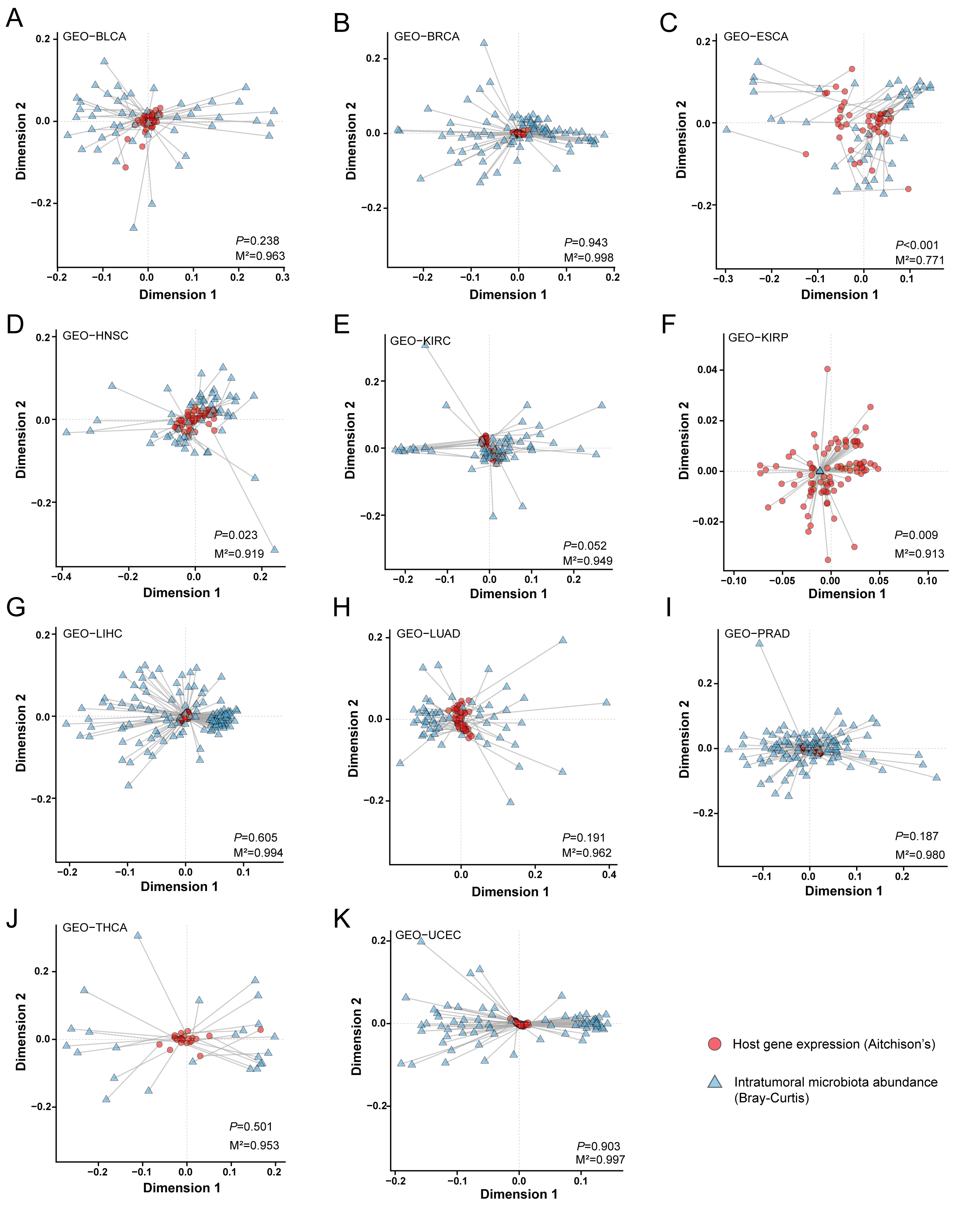


**Supplementary Figure 2 The overall correlations between host gene expression and intratumoral microbiota in 11 GEO-tumors.**

**A-K** The overall correlations between host gene expression and intratumoral microbiota abundance across 11 GEO-tumors was investigated using Procrustes analysis. Host gene expression data (circles) were represented by Aitchison distance, while intratumoral microbiota data (triangles) were represented by Bray-Curtis distance.


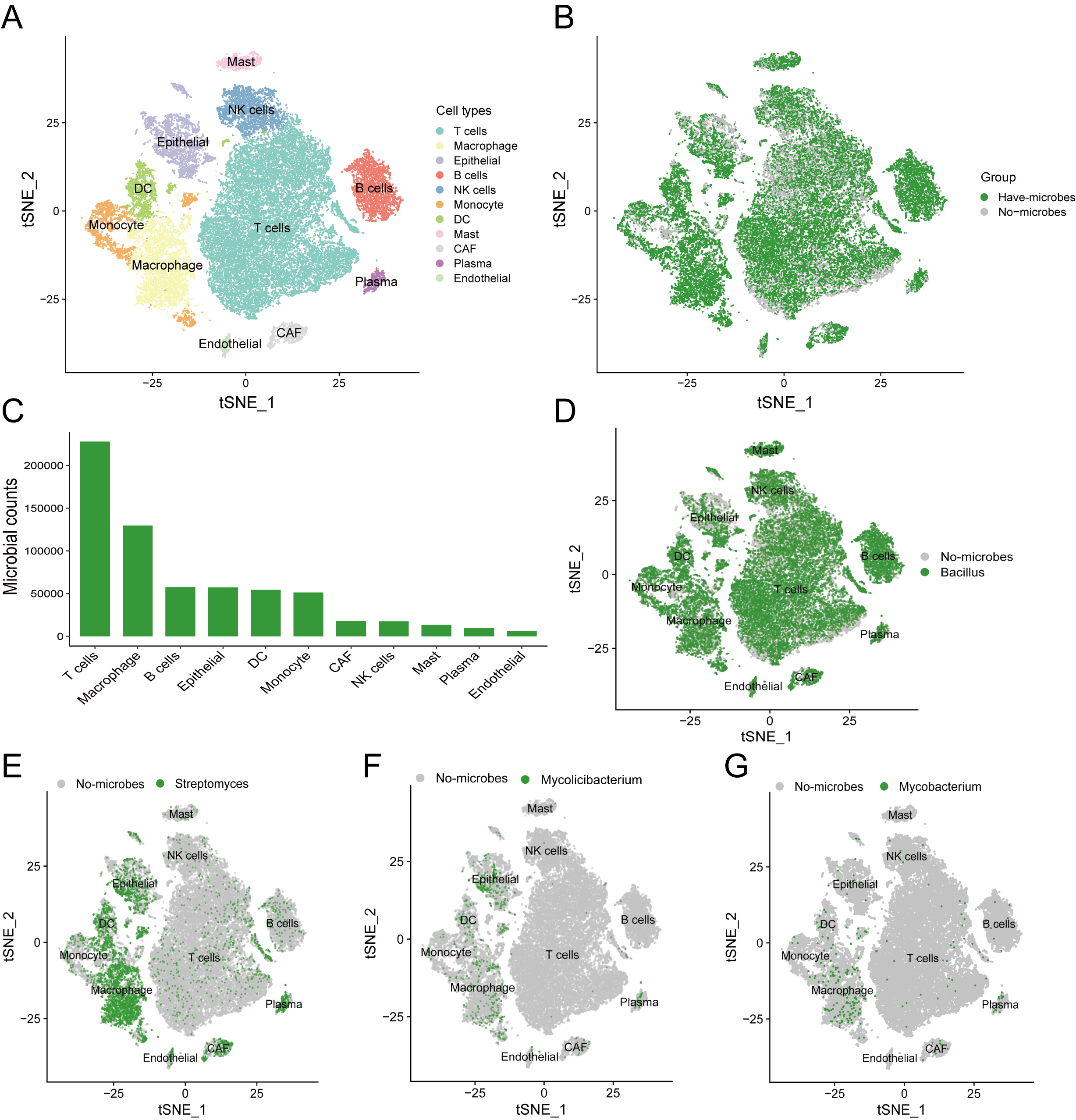


**Supplementary Figure 3 Identification of microbes within LUAD tumors at the single-cell level and their association with the gene expression of various host cell subtypes.**

A-B: The annotation of individual cell subtypes and the mapping of intratumoral microbiota at the single-cell level in LUAD. C: The count of microbes co-localized with each cell subtype. D-G: The distribution of *Bacillus*, *Streptomyces*, *Mycolicbacterium* and *Mycobacterium* in each cell subtype. The green dots represent cells co-localized with microbes, and the gray dots represent cells not co-localized with microbes.


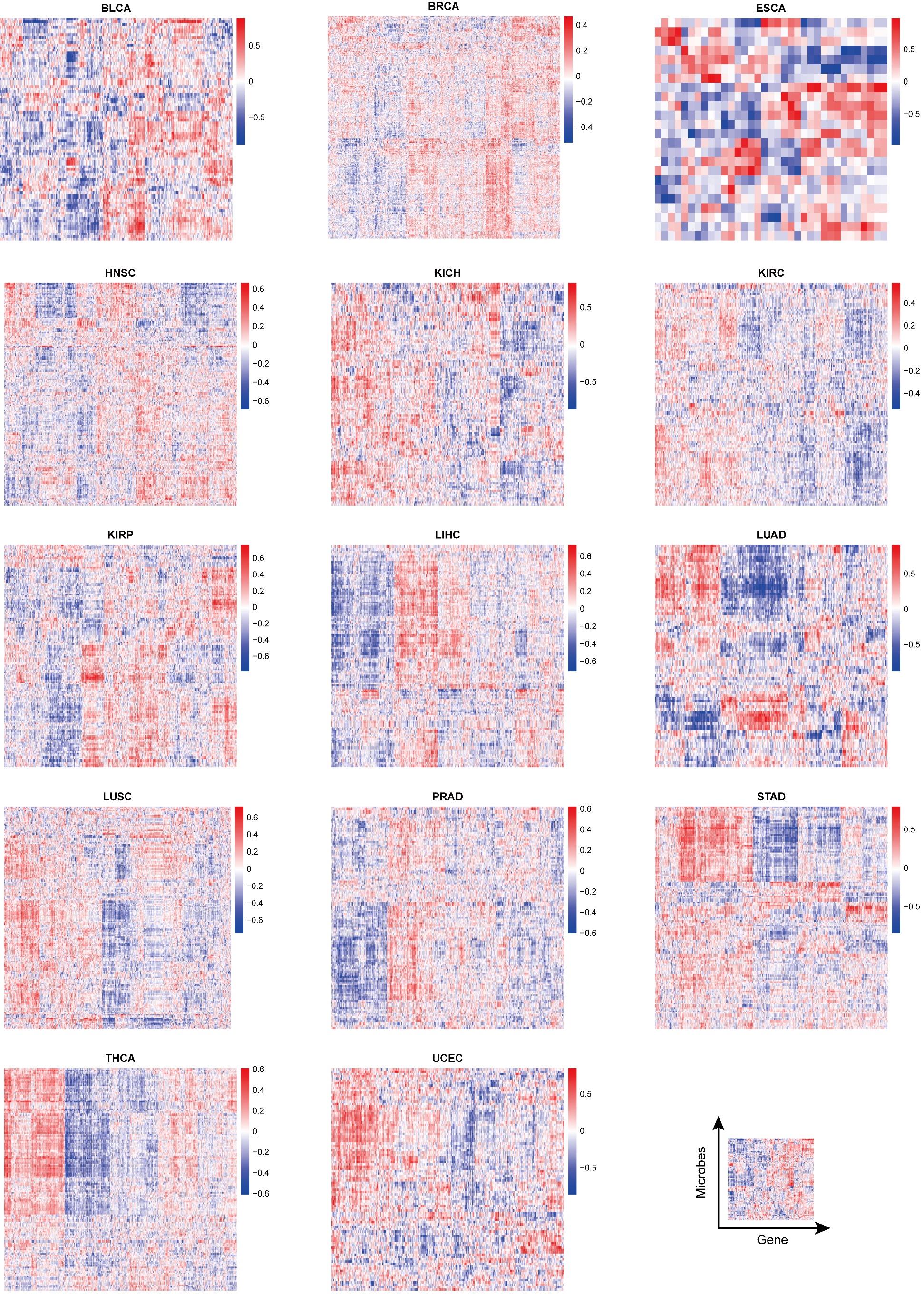


**Supplementary Figure 4 Correlation of host genes and intratumoral microbes in each tumor identified by Lasso regression.**

The heatmap shows the correlations between host genes (rows) and intratumoral microbes (columns), identified through Lasso and stable selection for specific microbe-host gene associations with FDR < 0.1, across 14 TCGA tumors.

**
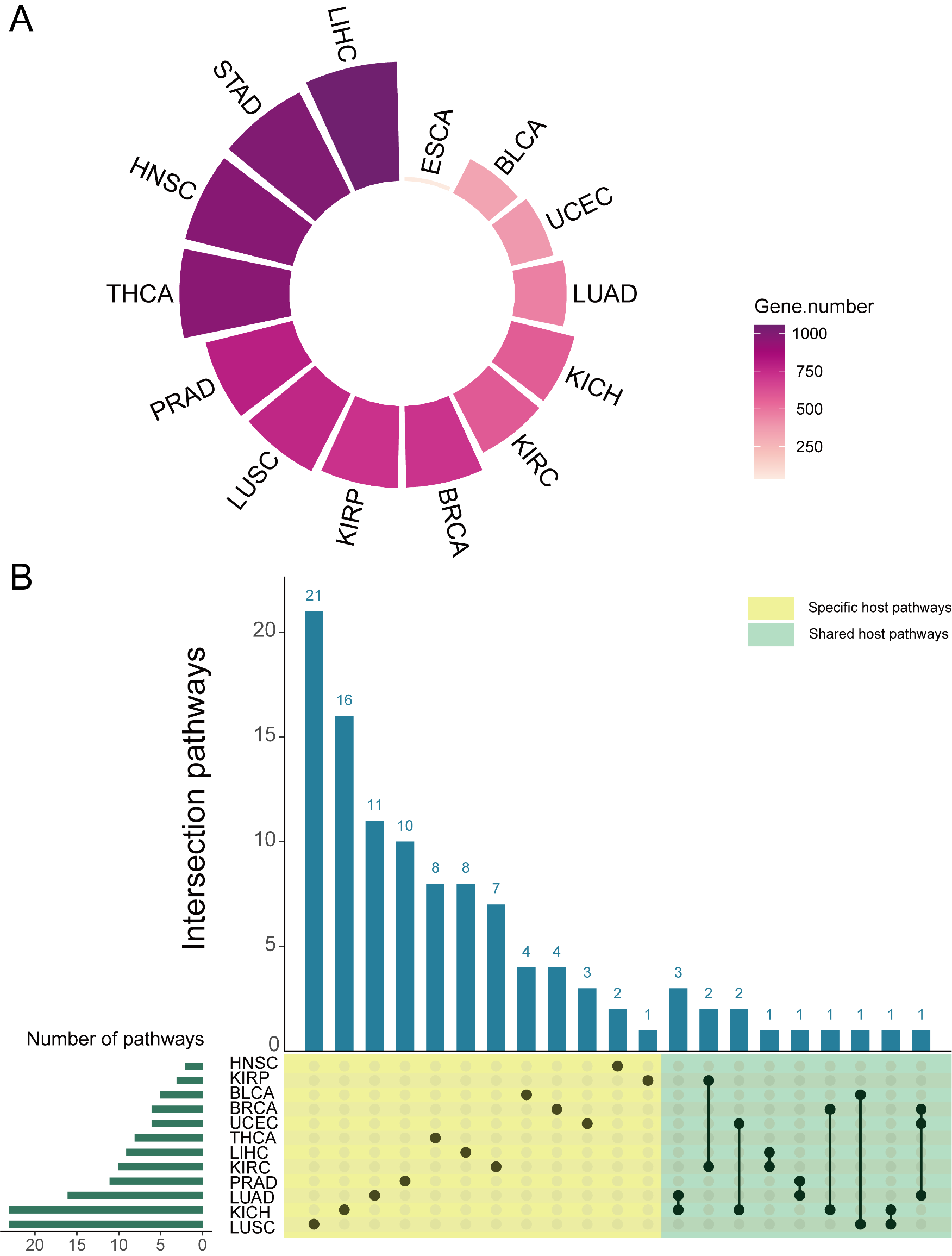
**

**Supplementary Figure 5 Specific intratumoral microbes associated with individual host genes and pathways in each tumor.**

**A** The number of host genes associated with specific intratumoral microbes across 14 tumors. **B** The host pathways enriched by host genes related to specific intratumoral microbes in 12 tumors (FDR < 0.1). The bar plot on the left illustrates the number of pathways enriched by genes associated with specific microbes in each tumor. The bar plot on top shows the number of overlapping pathways. The black dots at the bottom represent the number of tumor-specific pathways enriched in each tumor (yellow fill) and the number of shared pathways among tumors (green fill).


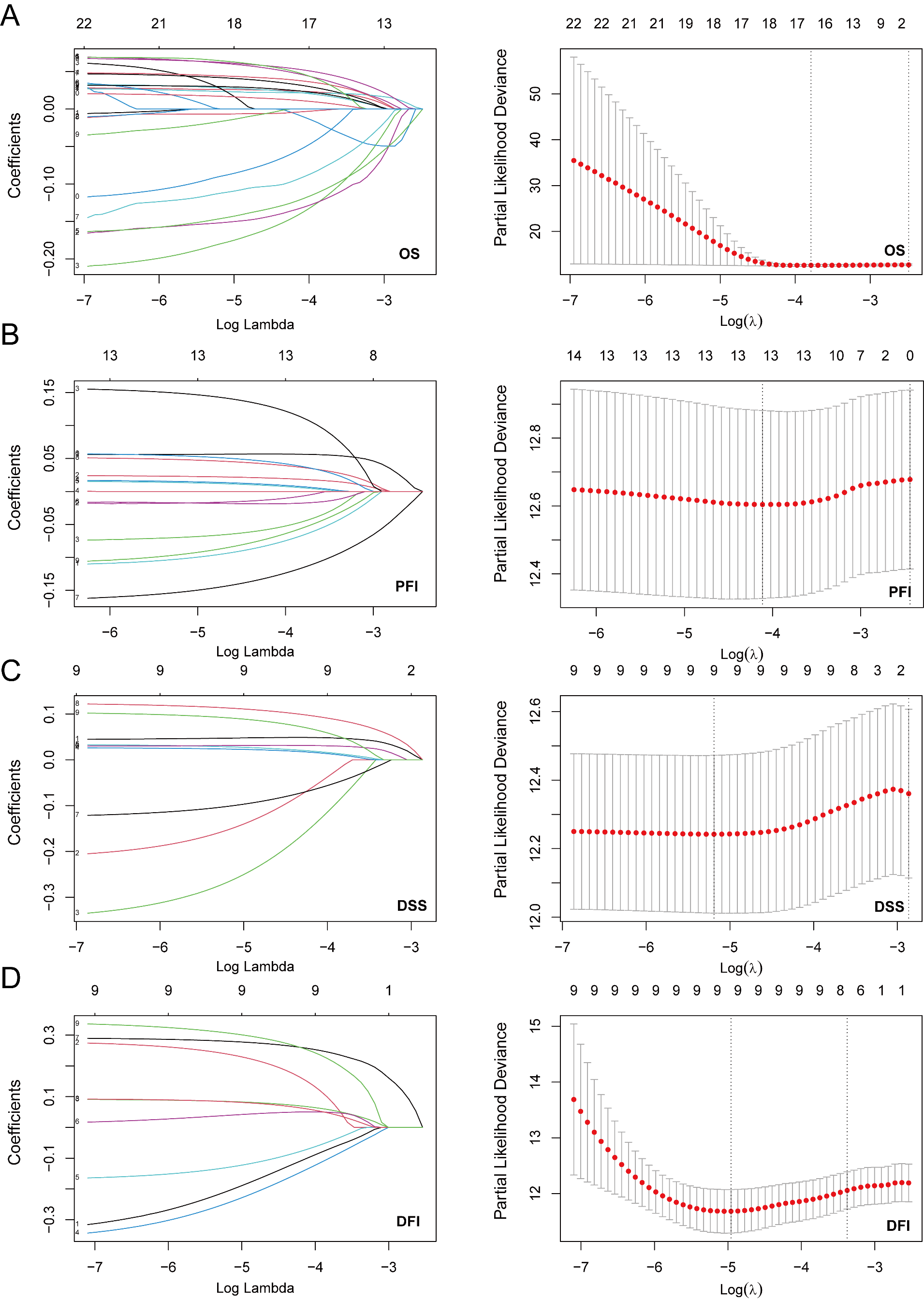


**Supplementary Figure 6 Screening of microbial features related to OS, PFI, DSS, and DFI and construction of the IPRMSs in TCGA-paired cohort.**

**A** Selected 17 microbes related to OS based on Lasso Cox regression. **B** Selected 13 microbes related to PFI with Lasso Cox. **C** Selected 9 microbes related to DSS with Lasso Cox. **D** Select 9 microbes related to DFI with Lasso Cox. Note: DFI-IPRMS was calculated with *P*<0.2 for *P*-threshold of microbial differential analysis in order to obtain more microbes associated with immune and prognosis.


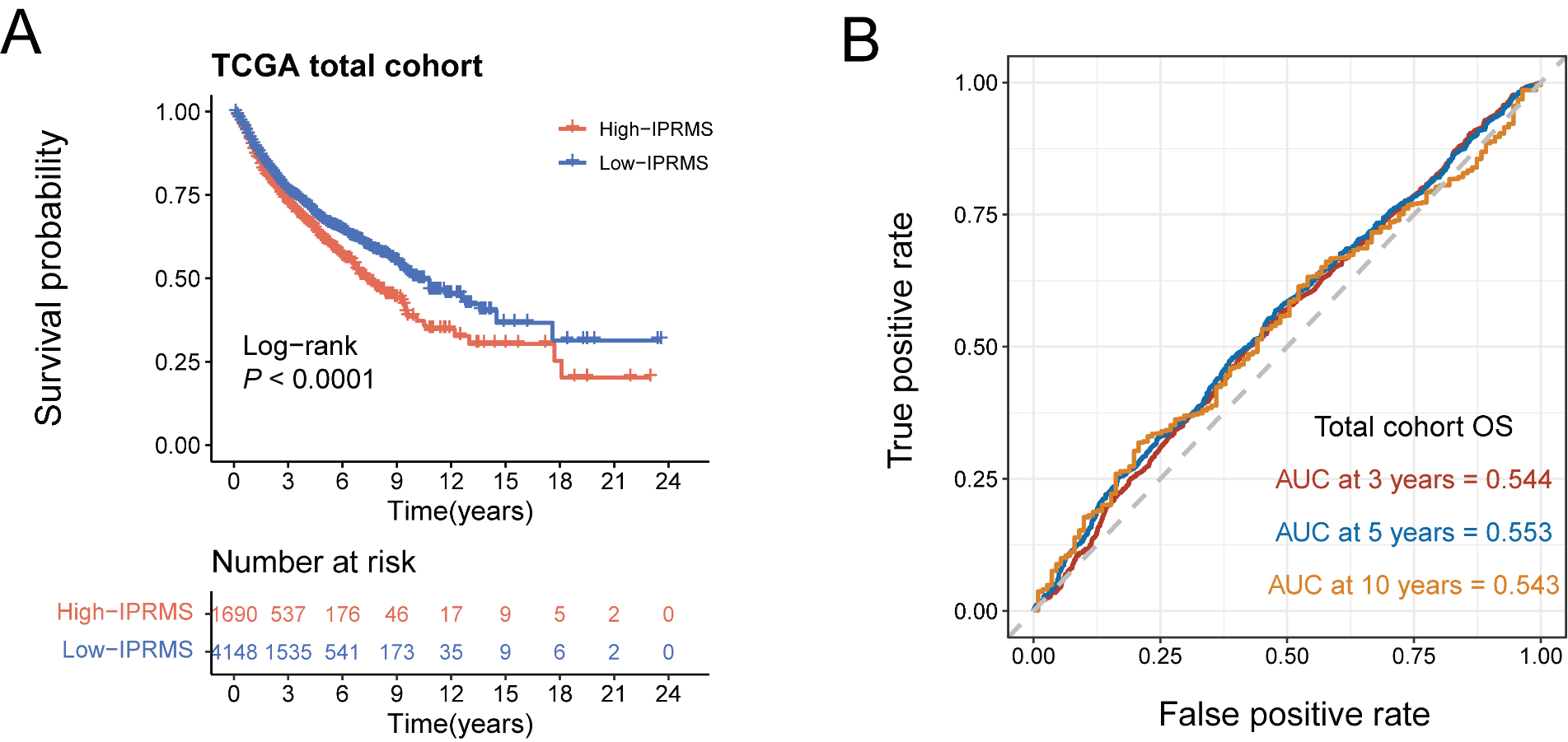


**Supplementary Figure 7 The associations of IPRMS with OS in** **TCGA-total cohort.**

**A** Kaplan Meier curves of OS for TCGA-total cohort based on OS-IPRMS classification. **B** ROC curves of OS for TCGA-total cohort based on OS-IPRMS classification.
