# Supplementary material for: Effect of immune-related intratumoral microbiota and host gene expression on cancer prognosis
Source: mSystems. 2025 Sep 15;10(10):e01146-25. doi: 10.1128/msystems.01146-25 (PMC12542631; doi:10.1128/msystems.01146-25)
Supplement: Supplemental figures, part 2 — Fig. S8 to S22. [file msystems.01146-25-s0002.docx]

**Supplementary Figure 8-22**


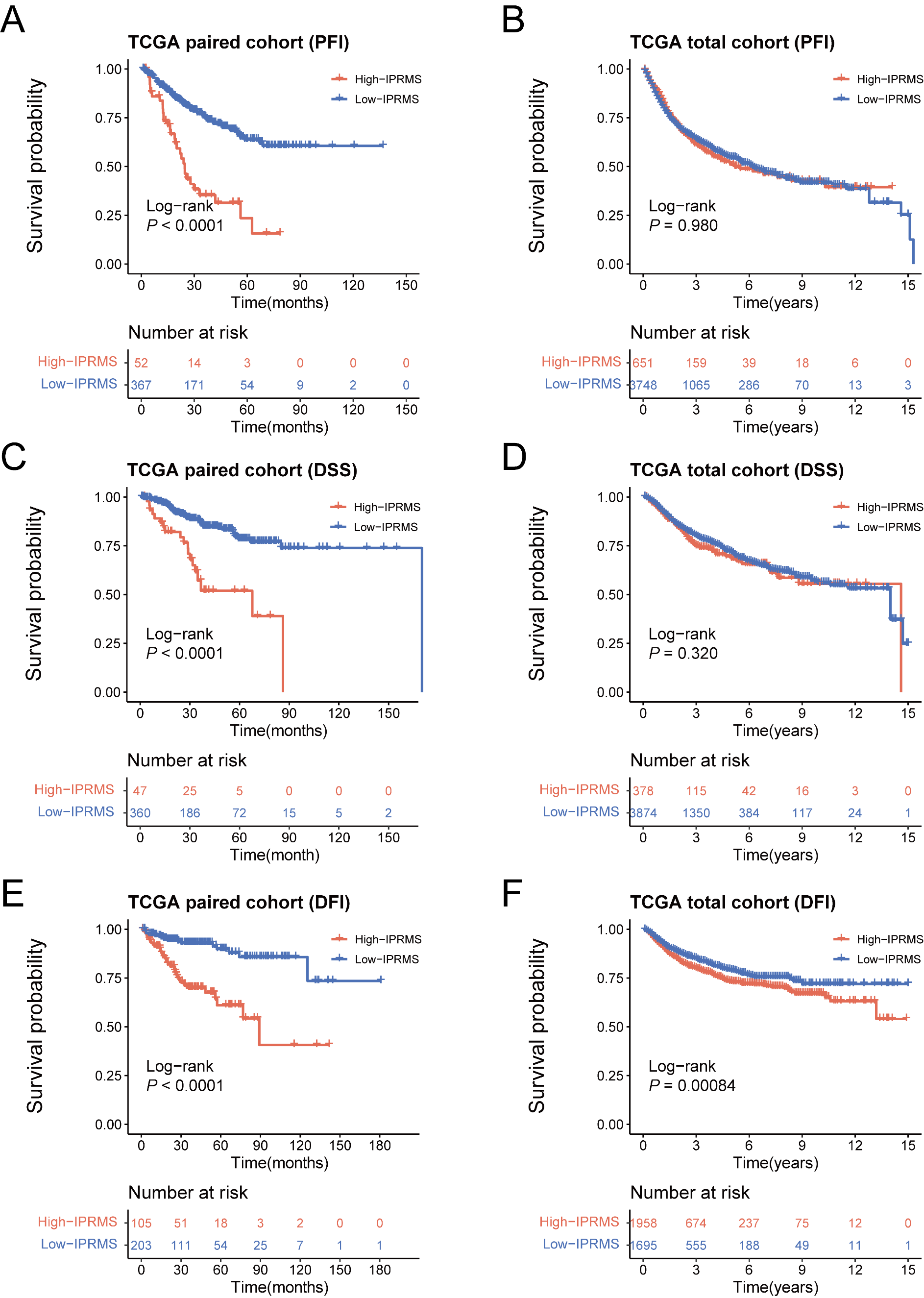


**Supplementary Figure 8 The associations of IPRMS with PFI, DSS and DFI.**

**A-B** Kaplan Meier curves of PFI for TCGA-paired cohort (A) and TCGA-total cohort (B) based on PFI-IPRMS classification. **C-D** Kaplan Meier curves of DSS for TCGA-paired cohort (C) and TCGA-total cohort (D) based on DSS-IPRMS classification. **E-F** Kaplan Meier curves of DFI for TCGA-paired cohort (E) and TCGA-total cohort (F) based on DFI-IPRMS classification.


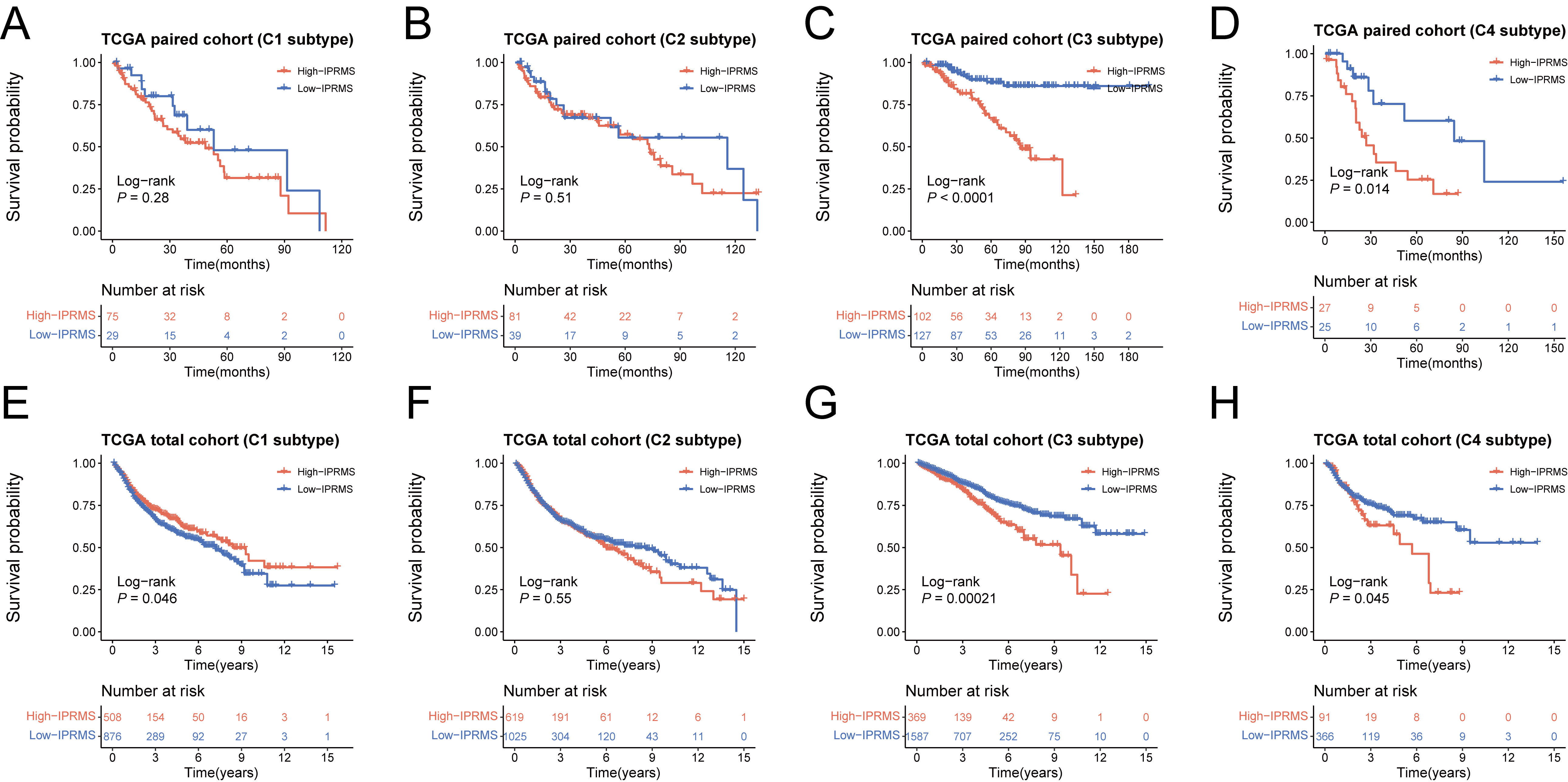


**Supplementary Figure 9 The association between IPRMS and OS in four different immune subtypes**

**A-D** Kaplan-Meier curves of C1 (wound healing) immune subtype, C2 (IFN-γ dominant) immune subtype, C3 (inflammatory) immune subtype, and C4 (lymphocyte depleted) immune subtype in TCGA-paired cohort stratified by IPRMS classification. **E-H** Kaplan-Meier curves of C1 immune subtype, C2 immune subtype, C3 immune subtype, and C4 immune subtype in TCGA-total cohort stratified by IPRMS classification.


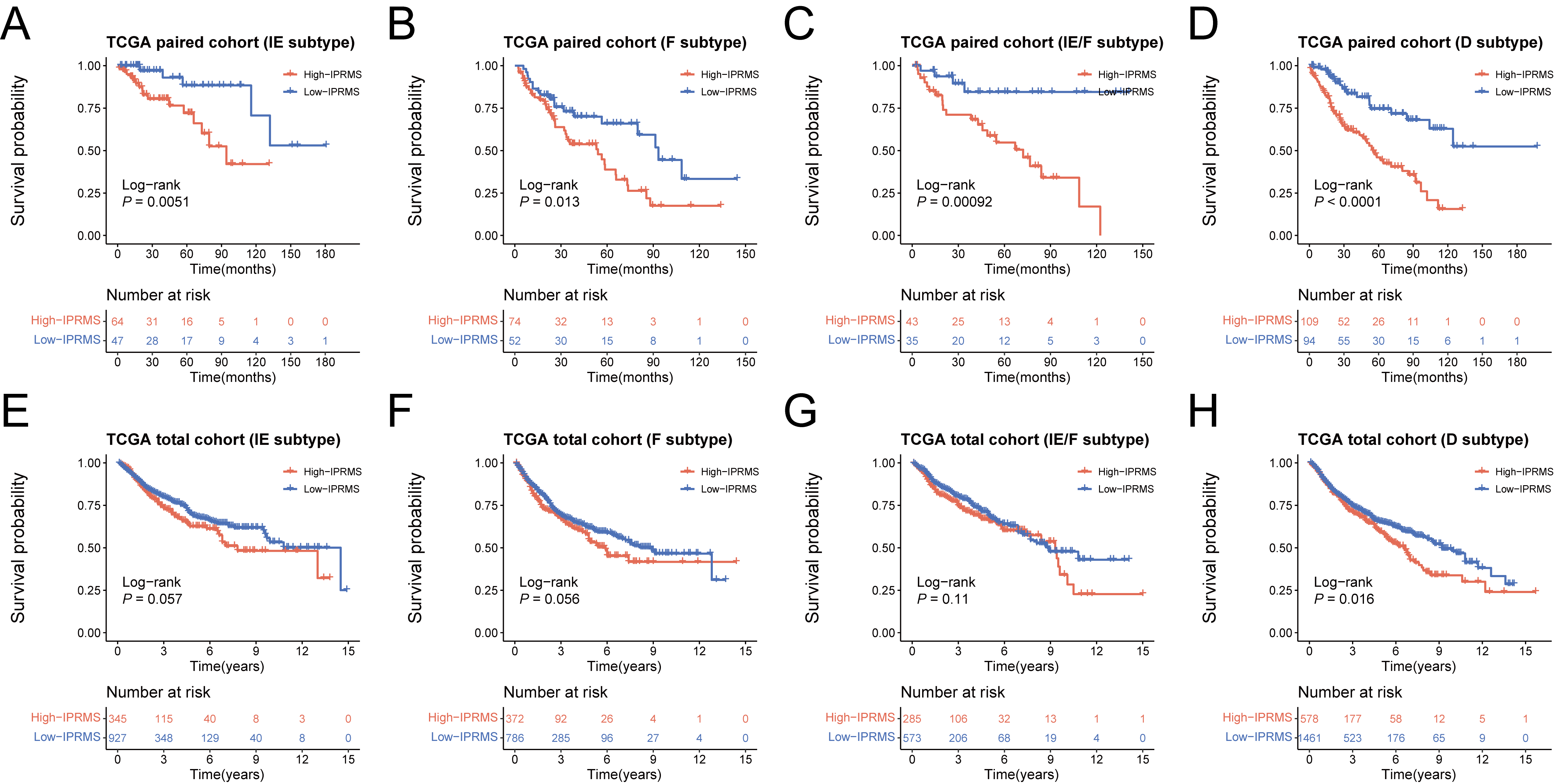


**Supplementary Figure 10 The association between IPRMS and OS in four different tumor microenvironment (TME) subtypes.**

**A-D** Kaplan-Meier curves of IE (Immune-Enriches, Non-Fibrotic tumor) TME subtype, F (Fibrotic tumor) TME subtype, IE/F (Immune-Enriches, Fibrotic tumor) TME subtype, and D (Depleted tumor) TME subtype in TCGA-paired cohort stratified by IPRMS classification. **E-H** Kaplan-Meier curves of IE TME subtype, F TME subtype, IE/F TME subtype, and D TME subtype in TCGA-total cohort stratified by IPRMS classification.


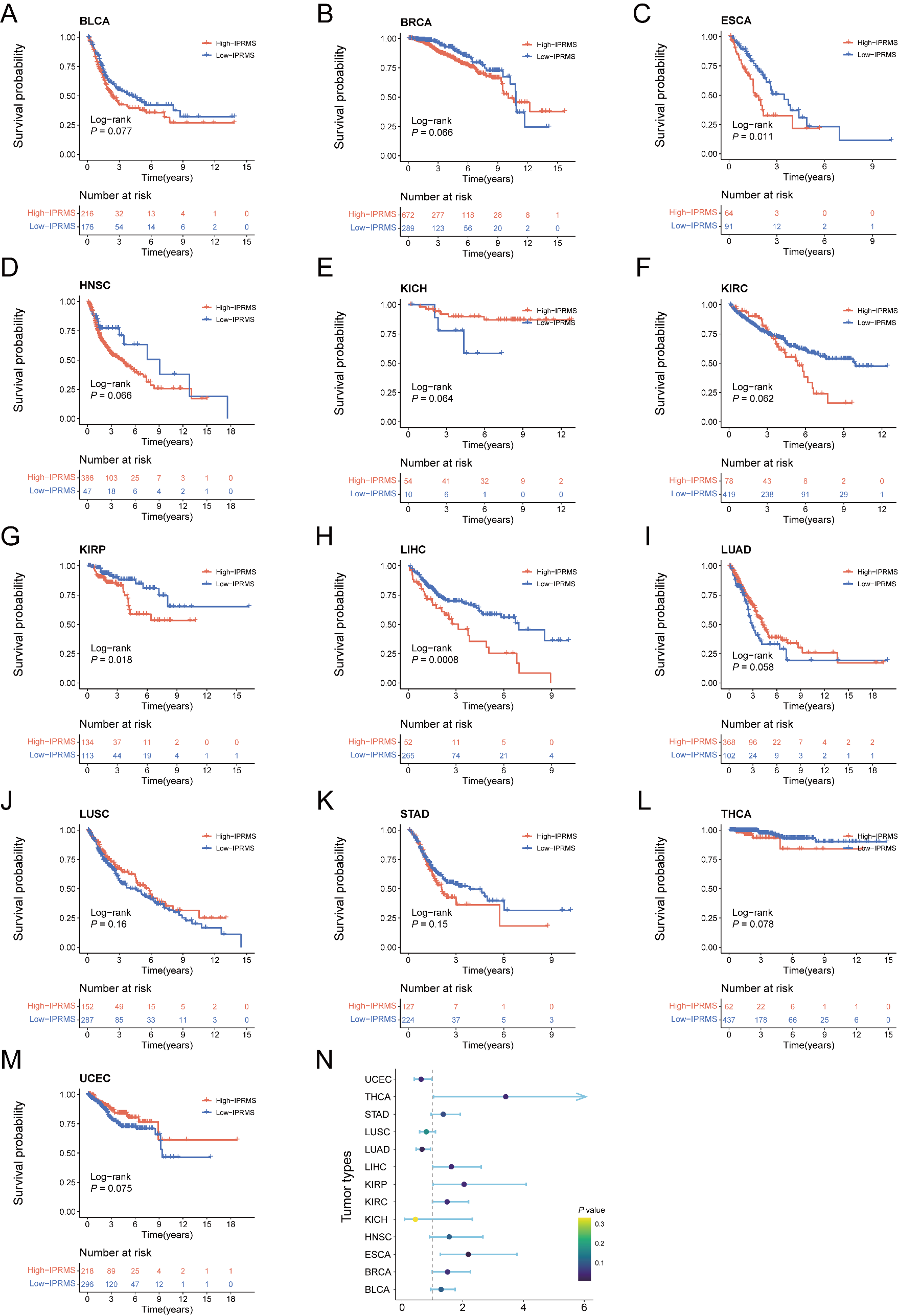


**Supplementary Figure 11 The association between pan-cancer IPRMS and the prognosis in each tumor.**

**A-M** Kaplan-Meier curves of 13 tumors in TCGA-total cohort stratified by pan-cancer IPRMS classification. The 13 tumors included BLCA, BRCA, ESCA, HNSC, KICH, KIRC, KIRP, LIHC, LUAD, LUSC, STAD, THCA, and UCEC. **N** The forest plot illustrates the association between pan-cancer IPRMS and the prognosis of 13 different tumors based on multivariate Cox. The factors adjusted for in the analysis were age, gender, race, and tumor stage, while for BRCA and UCEC, adjustments were made for age, race, and tumor stage.


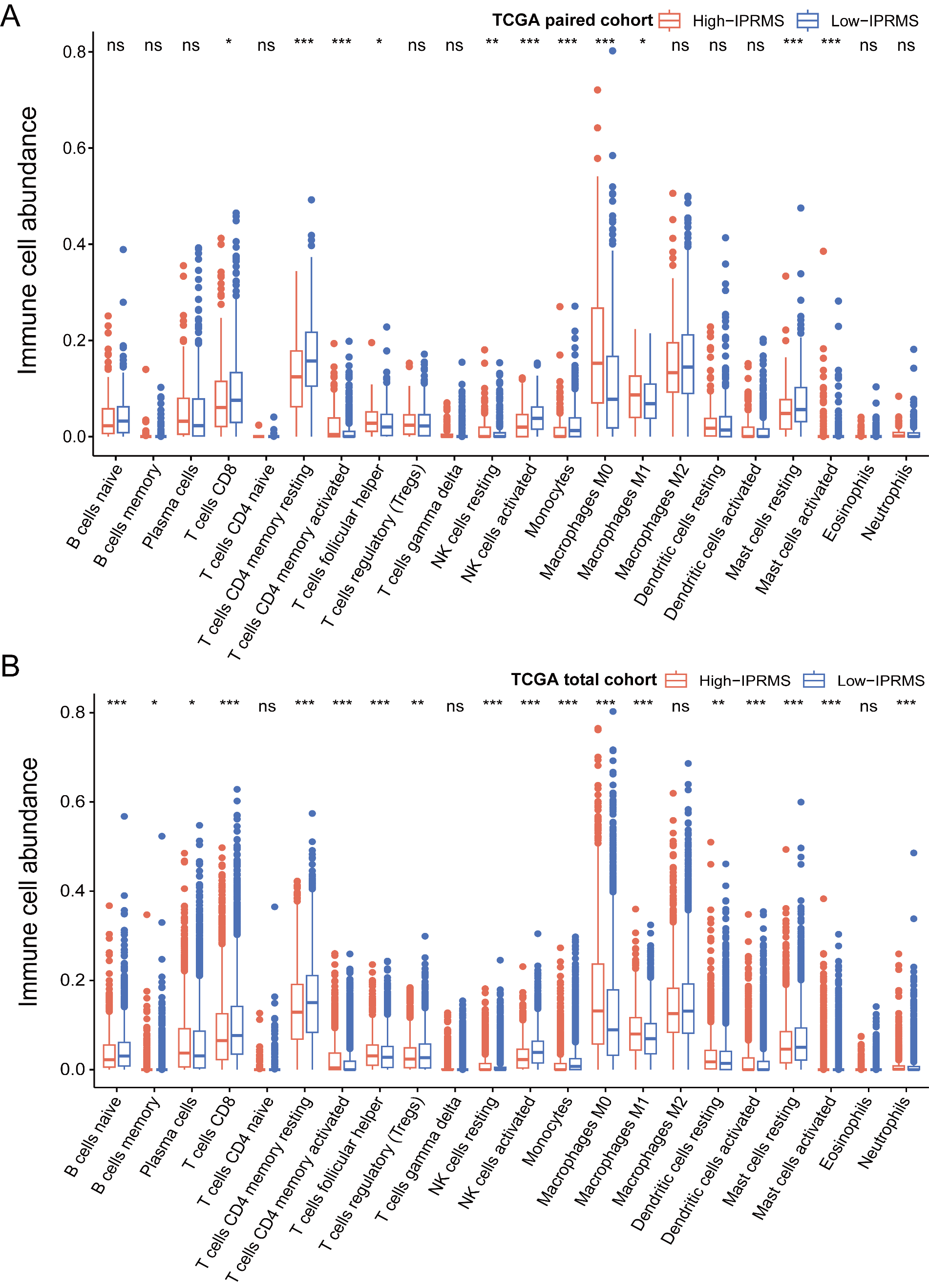


**Supplementary Figure 12 The immune infiltration situation based on CIBERSORT algorithm in TCGA-paired cohort and TCGA-total cohort.**

The differences in the abundance of infiltrating immune cells between the Low-IPRMS group and the High-IPRMS group in the (A) TCGA-paired cohort and (B) TCGA-total cohort were analyzed using the CIBERSORT algorithm. * *P*<0.05; ** *P*<0.01; *** *P*<0.001


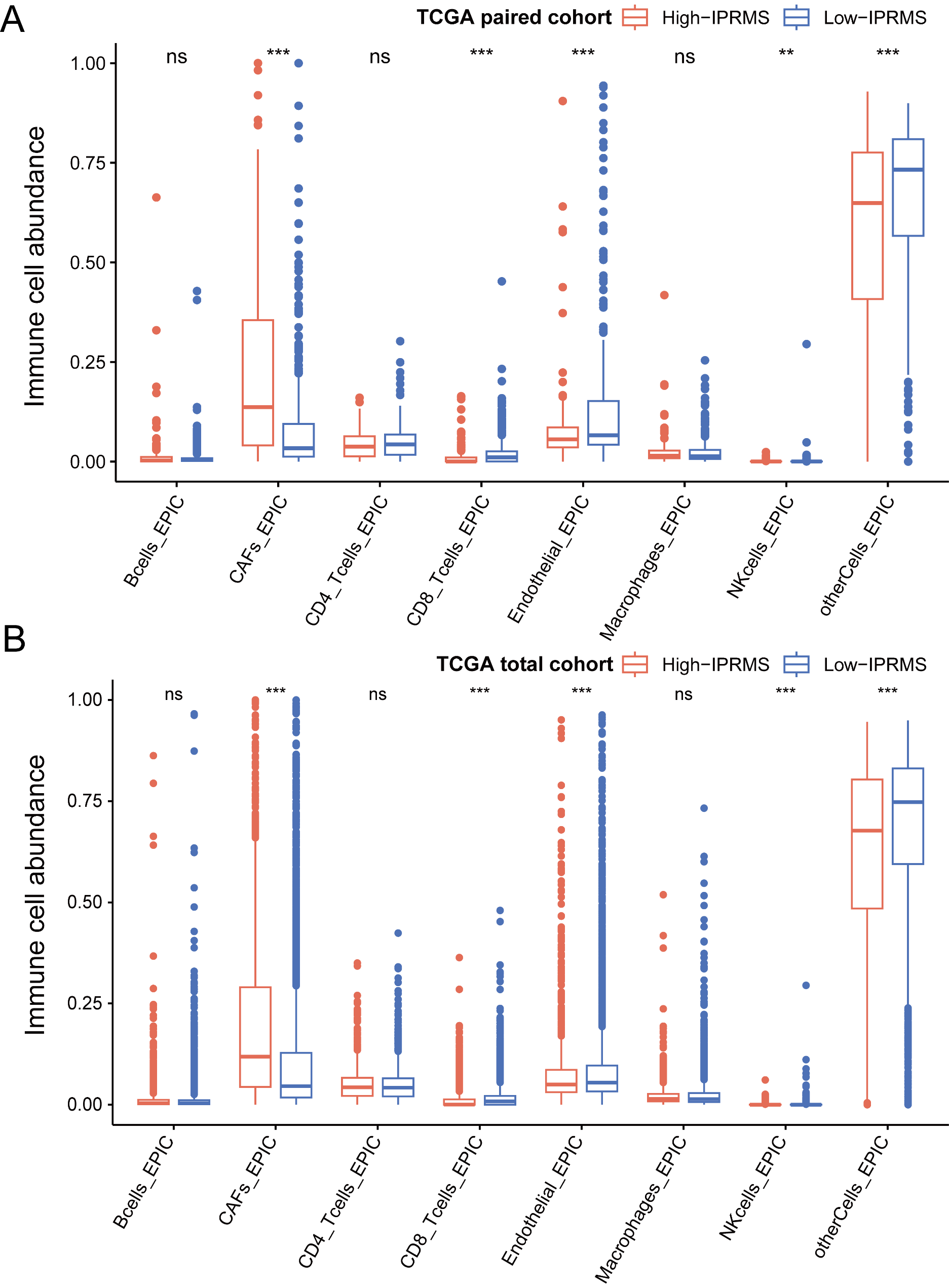


**Supplementary Figure 13 The immune infiltration situation based on EPIC algorithms in TCGA-total cohort.**

The differences in the abundance of infiltrating immune cells between the Low-IPRMS group and the High-IPRMS group in the (A) TCGA-paired cohort and (B) TCGA-total cohort were analyzed using the EPIC algorithm. * *P*<0.05; ** *P*<0.01; *** *P*<0.001


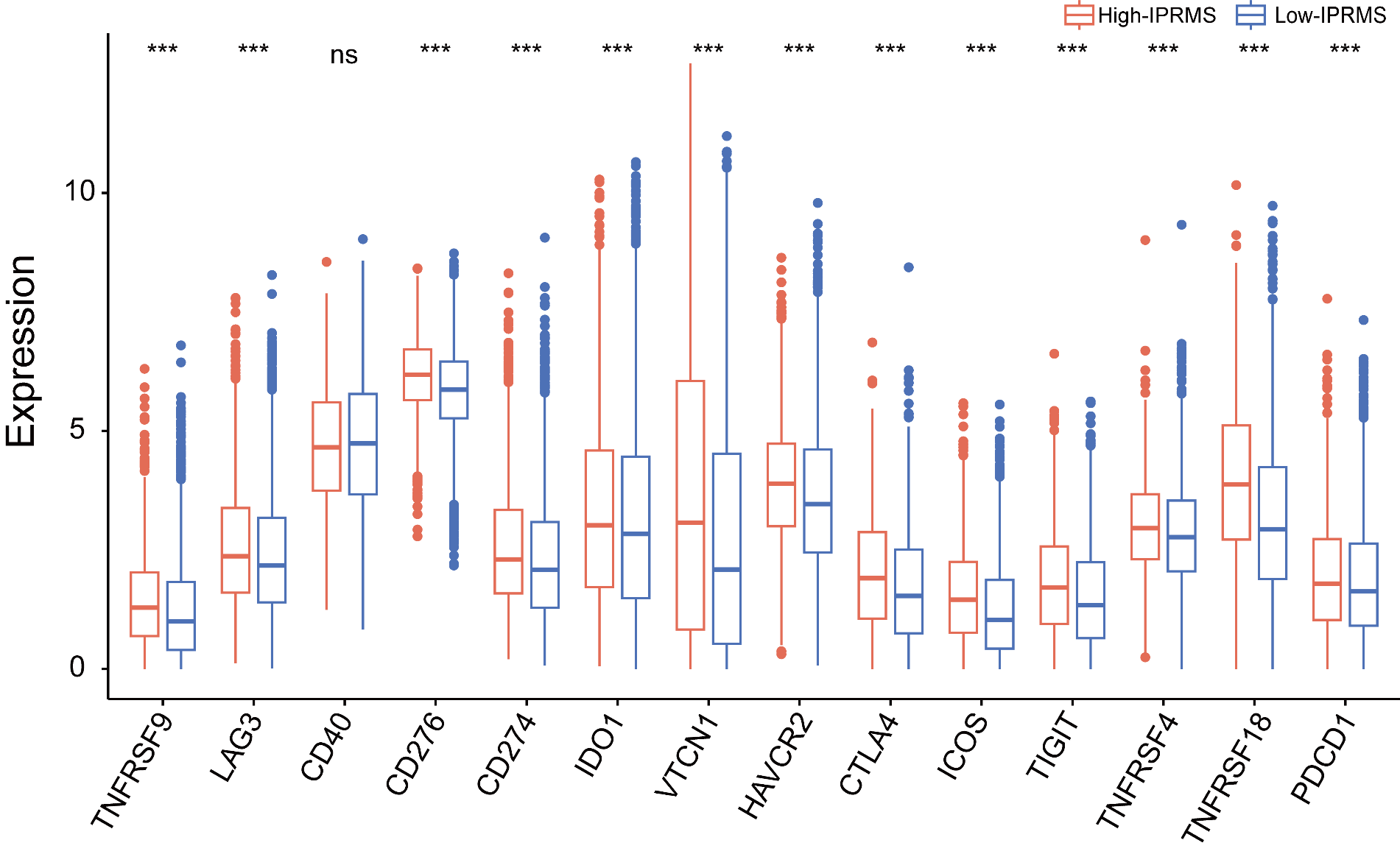


**Supplementary Figure 14 The differences of ICP genes expression** **between different IPRMS groups in TCGA-total cohort.**

The differences of ICP genes expression between the Low-IPRMS group and the High-IPRMS group in the TCGA-total. * *P*<0.05; ** *P*<0.01; *** *P*<0.001


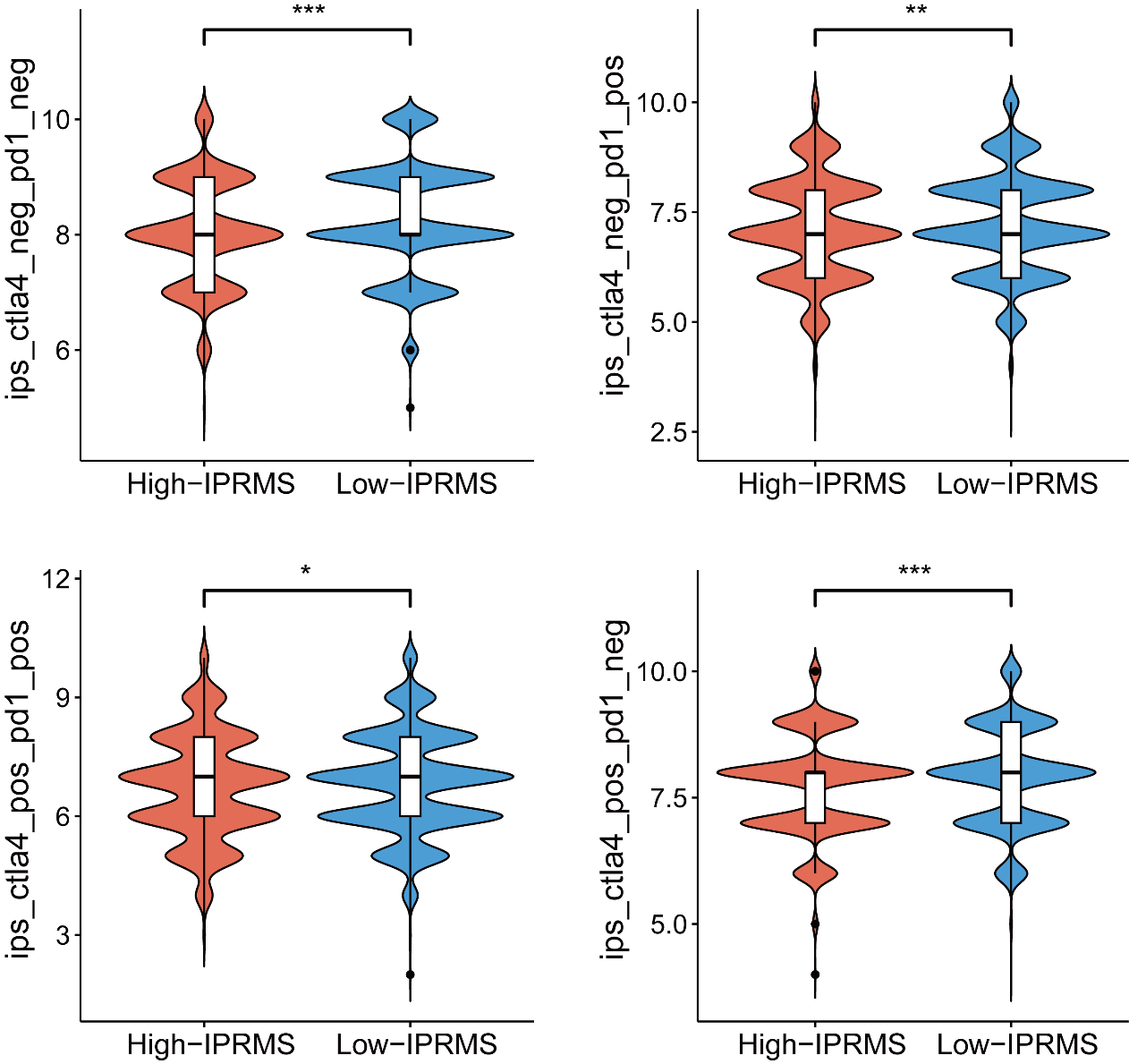


**Supplementary Figure 15 The difference in IPS between High-IPRMS and Low-IPRMS group in TCGA-total cohort.**

Distribution of IPS among four IPS types (CTLA4- PD1-, CTLA4- PD1+, CTLA4+ PD1+, CTLA4+ PD1-) between High-IPRMS and Low-IPRMS group in TCGA-total cohort. * *P*<0.05; ** *P*<0.01; *** *P*<0.001


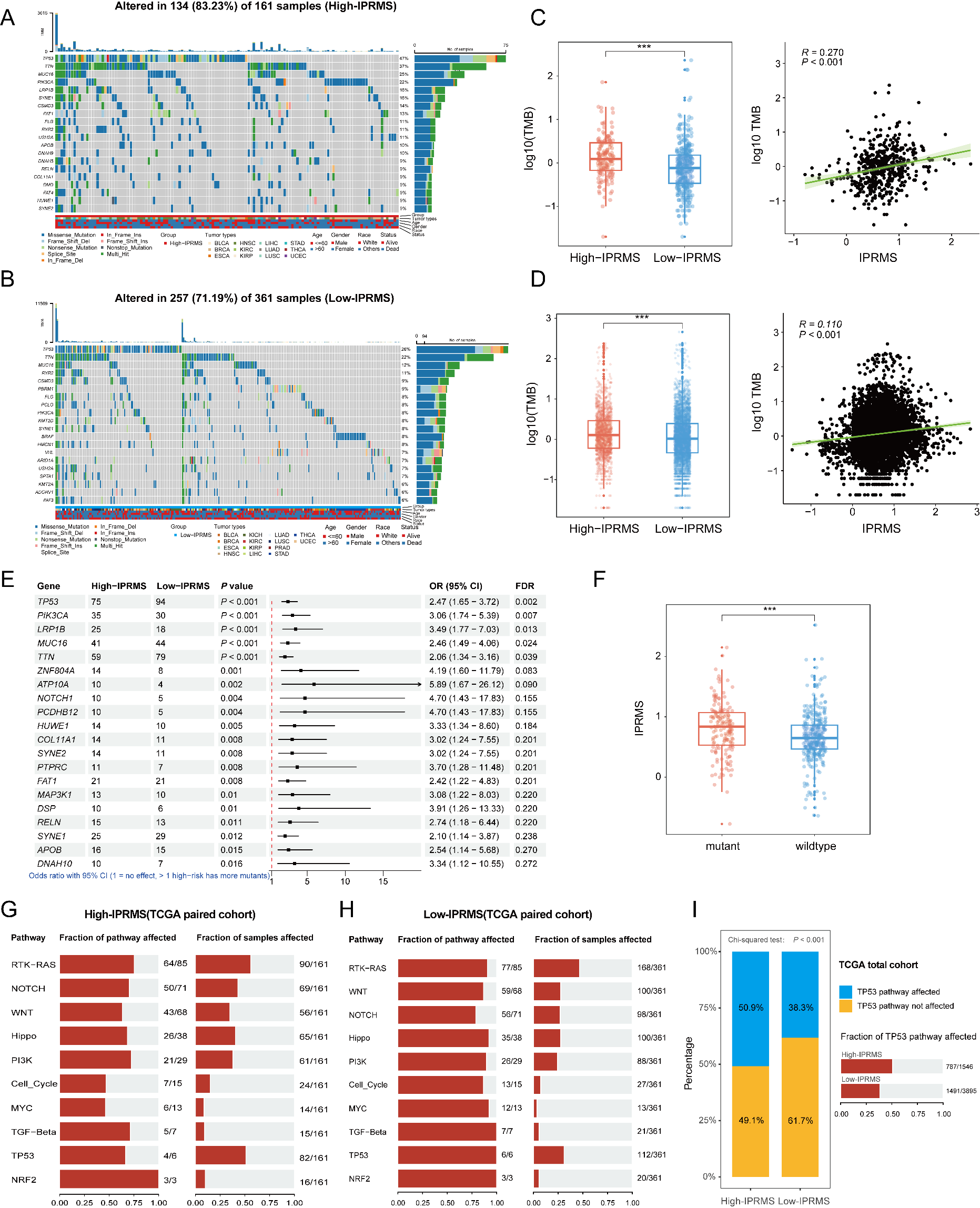


**Supplementary Figure 16 The association between IPRMS and host genomic variation.**

**A-B** The mutational landscape of significantly mutated genes in TCGA-paired cohort stratified by High-IPRMS group (A) versus Low-IPRMS group (B). **C** The difference of TMB between the two IPRMS groups (left plot) and the correlation between IPRMS and TMB (right plot) in TCGA-paired cohort. **D** The difference of TMB between the two IPRMS groups (left plot) and the correlation between IPRMS and TMB (right plot) in TCGA-total cohort. **E** The forest plot shows the associations between IPRMS groups and top 20 gene with the highest mutation frequency. **F** The difference of IPRMS between TP53 mutant type and wild type in TCGA-paired cohort. **G-H** Canonical driver pathway mutations, especially the TP53 signaling pathway mutations, in High-IPRMS group and Low-IPRMS group in TCGA-paired cohort. **I** Difference in the proportion of patients affected by TP53 pathway mutations between the High-IPRMS group and the Low-IPRMS group in the TCGA total cohort. **P*<0.05; ***P*<0.01; ****P*<0.001


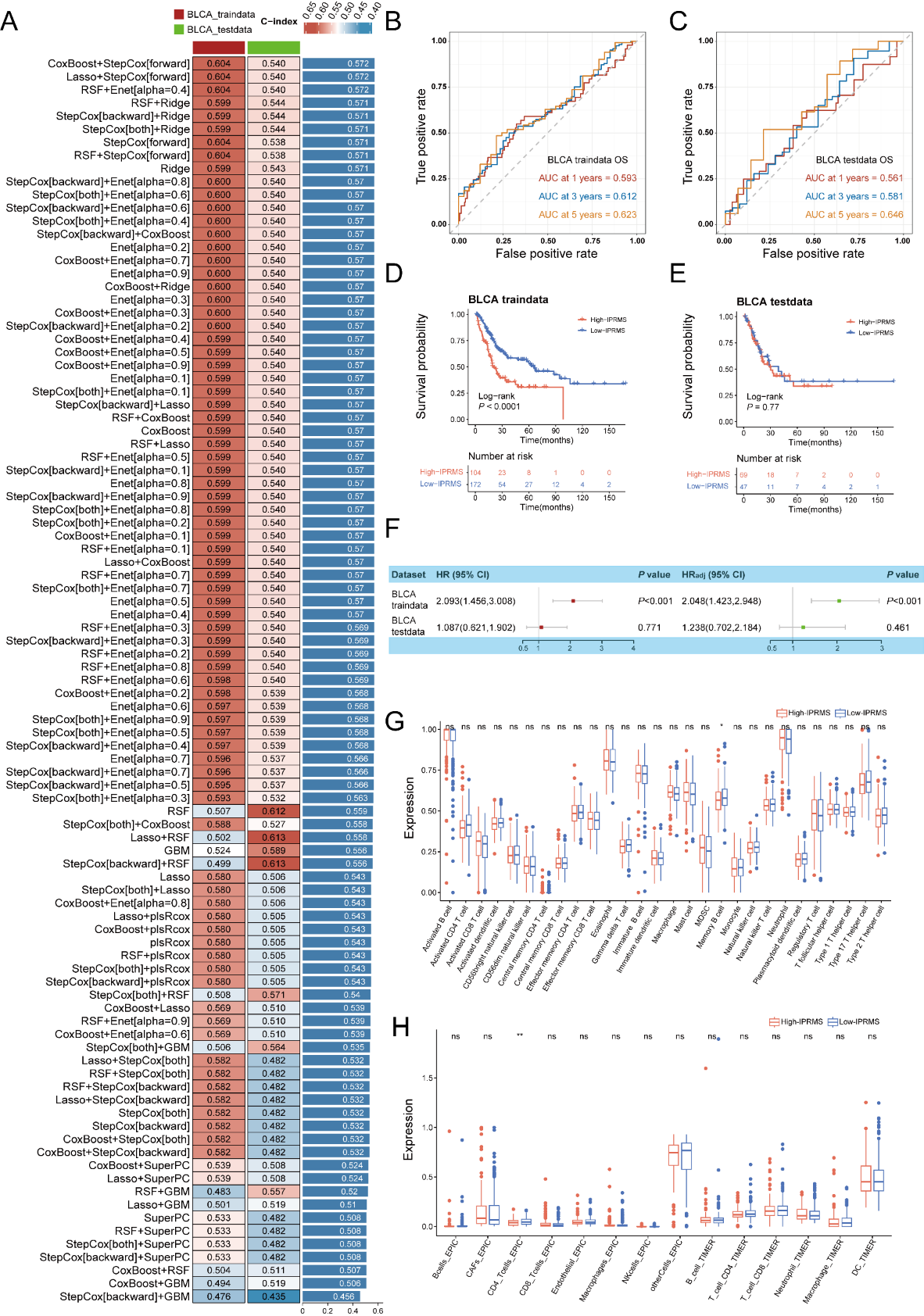


**Supplementary Figure 17 Construction of BLCA-IPRMS and its association with prognosis and immune infiltration.**

**A**. The C-index for traindata, testdata, and average C-index of each ML combination in BLCA. **B-C** ROC curve of the prognostic model based on IPRMS for BLCA traindata (B) and BLCA testdata (C). **D-E** Associations between BLCA-IPRMS and survival in BLCA traindata (D) and BLCA testdata (E). **F** Univariate and multivariate associations between IPRMS and OS, adjust factors were age, gender, race, and tumor stage. **G** The difference of infiltrating estimations of 28 immune cells in TME between High-IPRMS and Low-IPRMS groups. **H** The difference of abundance of immune cells between High-IPRMS and Low-IPRMS groups based on the EPIC and TIMER. * *P*<0.05; ** *P*<0.01; *** *P*<0.001


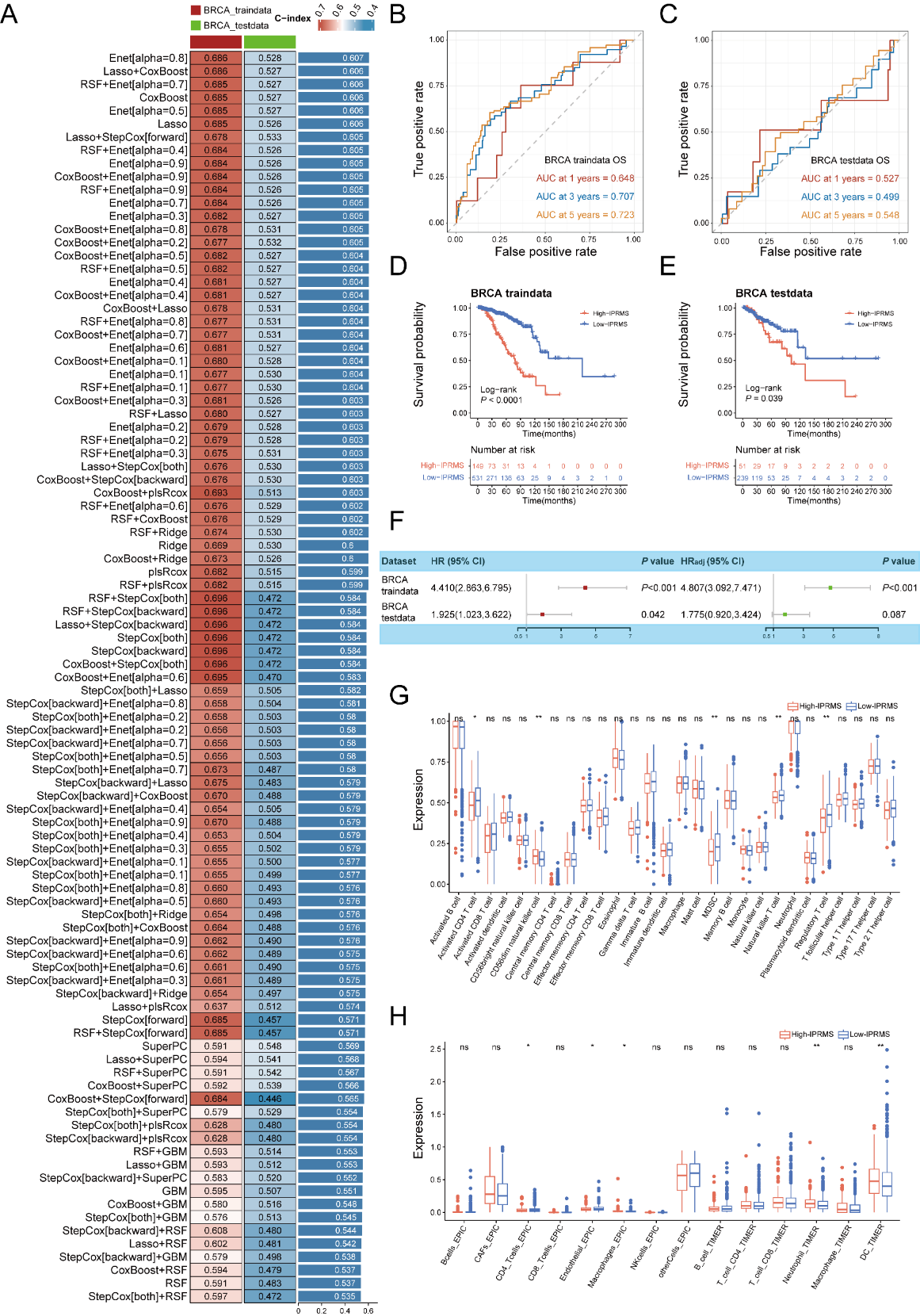


**Supplementary Figure 18 Construction of BRCA-IPRMS and its association with prognosis and** **immune infiltration.**

**A**. The C-index for traindata, testdata, and average C-index of each ML combination in BRCA. **B-C** ROC curve of the prognostic model based on IPRMS for BRCA traindata (B) and BRCA testdata (C). **D-E** Associations between BRCA-IPRMS and survival in BRCA traindata (D) and BRCA testdata (E). **F** Univariate and multivariate associations between IPRMS and OS, adjust factors were age, race, and tumor stage. **G** The difference of infiltrating estimations of 28 immune cells in TME between High-IPRMS and Low-IPRMS groups. **H** The difference of abundance of immune cells between High-IPRMS and Low-IPRMS groups based on the EPIC and TIMER. * *P*<0.05; ** *P*<0.01; *** *P*<0.001


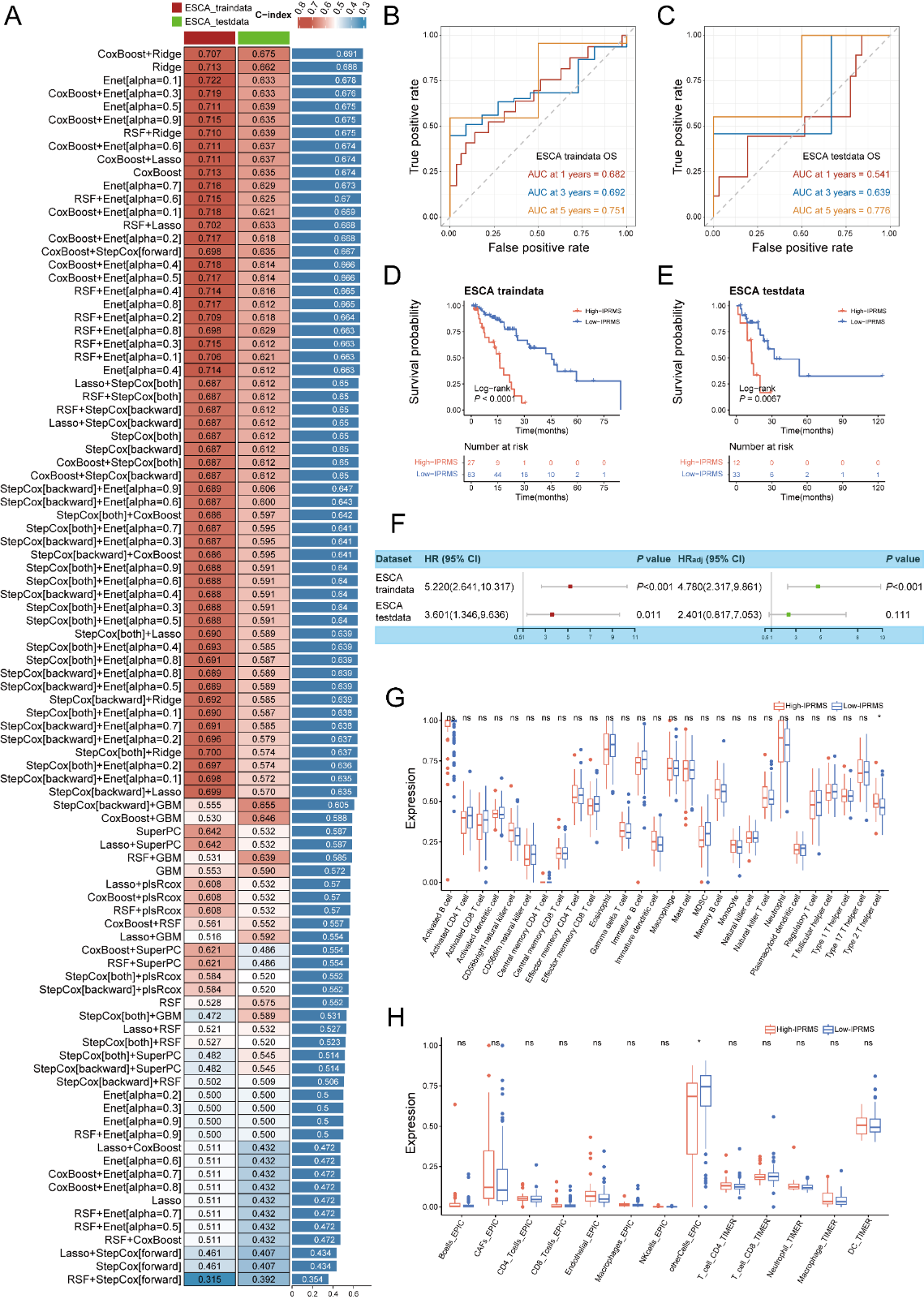


**Supplementary Figure 19 Construction of ESCA-IPRMS and its association with prognosis and** **immune infiltration.**

**A**. The C-index for traindata, testdata, and average C-index of each ML combination in ESCA. **B-C** ROC curve of the prognostic model based on IPRMS for ESCA traindata (B) and ESCA testdata (C). **D-E** Associations between ESCA-IPRMS and survival in ESCA traindata (D) and ESCA testdata (E). **F** Univariate and multivariate associations between IPRMS and OS, adjust factors were age, gender, race, and tumor stage. **G** The difference of infiltrating estimations of 28 immune cells in TME between High-IPRMS and Low-IPRMS groups. **H** The difference of abundance of immune cells between High-IPRMS and Low-IPRMS groups based on the EPIC and TIMER. * *P*<0.05; ** *P*<0.01; *** *P*<0.001


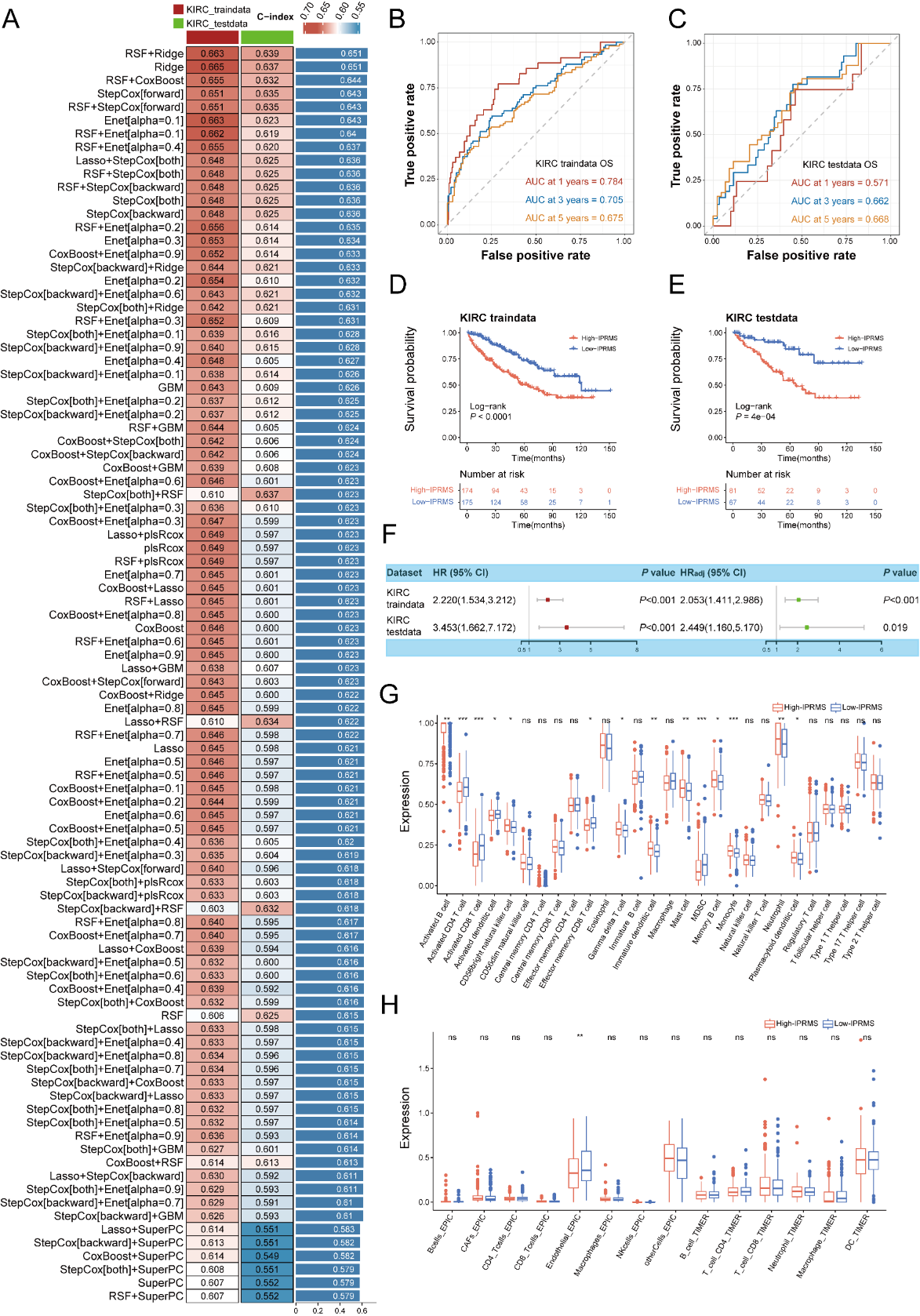


**Supplementary Figure 20 Construction of KIRC-IPRMS and its association with prognosis and immune infiltration.**

**A**. The C-index for traindata, testdata, and average C-index of each ML combination in KIRC. **B-C** ROC curve of the prognostic model based on IPRMS for KIRC traindata (B) and KIRC testdata (C). **D-E** Associations between KIRC-IPRMS and survival in KIRC traindata (D) and KIRC testdata (E). **F** Univariate and multivariate associations between IPRMS and OS, adjust factors were age, gender, race, and tumor stage. **G** The difference of infiltrating estimations of 28 immune cells in TME between High-IPRMS and Low-IPRMS groups. **H** The difference of abundance of immune cells between High-IPRMS and Low-IPRMS groups based on the EPIC and TIMER. * *P*<0.05; ** *P*<0.01; *** *P*<0.001


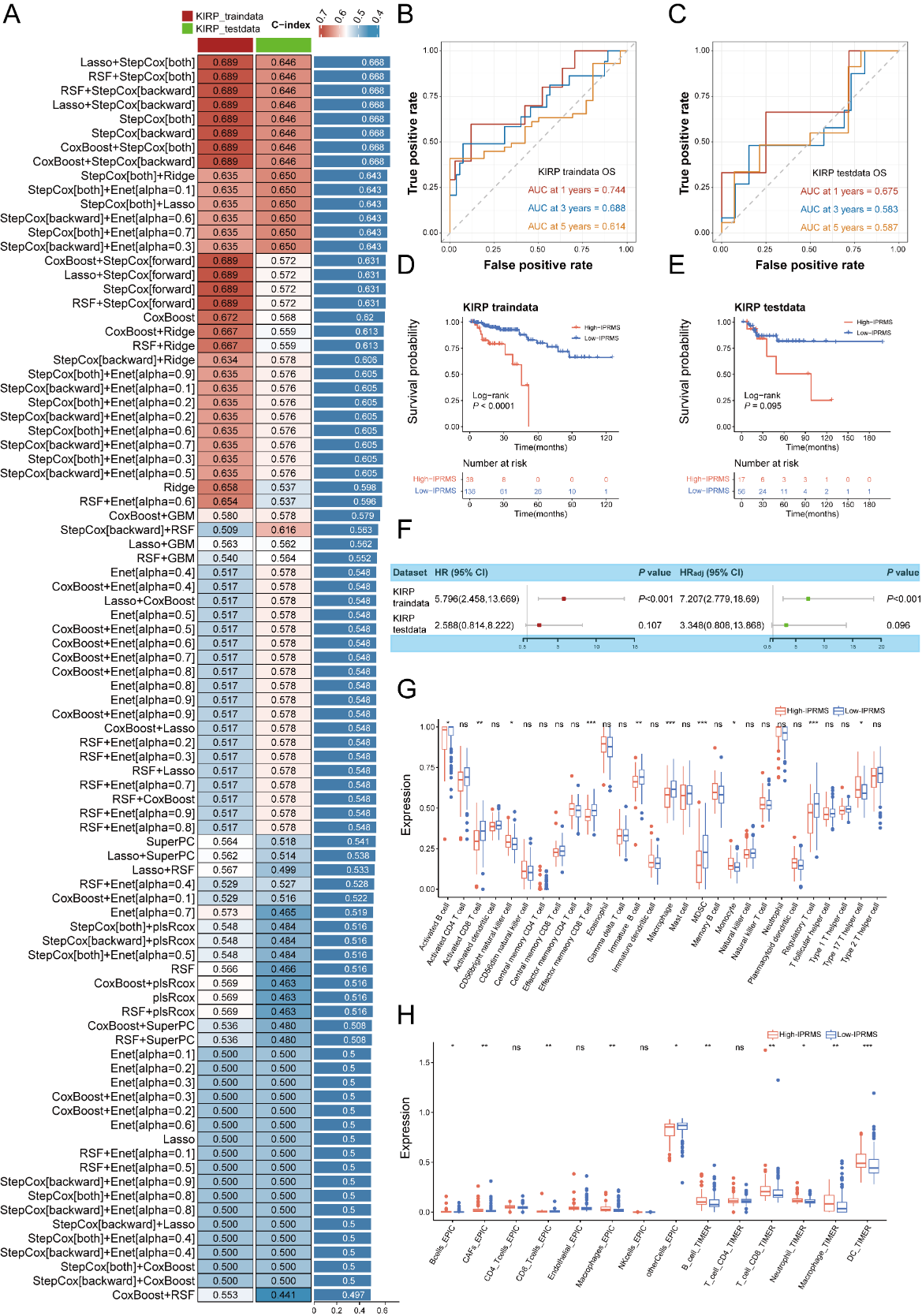


**Supplementary Figure 21 Construction of KIRP-IPRMS and its association with prognosis and immune infiltration.**

**A**. The C-index for traindata, testdata, and average C-index of each ML combination in KIRP. **B-C** ROC curve of the prognostic model based on IPRMS for KIRP traindata (B) and KIRP testdata (C). **D-E** Associations between KIRP-IPRMS and survival in KIRP traindata (D) and KIRP testdata (E). **F** Univariate and multivariate associations between IPRMS and OS, adjust factors were age, gender, race, and tumor stage. **G** The difference of infiltrating estimations of 28 immune cells in TME between High-IPRMS and Low-IPRMS groups. **H** The difference of abundance of immune cells between High-IPRMS and Low-IPRMS groups based on the EPIC and TIMER. * *P*<0.05; ** *P*<0.01; *** *P*<0.001


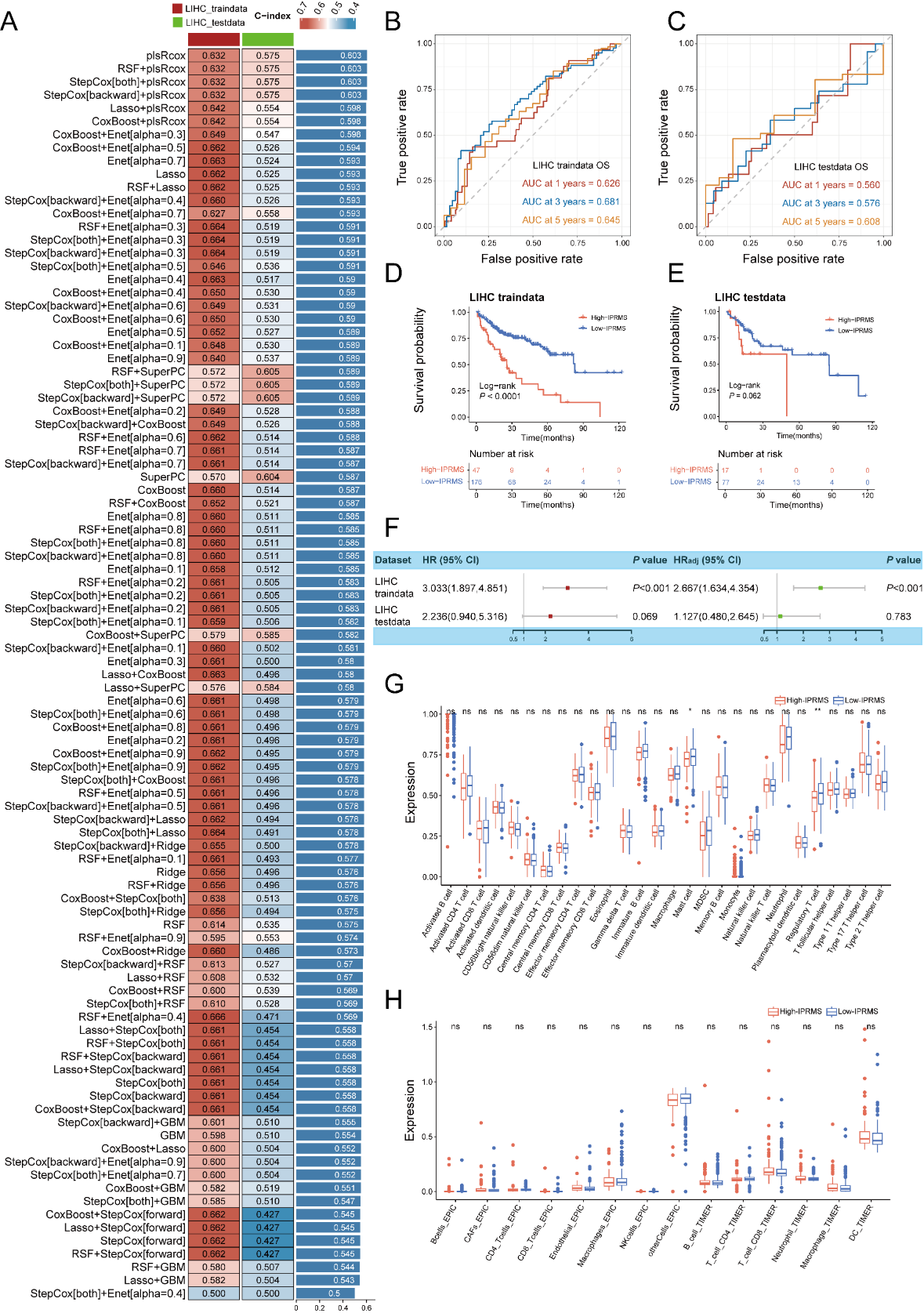


**Supplementary Figure 22 Construction of LIHC-IPRMS and its association with prognosis and immune infiltration.**

**A**. The C-index for traindata, testdata, and average C-index of each ML combination in LIHC. **B-C** ROC curve of the prognostic model based on IPRMS for LIHC traindata (B) and LIHC testdata (C). **D-E** Associations between LIHC-IPRMS and survival in LIHC traindata (D) and LIHC testdata (E). **F** Univariate and multivariate associations between IPRMS and OS, adjust factors were age, gender, race, and tumor stage. **G** The difference of infiltrating estimations of 28 immune cells in TME between High-IPRMS and Low-IPRMS groups. **H** The difference of abundance of immune cells between High-IPRMS and Low-IPRMS groups based on the EPIC and TIMER. * *P*<0.05; ** *P*<0.01; *** *P*<0.001
