# Supplementary material for: Effect of immune-related intratumoral microbiota and host gene expression on cancer prognosis
Source: mSystems. 2025 Sep 15;10(10):e01146-25. doi: 10.1128/msystems.01146-25 (PMC12542631; doi:10.1128/msystems.01146-25)
Supplement: Supplemental figures, part 3 — Fig. S23 to S32. [file msystems.01146-25-s0003.docx]

**Supplementary Figure 23-31**

**
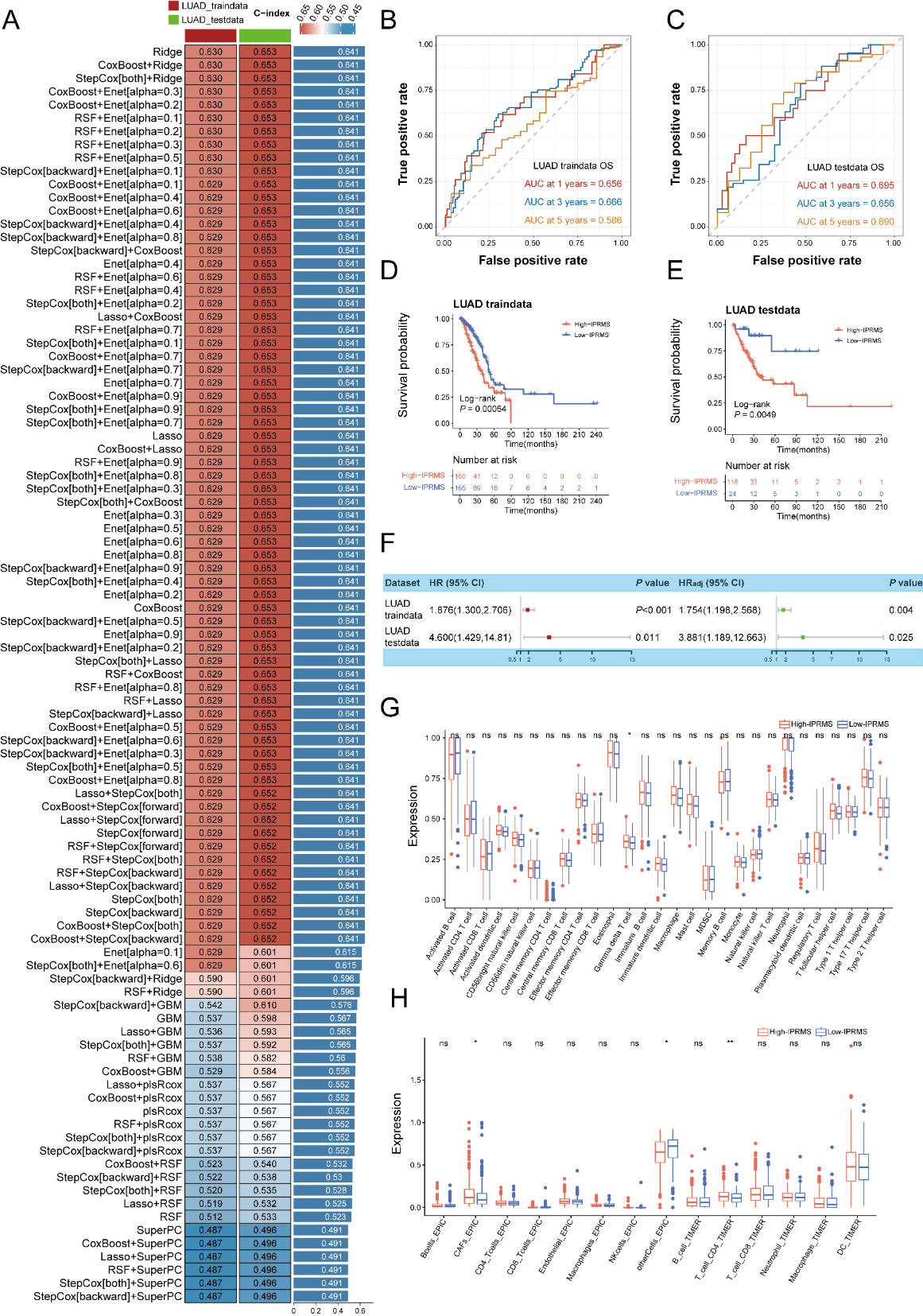
**

**Supplementary Figure 23 Construction of LUAD-IPRMS and its association with prognosis and immune infiltration.**

**A**. The C-index for traindata, testdata, and average C-index of each ML combination in LUAD. **B-C** ROC curve of the prognostic model based on IPRMS for LUAD traindata (B) and LUAD testdata (C). **D-E** Associations between LUAD-IPRMS and survival in LUAD traindata (D) and LUAD testdata (E). **F** Univariate and multivariate associations between IPRMS and OS, adjust factors were age, gender, race, and tumor stage. **G** The difference of infiltrating estimations of 28 immune cells in TME between High-IPRMS and Low-IPRMS groups. **H** The difference of abundance of immune cells between High-IPRMS and Low-IPRMS groups based on the EPIC and TIMER. * *P*<0.05; ** *P*<0.01; *** *P*<0.001

**
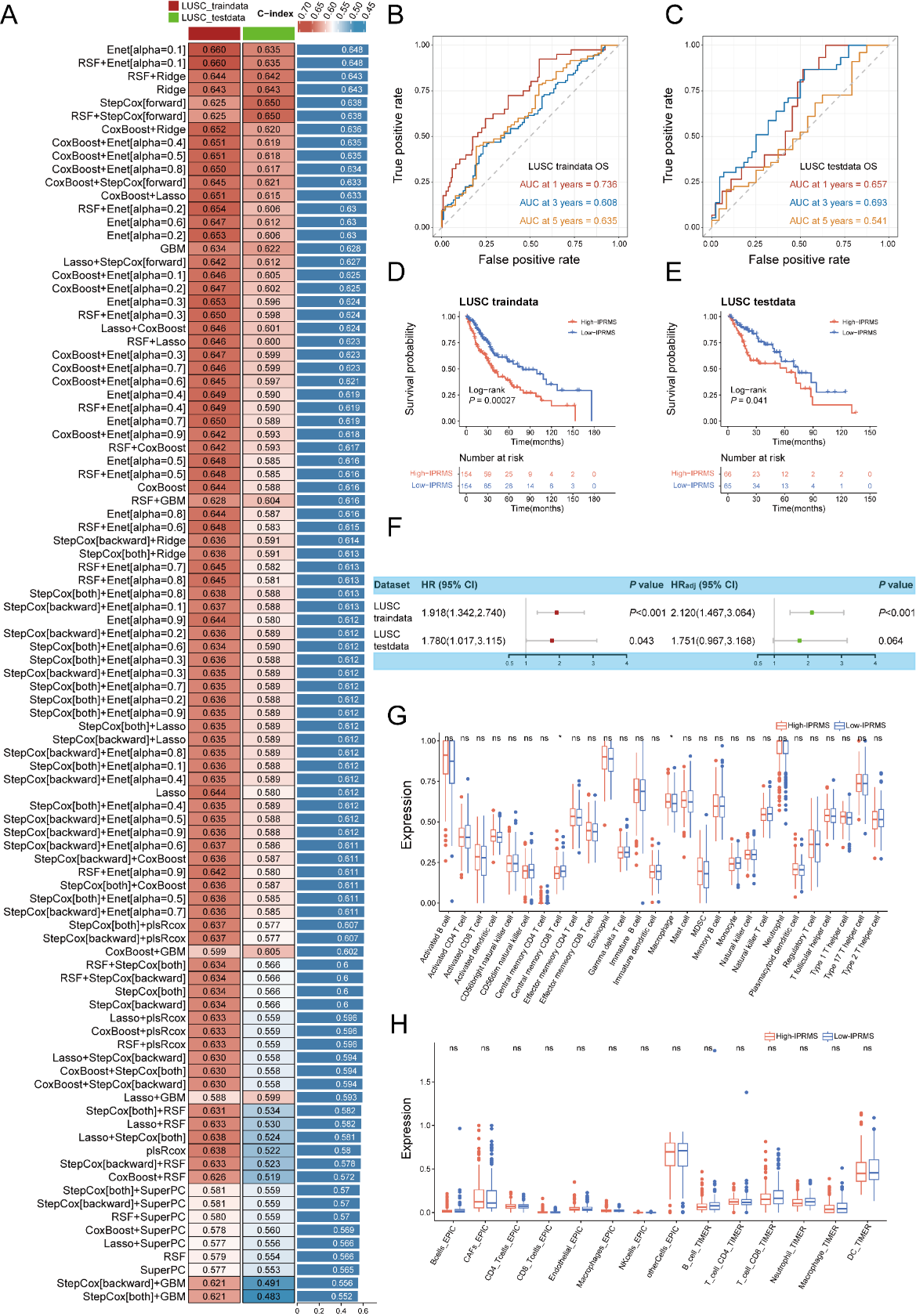
**

**Supplementary Figure 24 Construction of LUSC-IPRMS and its association with prognosis and immune infiltration.**

**A**. The C-index for traindata, testdata, and average C-index of each ML combination in LUSC. **B-C** ROC curve of the prognostic model based on IPRMS for LUSC traindata (B) and LUSC testdata (C). **D-E** Associations between LUSC-IPRMS and survival in LUSC traindata (D) and LUSC testdata (E). **F** Univariate and multivariate associations between IPRMS and OS, adjust factors were age, gender, race, and tumor stage. **G** The difference of infiltrating estimations of 28 immune cells in TME between High-IPRMS and Low-IPRMS groups. **H** The difference of abundance of immune cells between High-IPRMS and Low-IPRMS groups based on the EPIC and TIMER. * *P*<0.05; ** *P*<0.01; *** *P*<0.001

**
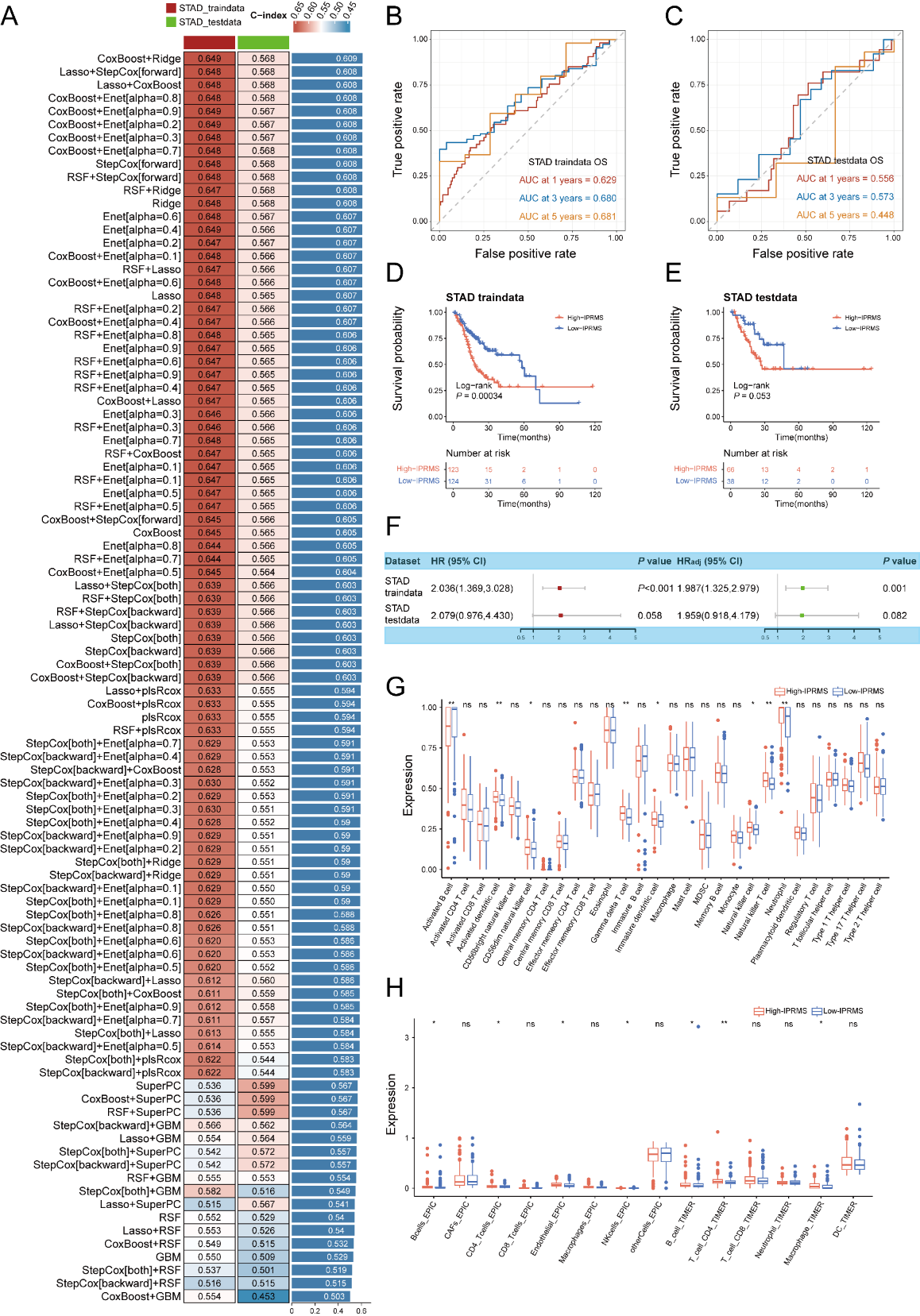
**

**Supplementary Figure 25 Construction of -STAD-IPRMS and its association with prognosis and immune infiltration.**

**A**. The C-index for traindata, testdata, and average C-index of each ML combination in STAD. **B-C** ROC curve of the prognostic model based on IPRMS for STAD traindata (B) and STAD testdata (C). **D-E** Associations between STAD-IPRMS and survival in STAD traindata (D) and STAD testdata (E). **F** Univariate and multivariate associations between IPRMS and OS, adjust factors were age, gender, race, and tumor stage. **G** The difference of infiltrating estimations of 28 immune cells in TME between High-IPRMS and Low-IPRMS groups. **H** The difference of abundance of immune cells between High-IPRMS and Low-IPRMS groups based on the EPIC and TIMER. * *P*<0.05; ** *P*<0.01; *** *P*<0.001

**
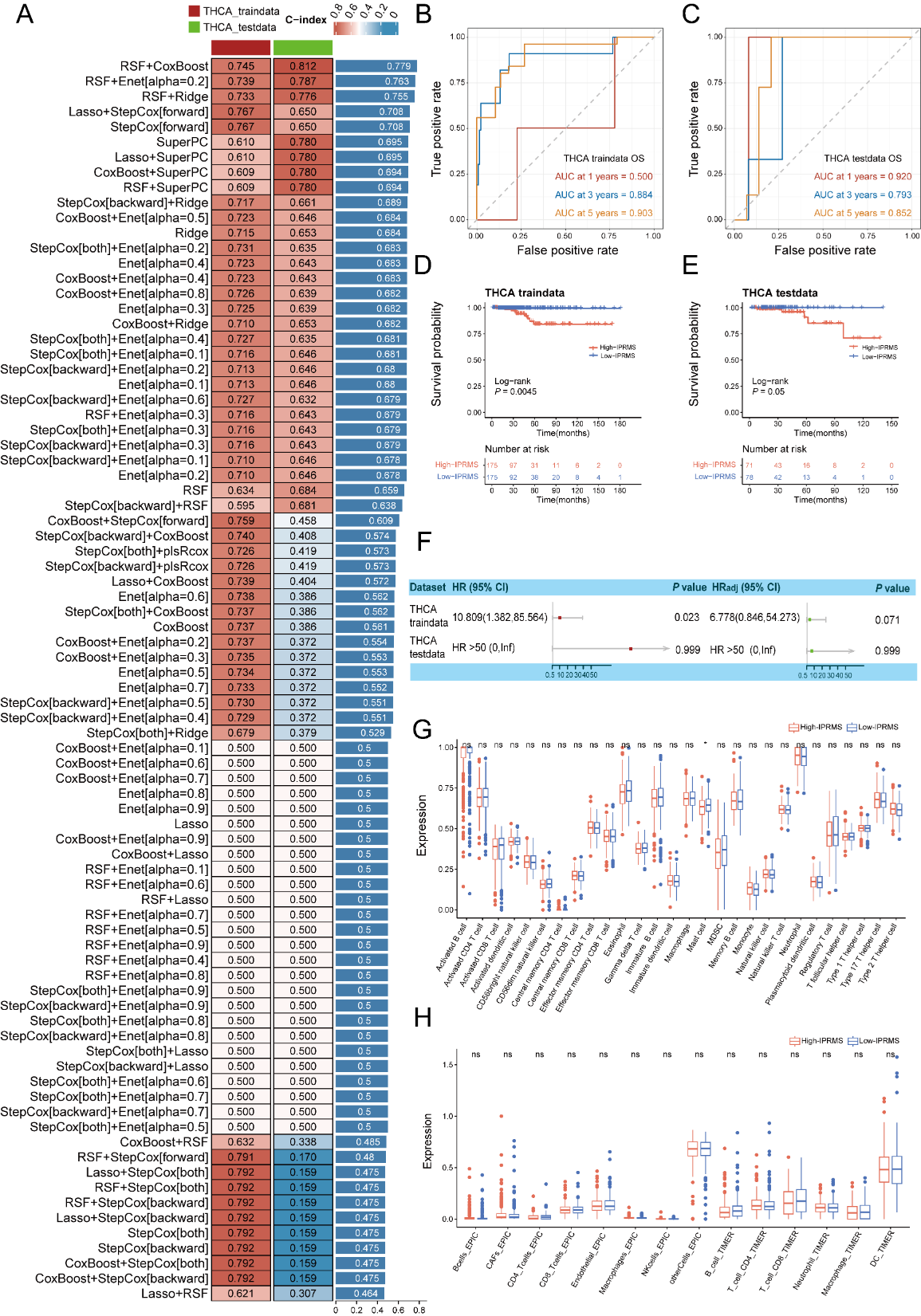
**

**Supplementary Figure 26 Construction of THCA-IPRMS and its association with prognosis and immune infiltration.**

**A**. The C-index for traindata, testdata, and average C-index of each ML combination in THCA. **B-C** ROC curve of the prognostic model based on IPRMS for THCA traindata (B) and THCA testdata (C). **D-E** Associations between THCA-IPRMS and survival in THCA traindata (D) and THCA testdata (E). **F** Univariate and multivariate associations between IPRMS and OS, adjust factors were age, gender, race, and tumor stage. **G** The difference of infiltrating estimations of 28 immune cells in TME between High-IPRMS and Low-IPRMS groups. **H** The difference of abundance of immune cells between High-IPRMS and Low-IPRMS groups based on the EPIC and TIMER. * *P*<0.05; ** *P*<0.01; *** *P*<0.001

**
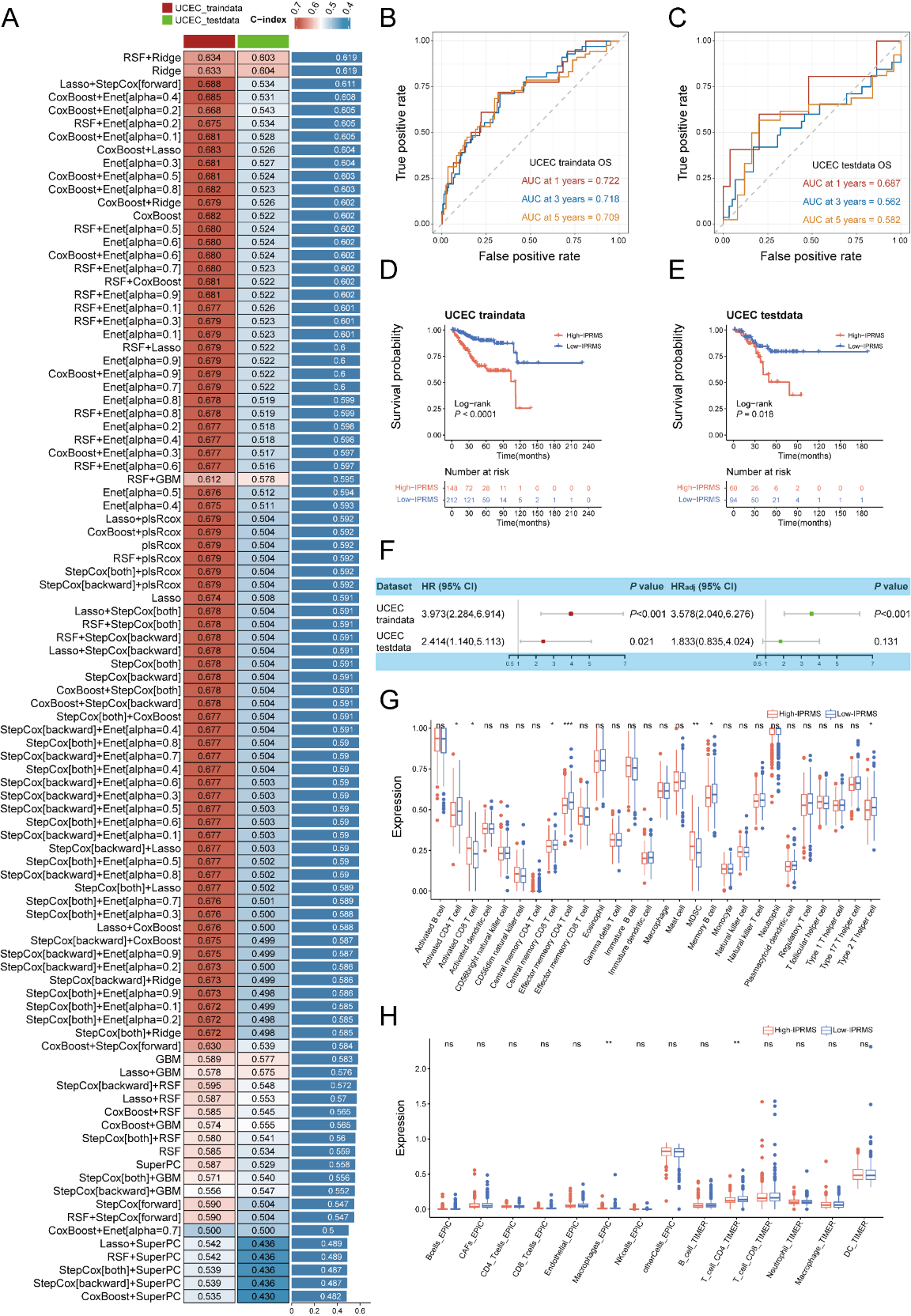
**

**Supplementary Figure 27 Construction of UCEC-IPRMS and its association with prognosis and immune infiltration.**

**A** The C-index for traindata, testdata, and average C-index of each ML combination in UCEC. **B-C** ROC curve of the prognostic model based on IPRMS for UCEC traindata (B) and UCEC testdata (C). **D-E** Associations between UCEC-IPRMS and survival in UCEC traindata (D) and UCEC testdata (E). **F** Univariate and multivariate associations between IPRMS and OS, adjust factors were age, race, and tumor stage. **G** The difference of infiltrating estimations of 28 immune cells in TME between High-IPRMS and Low-IPRMS groups. **H** The difference of abundance of immune cells between High-IPRMS and Low-IPRMS groups based on the EPIC and TIMER. * *P*<0.05; ** *P*<0.01; *** *P*<0.001


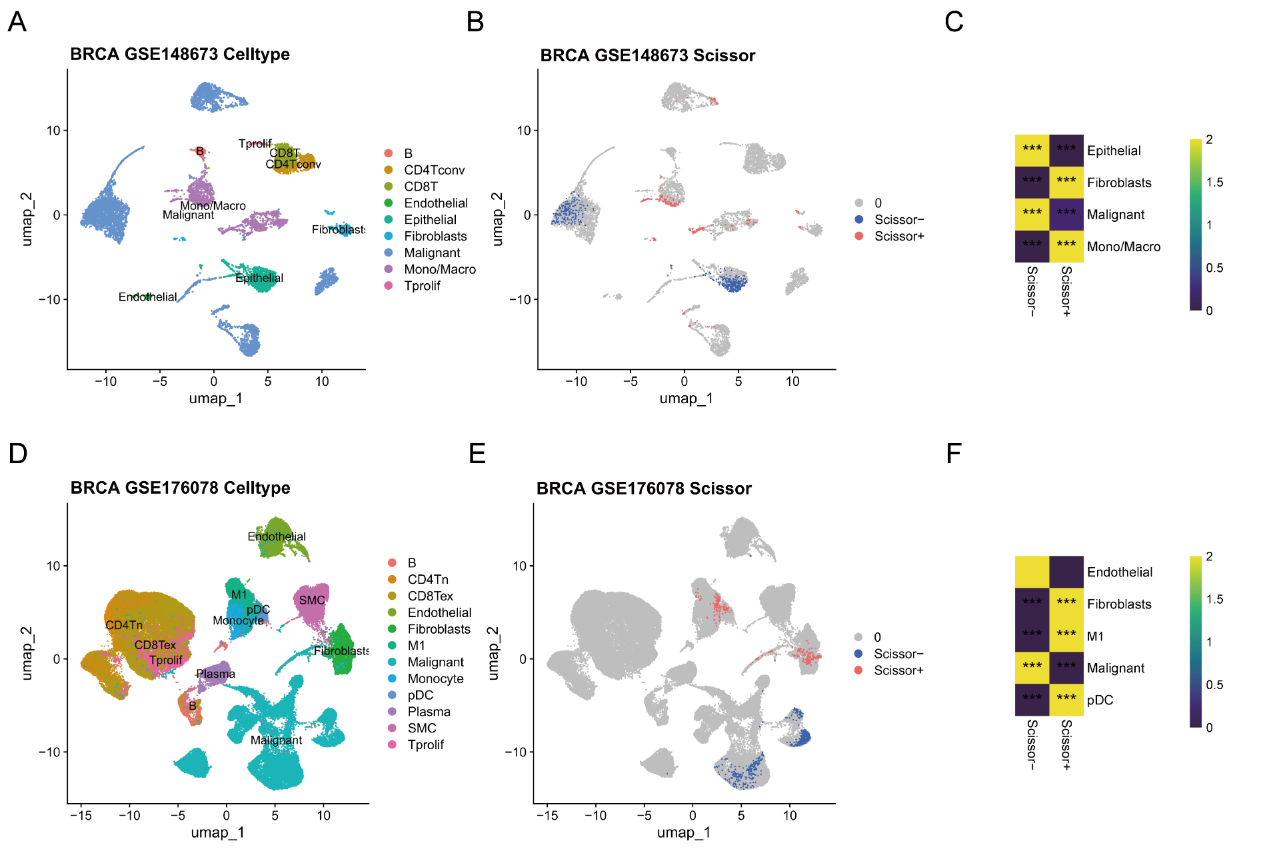


**Supplementary Figure 28 Scissor identification results on BRCA scRNA-seq data guided by TGCA-BRCA IPRMS phenotypes.**

**A** UMAP visualization of cell type annotation in BRCA_SC_GSE148673. **B** UMAP visualization of the Scissor-selected cells, the red and blue dots are cells associated with the High-IPRMS and Low-IPRMS phenotypes, respectively. **C** Heatmap showing the ORs of cell types occurring in BRCA_GSE148673 tissue. OR>1.5 and *P*<0.05 indicate that the cell type is preferred to enrich in the corresponding group. **D** UMAP visualization of cell type annotation in BRCA_SC_GSE176078. **E** UMAP visualization of the Scissor-selected cells, the red and blue dots are cells associated with the High-IPRMS and Low-IPRMS phenotypes, respectively. **F** Heatmap showing the ORs of cell types occurring in BRCA_GSE176078 tissue. OR>1.5 and *P*<0.05 indicate that the cell type is preferred to enrich in the corresponding group. If the OR>2, it is displayed as 2 in the heatmap. * OR>1.5 or OR<0.5 & *P*<0.05; ** OR>1.5 or OR<0.5 & *P*<0.01; *** OR>1.5 or OR<0.5 & *P*<0.001


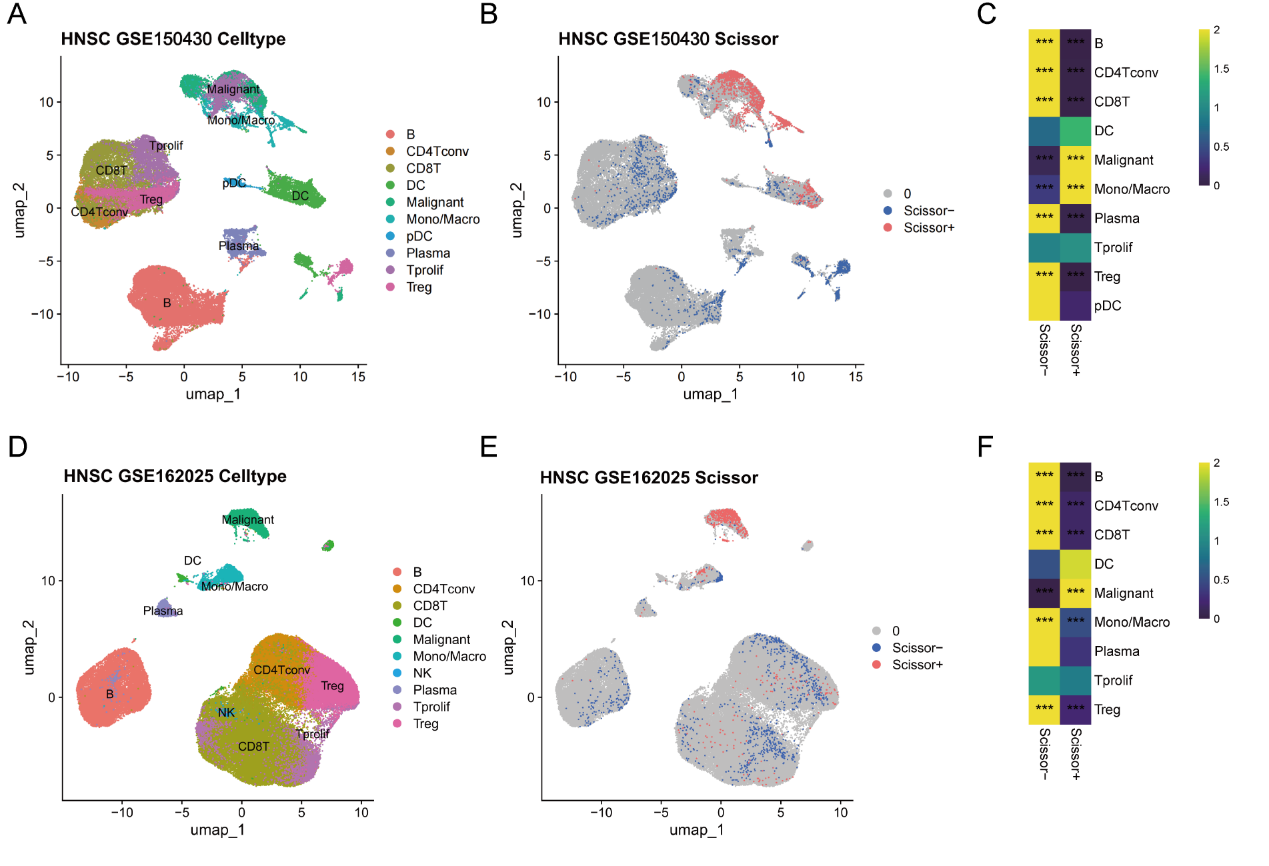


**Supplementary Figure 29 Scissor identification results on HNSC scRNA-seq data guided by TGCA- HNSC IPRMS phenotypes.**

**A** UMAP visualization of cell type annotation in HNSC_SC_GSE150430. **B** UMAP visualization of the Scissor-selected cells, the red and blue dots are cells associated with the High-IPRMS and Low-IPRMS phenotypes, respectively. **C** Heatmap showing the ORs of cell types occurring in HNSC_GSE150430 tissue. OR>1.5 and *P*<0.05 indicate that the cell type is preferred to enrich in the corresponding group. **D** UMAP visualization of cell type annotation in HNSC_SC_GSE162025. **E** UMAP visualization of the Scissor-selected cells, the red and blue dots are cells associated with the High-IPRMS and Low-IPRMS phenotypes, respectively. **F** Heatmap showing the ORs of cell types occurring in HNSC_GSE162025 tissue. OR>1.5 and *P*<0.05 indicate that the cell type is preferred to enrich in the corresponding group. If the OR>2, it is displayed as 2 in the heatmap. * OR>1.5 or OR<0.5 & *P*<0.05; ** OR>1.5 or OR<0.5 & *P*<0.01; *** OR>1.5 or OR<0.5 & *P*<0.001


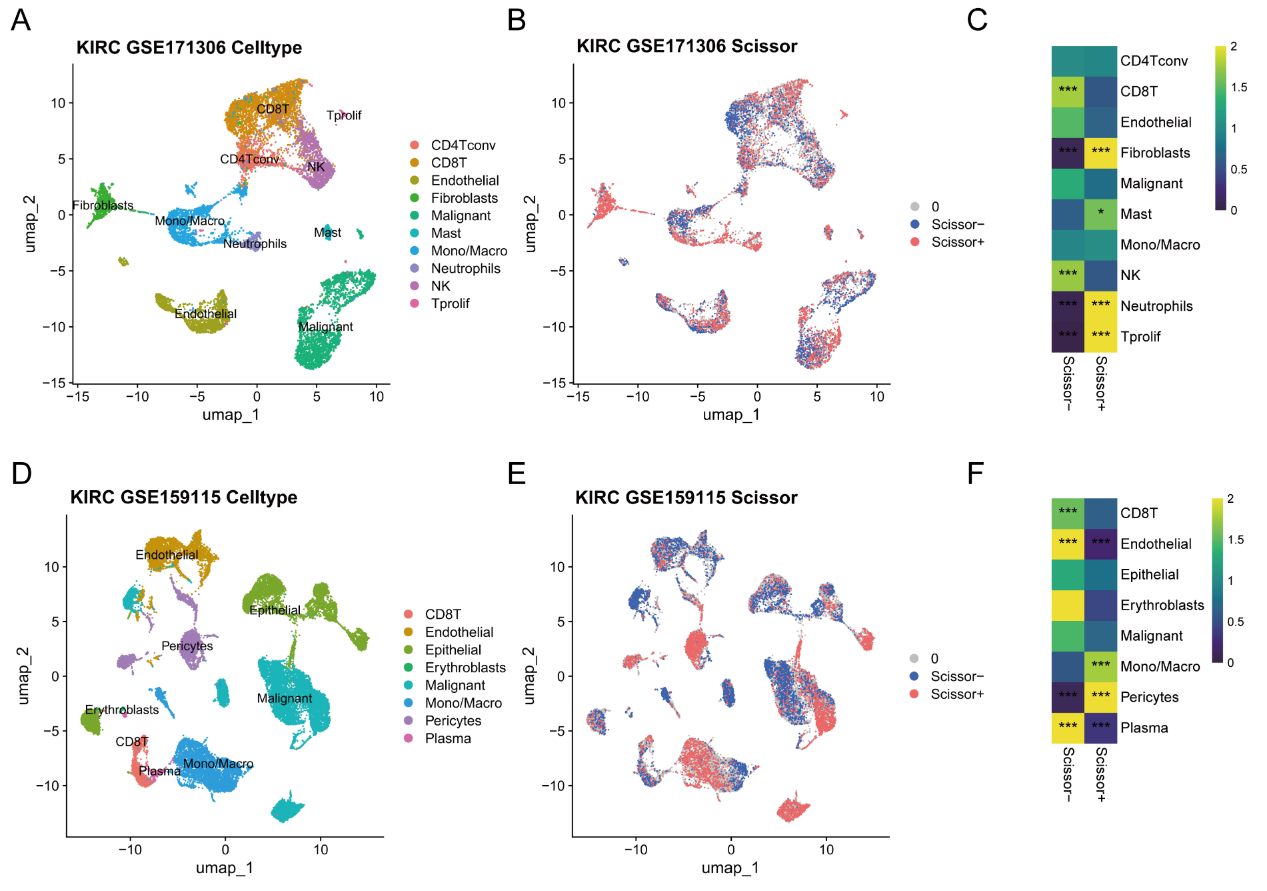


**Supplementary Figure 30** **Scissor identification results on KIRC scRNA-seq data guided by TGCA-KIRC IPRMS phenotypes.**

**A** UMAP visualization of cell type annotation in KIRC_SC_GSE171306. **B** UMAP visualization of the Scissor-selected cells, the red and blue dots are cells associated with the High-IPRMS and Low-IPRMS phenotypes, respectively. **C** Heatmap showing the ORs of cell types occurring in KIRC_GSE171306 tissue. OR>1.5 and *P*<0.05 indicate that the cell type is preferred to enrich in the corresponding group. **D** UMAP visualization of cell type annotation in KIRC_SC_GSE171306. **E** UMAP visualization of the Scissor-selected cells, the red and blue dots are cells associated with the High-IPRMS and Low-IPRMS phenotypes, respectively. **F** Heatmap showing the ORs of cell types occurring in KIRC_GSE159115 tissue. OR>1.5 and *P*<0.05 indicate that the cell type is preferred to enrich in the corresponding group. If the OR>2, it is displayed as 2 in the heatmap. * OR>1.5 or OR<0.5 & *P*<0.05; ** OR>1.5 or OR<0.5 & *P*<0.01; *** OR>1.5 or OR<0.5 & *P*<0.001


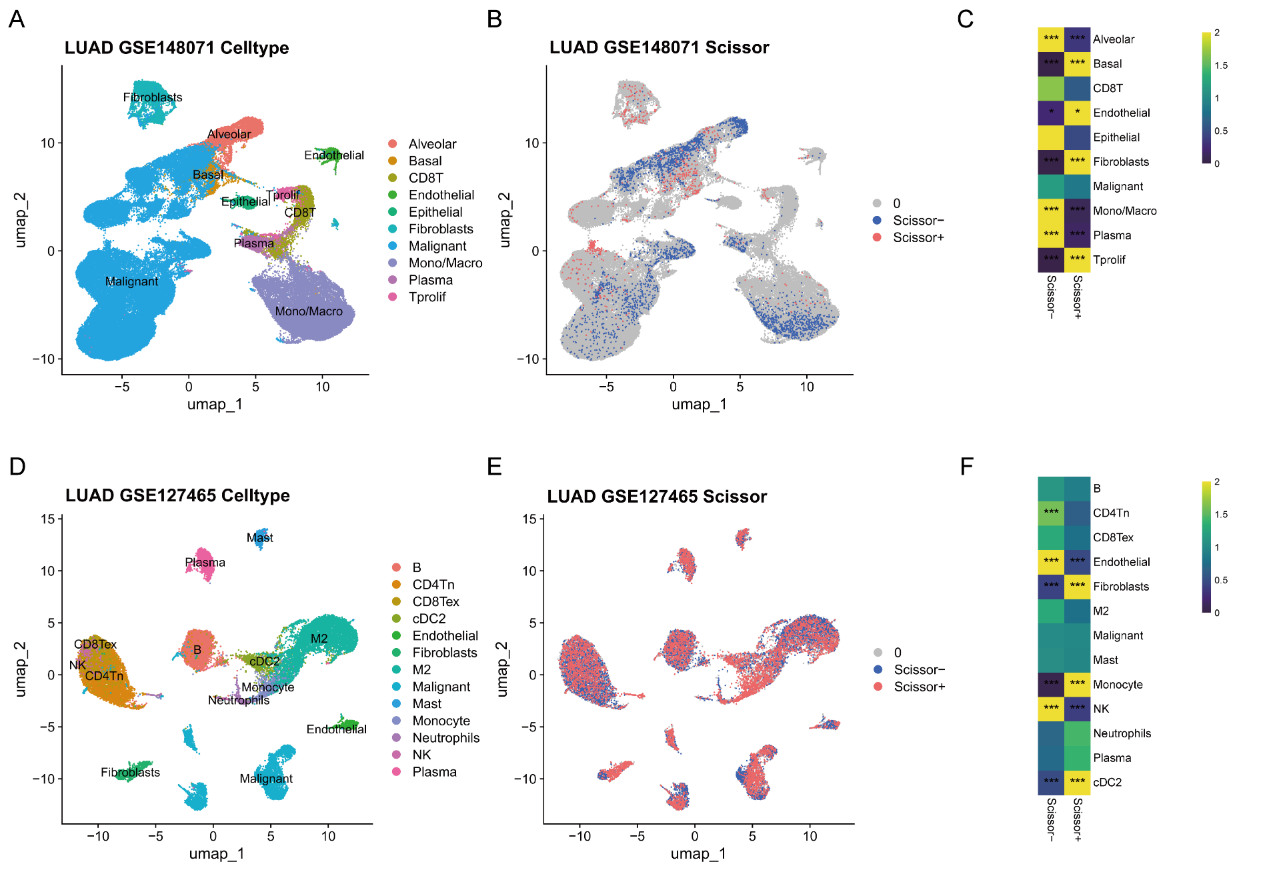


**Supplementary Figure 31 Scissor identification results on LUAD scRNA-seq data guided by TGCA-LUAD IPRMS phenotypes.**

**A** UMAP visualization of cell type annotation in LUAD_SC_GSE148071. **B** UMAP visualization of the Scissor-selected cells, the red and blue dots are cells associated with the High-IPRMS and Low-IPRMS phenotypes, respectively. **C** Heatmap showing the ORs of cell types occurring in LUAD_GSE148071 tissue. OR>1.5 and *P*<0.05 indicate that the cell type is preferred to enrich in the corresponding group. **D** UMAP visualization of cell type annotation in LUAD_SC_GSE127465. **E** UMAP visualization of the Scissor-selected cells, the red and blue dots are cells associated with the High-IPRMS and Low-IPRMS phenotypes, respectively. **F** Heatmap showing the ORs of cell types occurring in LUAD_GSE127465 tissue. OR>1.5 and *P*<0.05 indicate that the cell type is preferred to enrich in the corresponding group. If the OR>2, it is displayed as 2 in the heatmap. * OR>1.5 or OR<0.5 & *P*<0.05; ** OR>1.5 or OR<0.5 & *P*<0.01; *** OR>1.5 or OR<0.5 & *P*<0.001


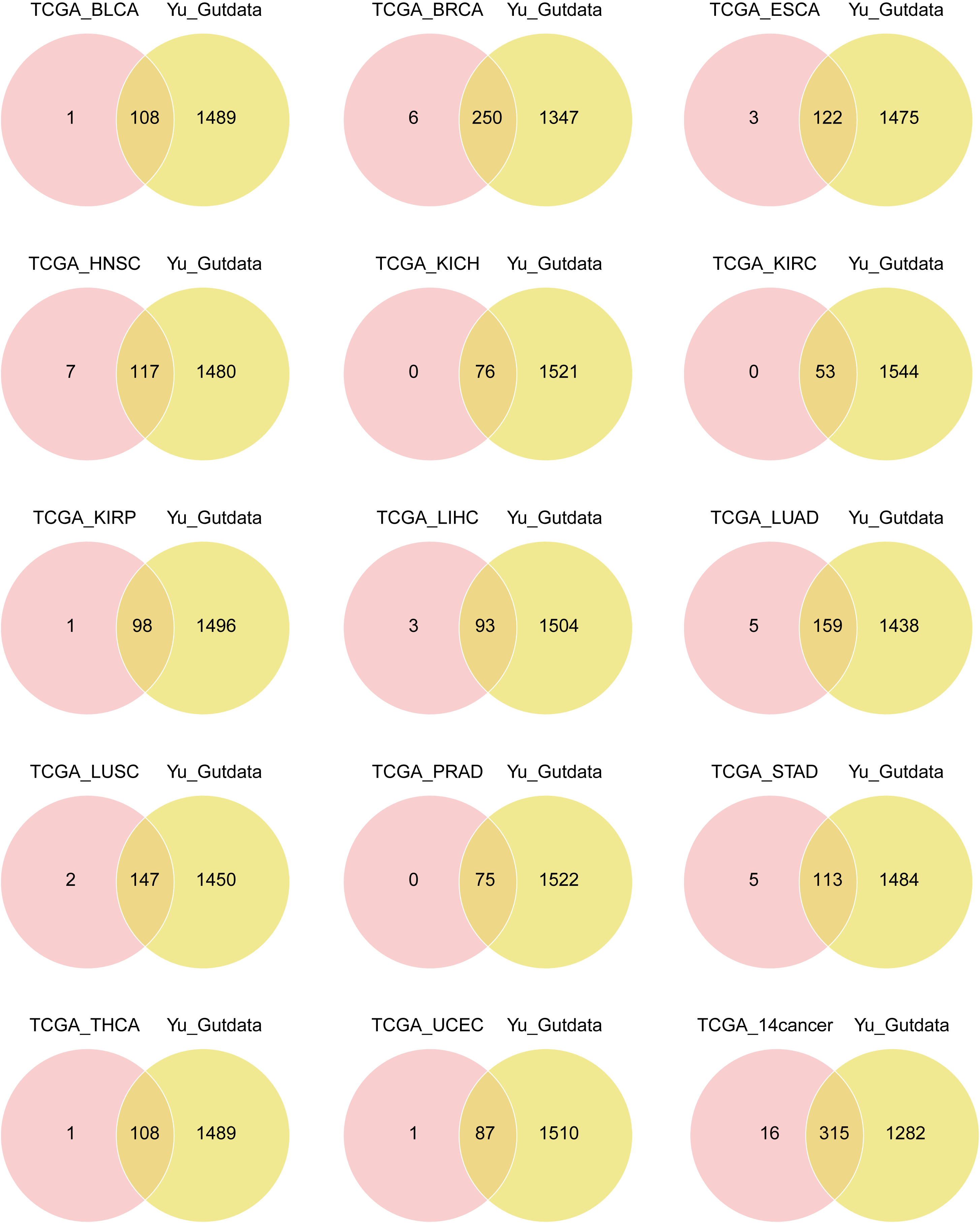


**Supplementary Figure 32 The comparison between TCGA intratumoral microbiota and gut microbiota.**

Venn diagrams respectively show the comparison of the list of intratumoral microbiota genera in TCGA after filtering for BLCA, BRCA, ESCA, HNSC, KICH, KIRC, KIRP, LIHC, LUAD, LUSC, PRAD, STAD, THCA and UCEC, as well as the combined TCGA_14cancer of 14 types of cancers, with the list of gut microbiota genera by Yu et al.
