# Supplementary material for: Effect of immune-related intratumoral microbiota and host gene expression on cancer prognosis
Source: mSystems. 2025 Sep 15;10(10):e01146-25. doi: 10.1128/msystems.01146-25 (PMC12542631; doi:10.1128/msystems.01146-25)
Supplement: Supplemental text — Detailed additional methodology. [file msystems.01146-25-s0004.docx]

**Supplementary materials**

**The dataset filtering step of the BIC dataset**

The BIC database primarily focuses on profiling tissue-resident bacteria from TCGA miRNA-seq data. The BIC dataset adopted relatively strict sample and microbial filtering steps. Its data filtering steps were as follows:

(1) Formalin-fixed paraffin-embedded (FFPE) samples were excluded due to known biases in microbial profiles.

(2) Duplicate samples (from the same patient with different analyte codes or plate IDs) were removed, retaining only one sample per patient based on priority criteria (e.g., analyte code “R” over “T”, higher plate ID).

(3) Only primary tumor (solid tissue) and adjacent normal samples were retained.

(4) Taxa description containing “Bacteria;;;;;;” or Archaea is neglected were removed, and empty taxa is categorized as “known” in each taxonomy level were removed.

After extracting bacterial abundance data from BIC, we further filtered out the low abundance microbes that may represent noise or residual contamination for downstream analysis. Specifically, we retained taxa only if they satisfied: relative abundance ≥ 0.0005 in at least 10% of samples. we applied an additional taxon-level filter to remove low-abundance microbes

**Analysis of the overall correlations between host gene expression and intratumoral microbiota**

Before identifying the associations between subsets of intratumoral microbiota and host gene subsets (group-level associations), we first analyzed the overall correlations between the microbiota and host gene expression in each tumor. This is because if no significant overall correlations (FDR>0.1) exist between microbiota and host gene expression, it is more likely that associations may be found between their subsets. We applied Procrustes analysis and the Mantel test to assess the overall correlations between host gene expression and intratumoral microbiota using R “vegan” package (1).To assess the correlations, we used M^2^ and r as statistical metrics, respectively. A lower M^2^ value or a higher r value indicates a stronger correlation. We used Aitchison’s distance for gene expression data and Bray-Curtis distance for intratumoral microbiota data. Compared with the Mantel test, the Procrustes analysis is a more powerful and accurate method for measuring the similarity between datasets. Therefore, in the main text, we describe the results based on the Procrustes analysis (2).

**Sparse canonical correlation analysis (CCA) and Lasso penalized regression**

The multi-omics machine learning framework developed by Priya et al. for analyzing the microbiota and host transcriptome mainly includes two important algorithms: Sparse canonical correlation analysis model and Lasso penalized regression. (3) In summary, the steps for identifying the associations between intratumoral microbes and host gene expression in this study are as follows:

(1) Data filtering and standardization: 1) The host RNA-Seq data were filtered to retain protein-coding genes and genes expressed in more than 50% of the samples in each tumor. Then, the filtered gene expression data (count value) were transformed to variance-stabilizing transformation using the DESeq2 package.(4) A cutoff of 25% variance quantile was used to filter out genes with low variance in each tumor. Finally, unique host gene expression matrices for each tumor were obtained for downstream analysis. 2) We combined matrices of taxonomic data summarized at different taxonomic levels (genus, family, order, class and phylum taxonomic levels) into a combined taxonomic matrix. Then, the summarized taxonomic matrix (count data) was concatenated with the combined taxonomic matrix. The resulting matrix was subjected to centered log-ratio (CLR) transformation for downstream analysis.

(2) Sparse CCA: This method was used to identify host gene subsets related to intratumoral microbiota to characterize group-level associations. The main steps included: 1) hyperparameter tuning and fitting for Sparse CCA model, 2) identification of Sparse CCA model components (microbes-host gene subsets associations), 3) pathway enrichment analysis for gene subsets within each component, 4) differential enrichment analysis of pathways between tumor tissues and adjacent normal tissues across 14 tumors, 5) identification of cancer-specific host pathways and cancer-shared host pathways.

(3) Lasso penalized regression: This method was used to identify specific associations between individual host gene and intratumoral microbe: 1) Lasso regression analysis; 2) Stability selection for the Lasso model; 3) Comparing tumor tissues versus adjacent normal tissues associations, and identification of specific microbe-gene associations that were found in tumor tissues but not in adjacent normal tissues; 4) Pathway enrichment analysis for genes in microbe-gene associations, and identifying cancer-specific pathways, as well as cancer-shared pathways.

Specific details are available in the research article by Priya et al., and the parameters of the two algorithms in this study are consistent with those used in Priya’s work.

**Survival mediation analysis (SMA)**

Based on the VanderWeele's SMA, we focused on the ways in which intratumoral microbes affect host prognosis by influencing three distinct types of mediators:

(1) IPRMS-immune cell-OS SMAs: We utilized the gene markers of 28 immune cells from the referenced study (<https://www.cell.com/cms/10.1016/j.celrep.2016.12.019/attachment/f353dac9-4bf5-4a52-bb9a-775e74d5e968/mmc3.xlsx>), and employed the Gene Set Variation Analysis (GSVA) algorithm to calculate the abundance of these immune cells. Using the abundances of each immune cell as a mediator, with pan-cancer IPRMS as the exposure, and host OS as the outcome, we fit SMA models to explore whether microbes could influence host prognosis by affecting infiltration of immune cells.

(2) Microbes-pathway-OS SMAs: Based on the “group-level associations”, we identified components containing multiple intratumoral microbial subsets and their related host gene subsets, annotate the gene subsets in each component to identify the host pathways associated with the intratumoral microbial subsets within that component. Subsequently, for each pathway in every component, we extracted the microbes-related genes that were enriched in a specific pathway, and used the Ridge Cox algorithm to calculate a score for this pathway (referred to pathway-score), characterizing the level of pathway enrichment and considering it as a mediator of SMA. Simultaneously, we used the Ridge Cox algorithm to construct a microbe score (referred to microbes-score) within the same component, characterizing the assessment metrics for microbial exposure and considering this score as an exposure factor for SMA. Using the microbe-pathway-OS SMAs, we explored whether microbes could affect host prognosis by influencing the regulation of specific pathways. The formulas for calculating the pathway-score and microbes-score are similar to that of IPRMS.

(3) Microbe-gene-OS SMAs: We identified microbe-related genes from the “individual microbe-host gene associations” section as mediators (gene expression was normalized by log2(TPM+1)), with microbes associated with gene expression levels as exposures (microbial abundance was normalized by CLR), to investigate whether a particular microbe can influence host prognosis by affecting the expression of related host genes.

SMAs conducted using both delta method and bootstrap method (*P*<0.05). The confounding factors controlled for in fitting the three types of SMAs included age, gender (except BRCA and UCEC, which did not control for gender), race, and tumor stage.

(1) Bulk RNA Sequencing: Among the 14 tumor types, sparse canonical correlation analysis (sparse CCA) identified more than 20 microbial-associated pathways in 11 cancer types: bladder urothelial carcinoma (BLCA), breast cancer (BRCA), esophageal carcinoma (ESCA), head and neck squamous cell carcinoma (HNSC), kidney renal clear cell carcinoma (KIRC), kidney renal papillary cell carcinoma (KIRP), liver hepatocellular carcinoma (LIHC), lung adenocarcinoma (LUAD), prostate adenocarcinoma (PRAD), thyroid carcinoma (THCA), and uterine corpus endometrial carcinoma (UCEC). To validate the microbiota-related associations between intratumoral microbiota and host gene expression observed in TCGA, we systematically searched for eligible datasets in the SRA and GEO databases based on the following inclusion criteria:

1) Initial screening in the SRA database was conducted using the following query pattern for each tumor type: ((((((Tumor name) AND TRANSCRIPTOMIC[Source]) AND cDNA) AND RNA-Seq[Strategy]) AND Homo sapiens[Organism]) AND PAIRED[Layout]).

2) Datasets must be publicly accessible, available in FASTQ format, and generated using the Illumina sequencing platform.

3). Only datasets containing at least 20 samples each of tumor tissue or adjacent/normal tissue were included.

4). Datasets published within the last five years were selected to ensure data relevance and timeliness.

5). Only primary tumors without prior treatment or intervention were considered.

6). For the GEO database, only datasets with RNA-seq count values of NCBI-generated data were selected.

Ultimately, we selected 11 GEO-tumor datasets to validate the microbe-host gene expression associations at the bulk level:

1) BLCA-GSE236932 (39 tumor tissues and 14 paired adjacent tissues, PE150) (5),

2) BRCA-GSE233242 (43 pairs, PE75) (6),

3) ESCA-GSE130078 (23 pairs, PE100) (7);

4) HNSC-GSE178537 (35 tumor tissues and 21 paired adjacent tissues, PE75 and PE100) (8);

5) KIRC-GSE126964 (55 tumor tissues and 11 paired adjacent tissues, PE150) (9);

6) KIRP-GSE180777 (43 tumor tissues and 45 paired adjacent tissues, PE150) (10);

7) LIHC-GSE214846 (65 pairs, PE150) (11);

8) LUAD-GSE233774 (30 pairs, PE150) (12);

9) PRAD-GSE237995 (63 tumor tissues and 58 paired adjacent tissues, PE75) (13);

10) THCA-GSE83520 (12 pairs, PE100, The THCA dataset did not find any datasets that met the screening conditions, so the GSE83520 dataset is temporarily used for analysis) (14);

11) UCEC-GSE146889 (37 tumor tissues and 35 paired adjacent tissues, PE100) (15).

For subsequent analysis, we downloaded FASTQ data from the SRA database to identify the intratumoral microbiota. To reduce heterogeneity in the upstream host gene expression analysis, we used the NCBI-generated data for each GEO-dataset.

(2) Single-cell RNA sequencing (scRNA-seq): TISCH2 (Tumor Immune Single-cell Hub 2) is a scRNA-seq database designed to study the tumor microenvironment (TME) (16). It collects scRNA-seq data from 20 types of tumors, and provides detailed cell-type annotations at the single-cell level, along with comprehensive sample information after uniform filtering. We selected scRNA-seq data for BRCA, HNSC, KIRC, and LUAD from TISCH2. In these tumors, the prognosis was associated with IPRMS and exhibited differences in immune infiltration across different IPRMS groups. The criteria of selection are as follows: 1) Selecting samples from untreated primary tumor tissues and adjacent normal tissues; 2) A total cell counts exceeding 10,000; 3) Inclusion of malignant cells in the sample; 4) Priority was given to data sourced from the 10X Genomics platform. Ultimately, we selected two scRNA-seq datasets for each of the four tumors, which are BRCA_SC_GSE148673 (6 samples, 10,359 cells),(17) BRCA_SC_GSE176078 (26 samples, 89,471 cells),(18) HNSC_SC_GSE150430 (15 samples, 45,959 cells),(19) HNSC_SC_GSE162025 (10 samples, 82,616 cells),(20) KIRC_SC_GSE171306 (2 samples, 11,427 cells),(21) KIRC_SC_GSE159115 (7 samples, 27,669 cells),(22) LUAD_SC_GSE148071 (42 samples, 82,267 cells, GEXSCOPETM platform),(23) and LUAD_SC_GSE127465 (7 samples, 23,999 cells).(24)

**The processing and analysis of bulk RNA sequence data**

First, Fqtrim (v0.9.7) was used to filter low-quality reads from the FASTQ files (BRCA-GEO parameters: -l 40 -m 3 -q 25; ESCA-GEO parameters: -l 50 -m 3 -q 25; parameters for the other four tumors: -l 60 -m 3 -q 25).(25) Then, HISAT2 (v2.2.1) (26) with default parameters was used to map the reads from each sample to the reference genome GRCh38 (Genome Reference Consortium Human Build 38.113). Subsequently, the reads mapped to GRCh38 were discarded. Remaining reads were mapped to the NCBI Microbial Reference Genome Database using Kraken2 (v2.1.2) with default parameters, which includes databases for “archaea”, “bacteria”, “plasmid”, “viral”, “human”, “fungi”, “protozoa”, and “UniVec_Core”.(27) Bracken (v2.5) was used to estimate the abundance of each sample at the bacterial taxonomic levels of phylum, class, order, family, and genus (BRCA-GEO parameters: -l 75; ESCA-GEO parameters: -l 100; parameters for the other four tumors: -l 150) (28). Subsequently, we used intratumoral microbiota data for downstream analysis.

In analyzing the associations between host gene expression and intratumoral microbiota, the normalization and analysis of host RNA-seq data were consistent with those of TCGA. Intratumoral microbiota data were filtered using two thresholds: (1) retaining microbes with a relative abundance exceeding 0.0005 in at least 10% of the samples (strict threshold), and (2) retaining microbes with a relative abundance exceeding 0.0001 in at least 5% of the samples (lenient threshold). The subsequent normalization and analysis were consistent with TCGA analysis.

Subsequently, we used Sparse CCA to analyze the association between intratumoral microbiota and host gene expression in six tumors, and performed pathway enrichment analysis on the subset of host genes associated with microbes. To compare the similarity of host pathways related to intratumoral microbiota between TCGA and GEO, we defined an overlap index between microbes-related pathways derived from the TCGA database and those from the GEO database, following a method similar to that used by Priya et al. The overlap index was calculated as the number of common pathways between the two databases in the same tumor types, divided by the number of pathways in the database with fewer pathways. The Pathway Overlap Index (POI) is represented as follows:

$$\text{Pathway overlap }\left( X\text{, }Y \right)\text{=}\frac{\left| X\bigcap Y \right|}{\min\left( \left| X \right|, \left| Y \right| \right)}$$

**Exploring the association between intratumoral microbiota and host gene expression at the single-cell level using the SAHMI algorithm.**

In this study, we not only analyzed the associations between the host and microbiota at the bulk level, but also explored them at the single-cell level. Since the largest number of microbiota-related host pathways (124 pathways) were identified at the bulk level in LUAD, the publicly available single-cell RNA sequencing datasets from LUAD were analyzed next. We conducted a single-cell level association analysis of intratumoral microbiota and host gene expression using single-cell sequencing data from 8 tumor tissues and 4 adjacent normal tissues in the GSE123902 dataset (PRJNA510249, 10X Genomics platform) (29). Host single-cell RNA-seq was analyzed using the Seurat package (v5.2.1), while the analysis of microbial data referred to the SAHMI algorithm. The SAHMI (single-cell analysis of host-microbiome interactions) algorithm is a powerful tool for analyzing the association between host and microbiota in single-cell sequencing data (30). It can identify co-localized microbiota with each host cell subpopulation under the premise of denoising and removing potential contaminants, and analyze their association with the molecular and clinical characteristics of host cell subtypes. The SAHMI process is divided into the following 8 steps (<https://github.com/sjdlabgroup/SAHMI>):

1) Taxonomic classification (recommended with Kraken2);

2) Extract microbiome reads;

3) Single-cell k-mer analysis;

4) Barcode level signal denoising (barcode k-mer correlation test);

5) Sample-level signal denoising (sample k-mer correlation tests);

6) Identifying contaminants and false positives (cell line quantile test);

7) Quantitation of microbes and creating the barcode-metagenome counts matrix;

8) Joint analysis of host and microbial data.

The specific steps for analyzing the association between intratumoral microbiota and the host at the single-cell level are as follows:

(1) Filter human sequences based on the GRCh38 genome using bowtie2 (v2.3.5.1) to obtain unmapped sequences.

(2) Use Kraken2 (v2.1.2) to align the unmapped sequences against the human genome (GRCh38) and remove potential human sequences again.

(3) Annotate the microbial sequences based on the SAHMI pipeline to identify intratumoral microbiota co-localized with each cell subtype in the LUAD microenvironment, and analyze the association between intratumoral microbiota and each cell subtype in the TME using the Seurat package.

(4) Investigating the association between the host and microbiota at the single-cell resolution: First, we identified differentially expressed genes in host cells with and without microbial reads within each LUAD cancer cell subtype to generate a microbiota -related gene set from tumor tissues. Next, we performed the same comparison in LUAD adjacent normal tissues to obtain a microbiota-related gene set from non-tumor tissues. We then removed overlapping genes between the two sets to isolate the microbe-related gene set specific to cancer tissues. Finally, KEGG enrichment analysis was carried out on this refined gene set to identify microbiota-related pathways in LUAD.

**Validation of the association between IPRMS phenotype and immune cell infiltration based on single-cell data from the TISCH database**

We validated the IPRMS phenotypes (High-IPRMS and Low-IPRMS) at the single-cell level for four tumors: BRCA, HNSC, KIRC, and LUAD. Initially, we downloaded the scRNA-seq expression matrix and metadata from TISCH2, filtering out cells derived from peripheral blood mononuclear cells (PBMCs) and lymph nodes. We then used the “run_seurat” function from the “scAB” package (31) to standardize the scRNA-seq data, select highly variable genes, data normalization, principal component analysis, dimensionality reduction and clustering. Next, we applied the “Scissor” function (using default parameters) from the “Scissor” package (32) to associate the scRNA-seq data from various tumors with TCGA bulk data and IPRMS phenotypes. The Scissor algorithm classifies cells into Scissor+ (High-IPRMS phenotype) cells, Scissor- (Low-IPRMS phenotype) cells, and background cells (non-Scissor phenotype, as “0” cells). This algorithm can identify cells highly correlated to the IPRMS phenotypes in different cell types. It allows us to compare the differences in cell types between High-IPRMS and Low-IPRMS cell subclusters at the single-cell level. Using the odds ratio (OR) defined by Zheng et al. (33), we evaluated the enrichment of the IPRMS phenotype for each cell type. For a specific cell type, an OR > 1.5 indicates a stronger tendency for enrichment in the given IPRMS phenotype, while an OR < 0.5 suggests a weaker tendency to be distributed within that phenotype.

**The connection and distinction between TCGA intratumoral microbiota and gut microbiota**

Based on the gut microbiota data from Yu et al. (34), we qualitatively compared the genus-level microbial list with those of 14 TCGA tumors (BLCA, BRCA, ESCA, HNSC, KICH, KIRC, KIRP, LIHC, LUAD, LUSC, PRAD, STAD, THCA, and UCEC) and the combined TCGA_14cancers using a Venn diagram. Additionally, we conducted source tracking analysis of the TCGA intratumoral microbiota using the FEAST (fast expectation-maximization microbial source tracking) algorithm (R “FEAST” package) (35). Specifically, we used the gut microbiota of healthy controls published by Yu et al. as the “Source”, and the filtered TCGA intratumoral microbiota for abundance and prevalence, as well as the unfiltered original TCGA intratumoral microbiota (TCGA-paired cohort) as the “Sink”, to determine the contribution ratio of gut microbiota to intratumoral microbiota (the proportion of intratumoral microbiota derived from gut microbiota), thereby analyzing the association between intratumoral microbiota and gut microbiota, as well as the uniqueness of intratumoral microbiota.

**References**

1. Oksanen J, Simpson GL, Blanchet FG, Kindt R, Legendre P, Minchin PR, O'Hara RB, Solymos P, Stevens MHH, Szoecs E, Wagner H, Barbour M, Bedward M, Bolker B, Borcard D, Carvalho G, Chirico M, De Caceres M, Durand S, Evangelista HBA, FitzJohn R, Friendly M, Furneaux B, Hannigan G, Hill MO, Lahti L, McGlinn D, Ouellette M-H, Ribeiro Cunha E, Smith T, Stier A, Ter Braak CJF, Weedon J. 2022. vegan: Community Ecology Package, <https://CRAN.R-project.org/package=vegan>.

2. Forcino FL, Ritterbush KA, Stafford ES. 2015. Evaluating the effectiveness of the Mantel test and Procrustes randomization test for exploratory ecological similarity among paleocommunities. Palaeogeography, Palaeoclimatology, Palaeoecology 426:199-208. <https://doi.org/https://doi.org/10.1016/j.palaeo.2015.03.023>

3. Priya S, Burns MB, Ward T, Mars RAT, Adamowicz B, Lock EF, Kashyap PC, Knights D, Blekhman R. 2022. Identification of shared and disease-specific host gene-microbiome associations across human diseases using multi-omic integration. Nature Microbiology 7:780-795. <https://doi.org/10.1038/s41564-022-01121-z>

4. Love MI, Huber W, Anders S. 2014. Moderated estimation of fold change and dispersion for RNA-seq data with DESeq2. Genome Biology 15:550.

5. Liu S, Wang Y, Duan L, Cui D, Deng K, Dong Z, Wei S. 2024. Whole transcriptome sequencing identifies a competitive endogenous RNA network that regulates the immunity of bladder cancer. Heliyon 10:e29344. <https://doi.org/10.1016/j.heliyon.2024.e29344>

6. Li S-Y, Hammarlund JA, Wu G, Lian J-W, Howell SJ, Clarke RB, Adamson AD, Gonçalves CF, Hogenesch JB, Anafi RC, Meng Q-J. 2024. Tumor circadian clock strength influences metastatic potential and predicts patient prognosis in luminal A breast cancer. Proceedings of the National Academy of Sciences of the United States of America 121:e2311854121. <https://doi.org/10.1073/pnas.2311854121>

7. You B-H, Yoon J-H, Kang H, Lee EK, Lee SK, Nam J-W. 2019. HERES, a lncRNA that regulates canonical and noncanonical Wnt signaling pathways via interaction with EZH2. Proceedings of the National Academy of Sciences of the United States of America 116:24620-24629. <https://doi.org/10.1073/pnas.1912126116>

8. Cheng H-Y, Hsieh C-H, Lin P-H, Chen Y-T, Hsu DS-S, Tai S-K, Chu P-Y, Yang M-H. 2022. Snail-regulated exosomal microRNA-21 suppresses NLRP3 inflammasome activity to enhance cisplatin resistance. Journal For Immunotherapy of Cancer 10. <https://doi.org/10.1136/jitc-2022-004832>

9. Zhao Q, Xue J, Hong B, Qian W, Liu T, Fan B, Cai J, Ji Y, Liu J, Yang Y, Li Q, Guo S, Zhang N. 2020. Transcriptomic characterization and innovative molecular classification of clear cell renal cell carcinoma in the Chinese population. Cancer Cell International 20:461. <https://doi.org/10.1186/s12935-020-01552-w>

10. Huang K-B, Gui C-P, Xu Y-Z, Li X-S, Zhao H-W, Cao J-Z, Chen Y-H, Pan Y-H, Liao B, Cao Y, Zhang X-K, Han H, Zhou F-J, Liu R-Y, Chen W-F, Jiang Z-Y, Feng Z-H, Jiang F-N, Yu Y-F, Xiong S-W, Han G-P, Tang Q, Ouyang K, Qu G-M, Wu J-T, Cao M, Dong B-J, Huang Y-R, Zhang J, Li C-X, Li P-X, Chen W, Zhong W-D, Guo J-P, Liu Z-P, Hsieh J-T, Xie D, Cai M-Y, Xue W, Wei J-H, Luo J-H. 2024. A multi-classifier system integrated by clinico-histology-genomic analysis for predicting recurrence of papillary renal cell carcinoma. Nature Communications 15:6215. <https://doi.org/10.1038/s41467-024-50369-y>

11. Long M, Zhou Z, Wei X, Lin Q, Qiu M, Zhou Y, Chen P, Jiang Y, Wen Q, Liu Y, Li R, Zhou X, Yu H. 2022. A novel risk score based on immune-related genes for hepatocellular carcinoma as a reliable prognostic biomarker and correlated with immune infiltration. Frontiers In Immunology 13:1023349. <https://doi.org/10.3389/fimmu.2022.1023349>

12. Wang Y, Miao Z, Qin X, Yang Y, Wu S, Miao Q, Li B, Zhang M, Wu P, Han Y, Li B. 2023. Transcriptomic landscape based on annotated clinical features reveals PLPP2 involvement in lipid raft-mediated proliferation signature of early-stage lung adenocarcinoma. Journal of Experimental & Clinical Cancer Research : CR 42:315. <https://doi.org/10.1186/s13046-023-02877-w>

13. Ramakrishnan S, Cortes-Gomez E, Athans SR, Attwood KM, Rosario SR, Kim SJ, Mager DE, Isenhart EG, Hu Q, Wang J, Woloszynska A. 2024. Race-specific coregulatory and transcriptomic profiles associated with DNA methylation and androgen receptor in prostate cancer. Genome Medicine 16:52. <https://doi.org/10.1186/s13073-024-01323-6>

14. Liyanarachchi S, Li W, Yan P, Bundschuh R, Brock P, Senter L, Ringel MD, de la Chapelle A, He H. 2016. Genome-Wide Expression Screening Discloses Long Noncoding RNAs Involved in Thyroid Carcinogenesis. The Journal of Clinical Endocrinology and Metabolism 101:4005-4013.

15. DiGuardo MA, Davila JI, Jackson RA, Nair AA, Fadra N, Minn KT, Atiq MA, Zarei S, Blommel JH, Knight SM, Jen J, Eckloff BW, Voss JS, Rumilla KM, Kerr SE, Lam-Himlin DM, Bellizzi AM, Graham RP, Kipp BR, Jenkins RB, Halling KC. 2021. RNA-Seq Reveals Differences in Expressed Tumor Mutation Burden in Colorectal and Endometrial Cancers with and without Defective DNA-Mismatch Repair. The Journal of Molecular Diagnostics : JMD 23:555-564. <https://doi.org/10.1016/j.jmoldx.2021.01.008>

16. Han Y, Wang Y, Dong X, Sun D, Liu Z, Yue J, Wang H, Li T, Wang C. 2023. TISCH2: expanded datasets and new tools for single-cell transcriptome analyses of the tumor microenvironment. Nucleic Acids Research 51:D1425-D1431. <https://doi.org/10.1093/nar/gkac959>

17. Gao R, Bai S, Henderson YC, Lin Y, Schalck A, Yan Y, Kumar T, Hu M, Sei E, Davis A, Wang F, Shaitelman SF, Wang JR, Chen K, Moulder S, Lai SY, Navin NE. 2021. Delineating copy number and clonal substructure in human tumors from single-cell transcriptomes. Nature Biotechnology 39:599-608. <https://doi.org/10.1038/s41587-020-00795-2>

18. Wu SZ, Al-Eryani G, Roden DL, Junankar S, Harvey K, Andersson A, Thennavan A, Wang C, Torpy JR, Bartonicek N, Wang T, Larsson L, Kaczorowski D, Weisenfeld NI, Uytingco CR, Chew JG, Bent ZW, Chan C-L, Gnanasambandapillai V, Dutertre C-A, Gluch L, Hui MN, Beith J, Parker A, Robbins E, Segara D, Cooper C, Mak C, Chan B, Warrier S, Ginhoux F, Millar E, Powell JE, Williams SR, Liu XS, O'Toole S, Lim E, Lundeberg J, Perou CM, Swarbrick A. 2021. A single-cell and spatially resolved atlas of human breast cancers. Nature Genetics 53:1334-1347. <https://doi.org/10.1038/s41588-021-00911-1>

19. Chen Y-P, Yin J-H, Li W-F, Li H-J, Chen D-P, Zhang C-J, Lv J-W, Wang Y-Q, Li X-M, Li J-Y, Zhang P-P, Li Y-Q, He Q-M, Yang X-J, Lei Y, Tang L-L, Zhou G-Q, Mao Y-P, Wei C, Xiong K-X, Zhang H-B, Zhu S-D, Hou Y, Sun Y, Dean M, Amit I, Wu K, Kuang D-M, Li G-B, Liu N, Ma J. 2020. Single-cell transcriptomics reveals regulators underlying immune cell diversity and immune subtypes associated with prognosis in nasopharyngeal carcinoma. Cell Research 30:1024-1042. <https://doi.org/10.1038/s41422-020-0374-x>

20. Liu Y, He S, Wang X-L, Peng W, Chen Q-Y, Chi D-M, Chen J-R, Han B-W, Lin G-W, Li Y-Q, Wang Q-Y, Peng R-J, Wei P-P, Guo X, Li B, Xia X, Mai H-Q, Hu X-D, Zhang Z, Zeng Y-X, Bei J-X. 2021. Tumour heterogeneity and intercellular networks of nasopharyngeal carcinoma at single cell resolution. Nature Communications 12:741. <https://doi.org/10.1038/s41467-021-21043-4>

21. Yu Z, Lu W, Su C, Lv Y, Ye Y, Guo B, Liu D, Yan H, Mi H, Li T, Zhang Q, Cheng J, Mo Z. 2021. Single-Cell RNA-seq Identification of the Cellular Molecular Characteristics of Sporadic Bilateral Clear Cell Renal Cell Carcinoma. Frontiers In Oncology 11:659251. <https://doi.org/10.3389/fonc.2021.659251>

22. Zhang Y, Narayanan SP, Mannan R, Raskind G, Wang X, Vats P, Su F, Hosseini N, Cao X, Kumar-Sinha C, Ellison SJ, Giordano TJ, Morgan TM, Pitchiaya S, Alva A, Mehra R, Cieslik M, Dhanasekaran SM, Chinnaiyan AM. 2021. Single-cell analyses of renal cell cancers reveal insights into tumor microenvironment, cell of origin, and therapy response. Proceedings of the National Academy of Sciences of the United States of America 118. <https://doi.org/10.1073/pnas.2103240118>

23. Wu F, Fan J, He Y, Xiong A, Yu J, Li Y, Zhang Y, Zhao W, Zhou F, Li W, Zhang J, Zhang X, Qiao M, Gao G, Chen S, Chen X, Li X, Hou L, Wu C, Su C, Ren S, Odenthal M, Buettner R, Fang N, Zhou C. 2021. Single-cell profiling of tumor heterogeneity and the microenvironment in advanced non-small cell lung cancer. Nature Communications 12:2540. <https://doi.org/10.1038/s41467-021-22801-0>

24. Zilionis R, Engblom C, Pfirschke C, Savova V, Zemmour D, Saatcioglu HD, Krishnan I, Maroni G, Meyerovitz CV, Kerwin CM, Choi S, Richards WG, De Rienzo A, Tenen DG, Bueno R, Levantini E, Pittet MJ, Klein AM. 2019. Single-Cell Transcriptomics of Human and Mouse Lung Cancers Reveals Conserved Myeloid Populations across Individuals and Species. Immunity 50. <https://doi.org/10.1016/j.immuni.2019.03.009>

25. Gpertea PG. 2018. fqtrim: fqtrim release v0. 9.7 (v0. 9.7). Zenodo.

26. Kim D, Paggi JM, Park C, Bennett C, Salzberg SL. 2019. Graph-based genome alignment and genotyping with HISAT2 and HISAT-genotype. Nature Biotechnology 37:907-915. <https://doi.org/10.1038/s41587-019-0201-4>

27. Wood DE, Lu J, Langmead B. 2019. Improved metagenomic analysis with Kraken 2. Genome Biology 20:257. <https://doi.org/10.1186/s13059-019-1891-0>

28. Lu J, Breitwieser FP, Thielen P, Salzberg SL. 2017. Bracken: estimating species abundance in metagenomics data. PeerJ Computer Science 3:e104. <https://doi.org/10.7717/peerj-cs.104>

29. Laughney AM, Hu J, Campbell NR, Bakhoum SF, Setty M, Lavallée V-P, Xie Y, Masilionis I, Carr AJ, Kottapalli S, Allaj V, Mattar M, Rekhtman N, Xavier JB, Mazutis L, Poirier JT, Rudin CM, Pe'er D, Massagué J. 2020. Regenerative lineages and immune-mediated pruning in lung cancer metastasis. Nature Medicine 26:259-269. <https://doi.org/10.1038/s41591-019-0750-6>

30. Ghaddar B, Biswas A, Harris C, Omary MB, Carpizo DR, Blaser MJ, De S. 2022. Tumor microbiome links cellular programs and immunity in pancreatic cancer. Cancer Cell 40. <https://doi.org/10.1016/j.ccell.2022.09.009>

31. Zhang Q, Jin S, Zou X. 2022. scAB detects multiresolution cell states with clinical significance by integrating single-cell genomics and bulk sequencing data. Nucleic Acids Research 50:12112-12130. <https://doi.org/10.1093/nar/gkac1109>

32. Sun D, Guan X, Moran AE, Wu L-Y, Qian DZ, Schedin P, Dai M-S, Danilov AV, Alumkal JJ, Adey AC, Spellman PT, Xia Z. 2022. Identifying phenotype-associated subpopulations by integrating bulk and single-cell sequencing data. Nature Biotechnology 40:527-538. <https://doi.org/10.1038/s41587-021-01091-3>

33. Zheng L, Qin S, Si W, Wang A, Xing B, Gao R, Ren X, Wang L, Wu X, Zhang J, Wu N, Zhang N, Zheng H, Ouyang H, Chen K, Bu Z, Hu X, Ji J, Zhang Z. 2021. Pan-cancer single-cell landscape of tumor-infiltrating T cells. Science (New York, NY) 374:abe6474. <https://doi.org/10.1126/science.abe6474>

34. Lin Y, Lau HC-H, Liu Y, Kang X, Wang Y, Ting NL-N, Kwong TN-Y, Han J, Liu W, Liu C, She J, Wong SH, Sung JJ-Y, Yu J. 2022. Altered Mycobiota Signatures and Enriched Pathogenic Aspergillus rambellii Are Associated With Colorectal Cancer Based on Multicohort Fecal Metagenomic Analyses. Gastroenterology 163:908-921. <https://doi.org/10.1053/j.gastro.2022.06.038>

35. Shenhav L, Thompson M, Joseph TA, Briscoe L, Furman O, Bogumil D, Mizrahi I, Pe'er I, Halperin E. 2019. FEAST: fast expectation-maximization for microbial source tracking. Nature Methods 16:627-632. <https://doi.org/10.1038/s41592-019-0431-x>
